# Enhancing hydrogen permeation barrier performance of ErCo_2_ magnetic refrigeration material via surface oxide layer formation

**DOI:** 10.1038/s41467-026-71547-0

**Published:** 2026-04-07

**Authors:** Ya Xu, Keiji Oyoshi, Haruka Yoshikawa, Hiroyuki Takeya, Hiroshi Amekura, Takafumi D. Yamamoto, Yoshitaka Matsushita, Alexei A. Belik, Miyoko Tanaka, Akiko T. Saito, Koji Kamiya, Yoshihiko Takeda

**Affiliations:** 1 Research Center for Energy and Environmental Materials (GREEN), National Institute for Materials Science (NIMS), 3-13 Sakura, Tsukuba, Ibaraki, Japan; 2 Faculty of Advanced Engineering, Tokyo University of Science, 6-3-1 Niijyuku, Katsushika, Tokyo, Japan; 3 Research Center for Materials Nanoarchitectonics (MANA), National Institute for Materials Science (NIMS), Namiki 1-1, Tsukuba, Ibaraki, Japan; 4 Research Network and Facility Services Division, National Institute for Materials Science (NIMS), Sengen 1-2-1, Tsukuba, Ibaraki, Japan; 5 Research Center for Magnetic and Spintronic Materials, National Institute for Materials Science (NIMS), 3-13 Sakura, Tsukuba, Ibaraki, Japan; 6 Present Address: Research Network and Facility Services Division, National Institute for Materials Science (NIMS), Tsukuba, Japan; 7 Present Address: Mitsubishi Kakoki Kaisha, Ltd., Kawasaki, Kanagawa, Japan; 8 Present Address: Department of Materials Science and Technology, Tokyo University of Science, Tokyo, Japan

**Keywords:** Magnetic properties and materials, Hydrogen storage

## Abstract

The ErCo_2_ intermetallic compound exhibits a significant magnetocaloric effect at approximately 32 K and has potential applications as a magnetic refrigeration material for hydrogen liquefaction. However, exposure to a hydrogen atmosphere may lead to hydride formation, which weakens the magnetocaloric effect. Thus, preventing hydrogen permeation into ErCo_2_ is crucial. Herein, we enhance the hydrogen permeation barrier (HPB) performance of ErCo_2_ particles by using electroless Cu plating followed by oxidation treatment to form a CuO layer with a thickness of a few micrometers. In experiments, ErCo_2_ particles, with a　1.5- to 5-µm-thick CuO surface layer, exhibited a large magnetic entropy change of 24 J kg⁻¹ K⁻¹ even after exposure to a H_2_ atmosphere at 1.27 MPa and 296 K for 7 d. Experimental analyses and first-principles calculations revealed the potential of CuO as an HPB material for magnetic refrigeration.

## Abstract (web summary)

Hydrogen permeation weakens magnetocaloric effect of ErCo_2_. This study reports that forming a thin CuO layer via electroless Cu plating followed by oxidation can significantly enhance the hydrogen permeation barrier performance of ErCo_2_ particles.

## Introduction

Hydrogen liquefaction is crucial for its transportation and storage^1–3^. Magnetic refrigeration offers a more energy-efficient alternative to conventional gas compression/expansion refrigeration technology^4–7^, and the efficiency of such refrigeration systems can be further improved using magnetic refrigeration materials with large magnetocaloric effects. Numerous magnetic refrigeration materials with large magnetocaloric effects have been developed, such as DyNi_2_^8^, ErCo_2_^9–11^, ErNi_2_^12^, Dy_1-x_Er_x_Ni_2_^13^, HoB_2_^14^, and HoAl_2_^15^. However, the development of magnetic refrigeration materials with a large magnetic entropy change (∆*S*_*M*_) over a broad temperature range (77–20 K) is challenging^16–20^. A layered composite magnetic refrigerant of HoNi_2_, DyNi_2_, and TbNi_2_ has been developed and obtained an average *∆S*_*M*_ value of 4.7 J kg^–1^ K^–1^ under a 2 T field change in a broad temperature range of 7.5–53.4 K^21^. ErCo_2_ exhibits a *∆S*_*M*_ > 20 J kg^–1^ K^–1^ and an adiabatic temperature change (Δ*T*_ad_) of ~10 K under a 5 T field change at the Curie temperature of ~32 K^9–11^, making it suitable for hydrogen liquefaction. Recent strategies of alloying ErCo_2_ with Ni, Al, or Fe have expanded the available temperature range to 20–77 K^22^.

The shape optimization of magnetic materials is important for maximizing the heat transfer between the magnetic material and heat exchange fluid within the active magnetic regenerative refrigeration (AMRR) system^6,7^. Magnetic refrigeration materials with a spherical shape (diameter of 200–500 μm) are expected to increase the cooling efficiency of refrigeration systems because such particles can be densely packed in the system, while retaining a certain amount of gaps between them for the flow of heat exchange fluid^23^. Currently, AMRR systems use a helium gas atmosphere rather than a hydrogen gas atmosphere to liquefy hydrogen via helium gas flow^6^.

The AMRR system could be simplified, and the energy efficiency could be significantly improved if magnetic refrigeration materials could be placed directly in a hydrogen atmosphere. However, ErCo_2_ is prone to hydride formation upon hydrogen exposure^24–26^, leading to particle fragmentation and loss of the magnetocaloric effect. Since the magnetic refrigeration materials must be exposed to a hydrogen atmosphere at room temperature for a few days each year during the routine maintenance period of magnetic refrigeration systems, it is imperative to develop ErCo_2_-based materials with superior hydrogen permeation barrier (HPB) performance that are effective not only at cryogenic temperatures but also at room temperature.

Previous studies have explored HPB coatings for steel materials to prevent hydrogen embrittlement and leakage in nuclear power plants^27–29^. Oxides such as Cr_2_O_3_-SiO_2_, Al_2_O_3_, Y_2_O_3_, Er_2_O_3_, La_2_O_3_, and SiO_2_ have been investigated owing to their low hydrogen solubility and permeability^30–32^. They are typically coated via a variety of techniques: hot-dipping, thermal spraying, plasma-spraying, physical vapor deposition (PVD), and chemical vapor deposition. We attempted to use hot-dipping, thermal spraying, and barrel PVD methods to form a Cr_2_O_3_ or Al₂O₃ layer onto the ErCo₂ particles. However, particle aggregation is difficult to prevent via thermal spraying, and the coating uniformity is hard to control on the small ErCo_2_ particles via the PVD method.

Compared to these coating methods, the formation of a CuO layer by copper plating followed by oxidation treatment offers great advantages in terms of cost and efficiency. CuO has not previously been recognized as an HPB material, and this study has demonstrated for the first time that it possesses sufficient HPB properties near room temperature.

Herein, we report our work to synthesize ErCo₂ magnetic refrigeration materials with excellent HPB performance using well-established techniques, i.e., preparing ErCo_2_ particles with diameters of 212–355 µm using an electrode induction melting gas atomization (EIGA) process^33–36^, forming a Cu layer on the ErCo_2_ particles using electroless plating, and forming a CuO layer using the subsequent oxidation treatment. To compare the HPB performance of different oxide layers, we formed a Co(Er) oxide layer through oxidation treatment alone and a CuO layer through a combination of electroless Cu plating and oxidation treatment on the particle surface. The HPB performance of the particles with these layers was evaluated using a Sieverts-type instrument (Fig. S1) at an initial H_2_ pressure of ~1.27 MPa and 296 K. The effects of the oxide layers on the magnetic properties of ErCo₂ particles were evaluated using a Quantum Design SQUID magnetometer. The ErCo_2_ particles with a Co(Er) oxide layer significantly improved the HPB performance, while particles with a CuO layer further improved it, almost completely blocking hydrogen permeation for 7 d under the abovementioned hydrogen exposure conditions. The CuO layer was analyzed via synchrotron X-ray diffraction (SXRD), scanning electron microscopy (SEM), and transmission electron microscopy (TEM). Time-of-flight secondary ion mass spectrometry (ToF-SIMS) provided experimental evidence that hydrogen is blocked at the surface. Density functional theory (DFT) calculations revealed that H_2_ is energetically favored for physisorption on the CuO surface and that its dissociation to H atoms must overcome an energy barrier of >20 kJ/mol, while diffusion of the H atom into CuO requires overcoming an even larger energy barrier ( > 300 kJ/mol). The DFT calculation results agreed with the experimental results, demonstrating the potential of CuO as an HPB material.

## Results and discussion

### HPB performance and magnetic properties

**Fig. 1:**
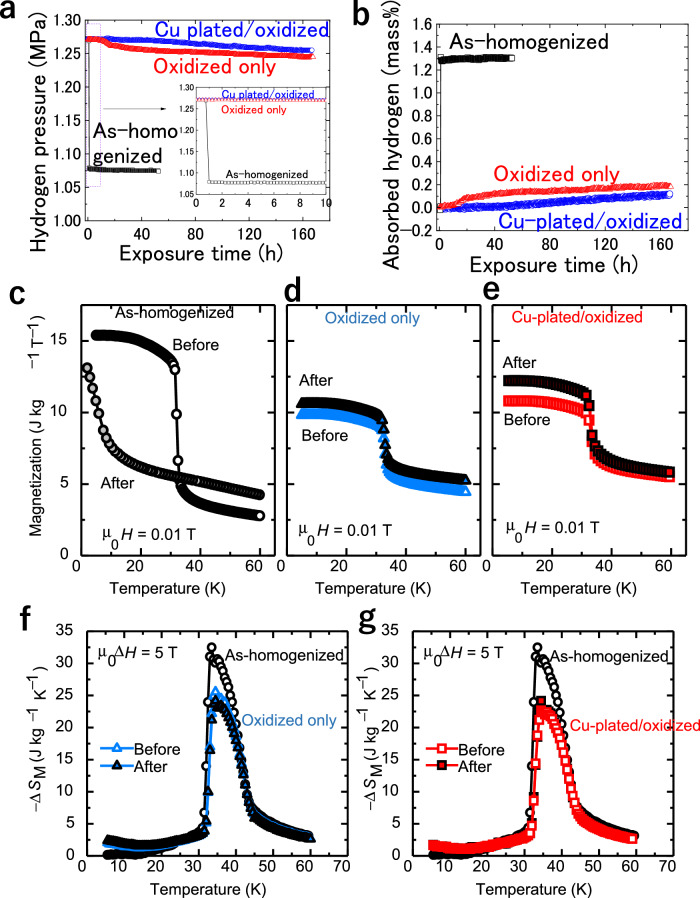
Hydrogen permeation barrier performance and magnetic properties of the ErCo_2_ particles. **a** Hydrogen pressure changes during the hydrogen exposure test for the as-homogenized, oxidized only, and Cu-plated/oxidized ErCo_2_ particles. The inset shows the hydrogen pressure change during the initial ten hours. **b** Corresponding absorbed hydrogen amount calculated from the pressure changes. The hydrogen exposure test started with an initial hydrogen pressure in the cell of 1.27 MPa at approximately 296 K. **c** Temperature dependence of magnetization at 0.01 T for the as-homogenized ErCo_2_ particles. **d** Temperature dependence of magnetization at 0.01 T for the oxidized ErCo_2_ particles before and after the hydrogen exposure test. **e** Temperature dependence of magnetization at 0.01 T for the Cu-plated/oxidized ErCo_2_ particles before and after the hydrogen exposure test. **f** Magnetic entropy change (∆*S*_M_) for *μ*_0_ *∆H* = 5 T of the as-homogenized and oxidized ErCo_2_ particles before and after the hydrogen exposure test. **g** Magnetic entropy change (∆*S*_M_) for *μ*_0_ *∆H* = 5 T of the as-homogenized and Cu-plated/oxidized ErCo_2_ particles before and after the hydrogen exposure test (∆*S*_M_ is not shown for the as-homogenized particles after the hydrogen exposure test). Source data are provided as a Source Data file.

The effects of different oxide layers on the HPB performance of ErCo_2_ particles were investigated by comparing three types of samples: (1) particles after homogenization at 1123 K for 7 d in an Ar atmosphere (referred to as “as-homogenized”), (2) the homogenized particles after oxidation at 773 K for 0.5 h in air (referred to as “oxidized”), and (3) the homogenized particles after electroless Cu plating and subsequent oxidation at 773 K for 0.5 h in air (referred to as “Cu-plated/oxidized”). Figure 1a shows the hydrogen pressure changes over time for these samples under an initial hydrogen pressure of ∼1.27 MPa at 296 K, and Fig. 1b shows the corresponding absorbed hydrogen amounts calculated from the pressure changes.

For the as-homogenized ErCo_2_ particles, the hydrogen pressure rapidly decreased from 1.27 to 1.08 MPa within ∼1 h, corresponding to an absorbed hydrogen amount of ~1.311 mass% of the sample weight—comparable to that observed in typical hydrogen storage alloys^37,38^. Stereomicroscopy reveals that the ErCo_2_ particles pulverized after hydrogen exposure (Fig. S2a, S2b), which could be due to the large amount of absorbed hydrogen in ErCo_2_.

In contrast, the oxidized ErCo_2_ particles underwent slower hydrogen absorption, achieving a significantly reduced absorbed hydrogen amount. The initial hydrogen pressure of 1.271 MPa started to decrease only after 14 h and reached ~1.245 MPa after 167.5 h, corresponding to an absorbed hydrogen amount of 0.182 mass%. Stereomicroscopy images (Fig. S2c, S2d) showed that most of the particles retained their shape, and only a few were pulverized. These results demonstrate that the oxidation treatment substantially improved the HPB performance of ErCo_2_ by restricting hydrogen permeation.

Further improvement was observed for the Cu-plated/oxidized particles. The hydrogen pressure (initially 1.272 MPa) remained almost unchanged, decreased slightly after 60 h, and stabilized at 1.255 MPa after 166.75 h. The corresponding absorbed hydrogen amount was 0.117 mass%, indicating even slower absorption than that upon oxidation alone. Stereomicroscopy confirmed that most particles retained their shape after hydrogen exposure (Fig. S2e, S2f). The as-homogenized ErCo_2_ particles exhibited low HPB performance under 1.27 MPa hydrogen at room temperature, while oxidation significantly improved the HPB properties. Moreover, Cu plating followed by oxidation further improved the HPB performance, effectively preventing hydrogen permeation for over 100 h.

Pressure–composition–temperature (PCT) measurements were performed for the as-homogenized, oxidized, and Cu-plated/oxidized ErCo_2_ particles (Fig. S3). For the as-homogenized particles, no obvious hydrogen absorption was observed up to 4 MPa at 303 K, whereas the amount of hydrogen absorption reached approximately 1.3 mass% at 333 K. The PCT results are consistent with the hydrogen exposure test results, considering that the PCT measurement was completed in less than an hour (0.57 h) at 303 K and hydrogen absorption commenced in the as-homogenized particles during the hydrogen exposure test (Fig. 1a). In addition, the amount of hydrogen absorption obtained from the PCT measurement at 333 K agrees well with the value of 1.311 mass% obtained from the hydrogen exposure test. The absorbed hydrogen maintained approximately 1 mass% even after reducing the pressure to a low level ( < 0.005 MPa), suggesting that hydrogen absorbed by ErCo_2_ does not easily desorb.

For the oxidized particles, no obvious hydrogen absorption was observed at 303 K, and only a small amount of hydrogen absorption ( ~ 0.2 mass%) was observed at 333 K (Fig. S3b). For the Cu-plated/oxidized particles, no apparent hydrogen absorption was observed at both 303 and 333 K (Fig. S3c). These PCT measurements are consistent with those of the hydrogen exposure test (Fig. 1a, b), indicating that the HPB performance was improved by the oxidation and further enhanced by Cu-plated/oxidation treatment.

Furthermore, we performed long-term hydrogen exposure tests at room temperature up to 28 days for evaluating long-term hydrogen permeation stability of oxidized only and Cu-plated/oxidized ErCo_2_ particles. Particles after different Cu plating times (0.17 and 1.5 h) were used for examining the influence of CuO layer thickness on HPB performance (Fig. S4). For all samples, the decrease in hydrogen pressure throughout the test period was less than 2%, indicating an extremely slow hydrogen absorption rate. This demonstrated that both oxidized and Cu-plated/oxidized ErCo₂ exhibit excellent long-term HPB stability. Among these, the Cu-plated/oxidized particles showed slightly superior HPB characteristics compared to the oxidized particles. Furthermore, samples with shorter Cu plating time (0.17 h) exhibited HPB characteristics no less than those with long Cu plating time (1.5 h) (Table S1). As the thickness of the CuO layer formed on the sample with shorter Cu plating time (0.17 h) was approximately 1.5 μm (Fig. S5), this result suggests that a 1.5 μⅿ-thick CuO layer can provide a sufficient HPB effect.

The effect of the Cu plating–oxidation treatment on the magnetic properties of ErCo_2_ was investigated by measuring the M–T curves under varying field up to 5 T for the as-homogenized, oxidized, and Cu-plated/oxidized samples before and after H_2_ exposure test (Fig. S6) to calculate the magnetic entropy change ($\triangle {S}_{M}$) using Eq. 3. Figure 1c, d show the M–T curves of the as-homogenized, oxidized, and Cu-plated/oxidized particles, measured at 0.01 T before and after hydrogen exposure. In the as-homogenized particles, magnetization sharply increased at ~32 K (the Curie temperature) owing to the first-order ferrimagnetic transition^9,10^. After hydrogen exposure, this transition was absent, replaced by a rapid magnetization increase below 10 K—suggesting ErCo_2_ conversion to hydride, which significantly altered the magnetic properties. Conversely, the oxidized and Cu-plated/oxidized particles retained a clear magnetization increase at ~32 K, both before and after hydrogen exposure, confirming that the Cu plating–oxidation treatment blocks hydrogen permeation and preserves the magnetic properties of ErCo_2_.

Figure 1f, g present ∆*S*_M_ for the as-homogenized, oxidized, and Cu-plated/oxidized samples under μ_0_∆*H* = 5 T. The maximum ∆*S*_M_ was ~33 J kg⁻¹ K⁻¹ for the as-homogenized particles, ~26 J kg⁻¹ K⁻¹ after the oxidation treatment, and ~24 J kg⁻¹ K⁻¹ after the Cu plating−oxidation treatment. The temperature range of large ∆*S*_M_ (32–42 K) remained unchanged. After hydrogen exposure, both ∆*S*_M_ value and temperature range remained stable for the oxidized and Cu-plated/oxidized samples. The reduction in Δ*S*_M_ is due to the decrease in the weight fraction of ErCo_2_ main phase in the ErCo_2_ particle after Cu plating and/or oxidation treatments. As the Δ*S*_M_ data in the present study are shown in units of J per K per total mass of ErCo_2_ particles, their magnitude decreases proportionally to the weight fraction of the ErCo_2_ phase. From the microstructural analysis results shown in Fig. 3, we obtained an average thickness of each phase by measuring 3–5 particles and evaluated the weight fraction of each phase in the ErCo_2_ particle: the innermost ErCo_2_ phase, the Er–Co–O layer, the Co–O layer, and the outermost CuO layer (Fig. S7). The weight fraction of the ErCo_2_ phase decreased by approximately 29% after Cu plating and oxidation, which roughly corresponds to the 30% decrease in magnetic entropy change. For the oxidized-only sample, we also confirmed that the decrease in the weight fraction of the ErCo_2_ phase corresponds to the decrease in magnetic entropy via the same process (Fig. S8). These results indicate that while the magnetocaloric effect per unit weight after oxidation or Cu plating–oxidation treatments is smaller, it maintains a high intensity of this effect and effectively prevents hydrogen-induced deterioration of the magnetic properties of ErCo_2_.

### Characterization of ErCo_2_ and surface oxide layer

**Fig. 2:**
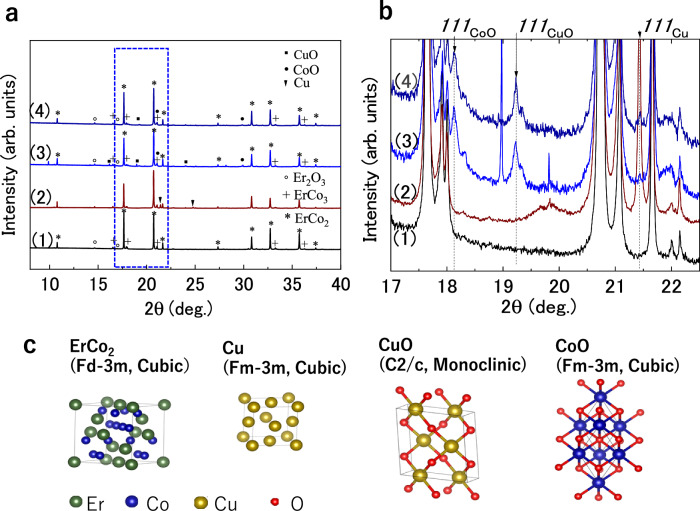
Structural characterization of the ErCo₂ particles via synchrotron X-ray diffraction. **a** XRD patterns of the as-homogenized (1), Cu-plated (2), and Cu-plated/oxidized ErCo_2_ particles (3) and the particles after the hydrogen exposure test (4). **b** Magnified view of the 2θ = 17°–22.5° range, indicating the presence of the *111*_*Cu*_ peak after Cu plating, along with *111*_*CuO*_ and *111*_*CoO*_ peaks after oxidation. **c** Unit lattices of the identified phases: ErCo_2_, Cu, CuO, and CoO. Source data are provided as a Source Data file.

The changes in the crystal structure of the ErCo_2_ particles during the Cu plating–oxidation treatment were evaluated through SXRD. Figure 2a presents the SXRD profiles of the as-homogenized, Cu-plated, and Cu-plated/oxidized ErCo_2_ particles before and after hydrogen exposure. For the as-homogenized particles, most peaks corresponded to the ErCo_2_ phase (cubic, with space group symmetry ${Fd}\bar{3}m$), accompanied by weak peaks indexed to the ErCo_3_ (hexagonal, $R\bar{3}{{\rm{m}}}$) and HT- & LT-Er_2_O_3_ (${Ia}\bar{3}{and}$ $C2/m$) phases. Qualitative analysis using the reference intensity ratio revealed the mass percentages of ErCo_2_, ErCo_3_, and Er_2_O_3_ (Sum of HT- and LT-phase values) to be 94%, 5.5%, and 0.5%, respectively (Fig. S9). The trace amounts of the Er_2_O_3_ phase likely resulted from the formation of a natural surface oxide layer upon air exposure, as confirmed by cross-sectional analyses via SEM and energy-dispersive X-ray spectroscopy (EDS; Fig. S10).

After Cu plating, in addition to the aforementioned phases, trace amounts of the metallic Cu phase (cubic, ${Fm}\bar{3}m$) were detected. Following the oxidation of the Cu-plated sample, ErCo_2_ remained as the main phase, while the metallic Cu phase disappeared and trace amounts of CuO ($C2/c$) and CoO (cubic, ${Fm}\bar{3}m$) were observed. A magnified view of 2*θ* = 17°–22.5° clearly revealed the presence of a 111_Cu_ peak after Cu plating and 111_CuO_ and 111_CoO_ peaks after oxidation (Fig. 2b). After hydrogen exposure, the SXRD profiles of the Cu-plated/oxidized particles remained unchanged, indicating that the oxide phases persist even after hydrogen exposure at 1.27 MPa and ~296 K. This reveals that CuO is not reduced to its metallic state under these conditions, which is consistent with previous reports suggesting that CuO is difficult to reduce at room temperature^39,40^. The unit lattice schematics of the identified phases are shown in Fig. 2c. These results indicate that small amounts of oxides (CuO, CoO, and LT-Er_2_O_3_) were formed after Cu plating and the subsequent oxidation; however, the primary phase remained as ErCo_2_.

**Fig. 3:**
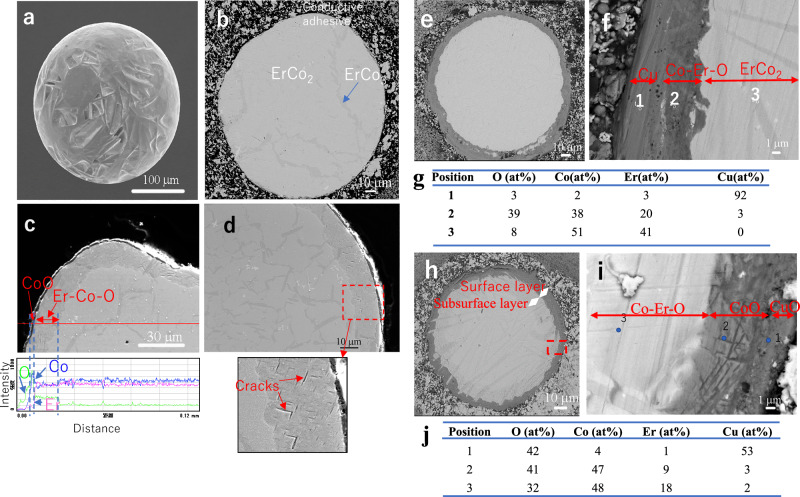
Analysis of surface and cross-sectional morphologies of the ErCo_2_ particles via SEM. **a** SE images of the ErCo_2_ particle produced by atomization after homogenization at 1173 K for 7 days in an Ar gas atmosphere. **b** BSE image of the cross-section of the as-homogenized particle. **c** Cross-sectional SE image of the oxidized ErCo_2_ particle and the results of EDS linear component analysis. **d** Cross-sectional SE image of the oxidized ErCo_2_ particle with an enlarged view showing small cracks in the surface layer. **e** Cross-sectional BSE image of the Cu-plated particle. **f** Cross-sectional BSE image of the surface layer of Cu-plated particle. **g** EDS results for the positions marked in (**f**). **h** Cross-sectional BSE image of the Cu-plated/oxidized ErCo_2_ particle. **i** Cross-sectional BSE image of the surface layer region in **h**. **j** EDS results for the positions marked in (**i**).

The surface and cross-sectional morphologies of the as-homogenized ErCo_2_ particles were examined through SEM. Figure 3a shows a secondary electron (SE) image revealing surface irregularities, and Fig. 3b shows a backscattered electron (BSE) image indicating a largely uniform composition, except for some small dark contrast areas. EDS identified these areas as Co-rich regions (Fig. S10), which likely corresponded to the ErCo_3_ phase, as supported by the SXRD results. EDS line analysis along the particle diameter revealed a thin Er- and O-enriched surface layer, suggesting the presence of a natural Er oxide film (Fig. S10).

ErCo_2_ particles with Er and Co oxide surface layers were prepared via the oxidation treatment. Figure 3c shows the SE image and EDS line analysis results of an oxidized particle cross-section, revealing a two-layered structure. The outermost layer (1–2 μm thick) exhibited a dark contrast and consisted mainly of Co oxide. Beneath it, a 5- to 20-μm-thick intermediate layer appeared brighter than the outermost layer but darker than the interior. EDS indicated the presence of Er and Co oxides in this layer, suggesting the partial oxidation of ErCo_2_. Cracks were observed within some areas of this oxide layer (Fig. 3d), indicating its brittleness and lack of densification.

ErCo_2_ particles with a CuO surface layer were prepared via the Cu plating–oxidation treatment. To ensure uniform plating, a brief pretreatment with dilute hydrochloric acid was conducted before Cu deposition, forming an oxide layer with a larger atomic ratio of Co/Er compared with that of the subsurface region (Fig. S11). The dissolution of Er was more pronounced than that of Co, leading to a higher Co concentration on the surface. After Cu plating, a 10 to 15-μm-thick dark contrast layer was formed (Fig. 3e, f). EDS identified Cu as the dominant element in the outermost region, with Co, O, and Er in the underlying region within this layer (Fig. 3g). The Co/Er ratio in the underlying region (1.9) was higher than that in the adjacent interior (1.2), likely because of the acid pretreatment.

Subsequent oxidation of the Cu-plated particles led to further structural changes. The subsurface layer formed beneath the surface layer with dark contrast exhibited an intermediate contrast (Fig. 3h, i). EDS analysis (Fig. 3j) confirmed that the outermost layer primarily consisted of CuO, whereas the underlying layer contained CoO with minor Er oxides. The subsurface layer contained Co, Er, and O, with an O concentration (32 at%) lower than that in the surface layer (41 at%) but significantly higher than that in the interior (Fig. 3g), indicating the presence of distinct Er and Co oxides.

**Fig. 4:**
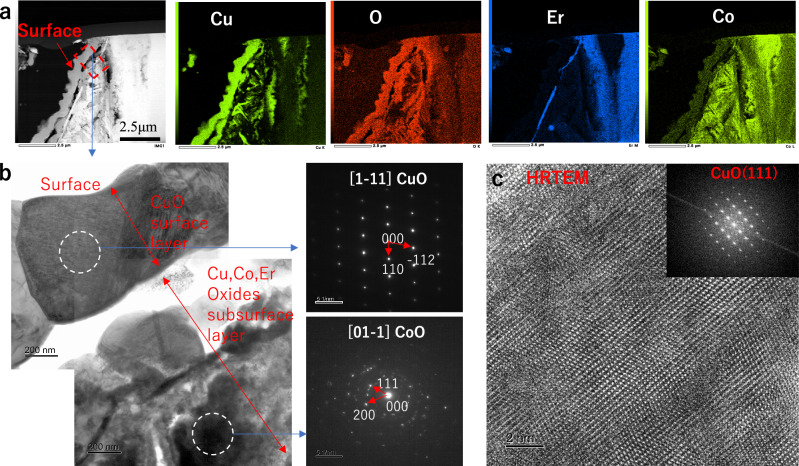
Microstructural characterization of the surface layer via TEM. **a** HAADF-STEM image of the surface layer area of the Cu-plated/oxidized ErCo_2_ particle and the corresponding EDS elemental mapping images of Cu, O, Er, and Co. **b** TEM image of the marked region in **a** and SAED patterns for the outer and subsurface layers. **c** HRTEM image of the CuO outer layer; the inset shows the corresponding FFT pattern.

The surface-layer structures of the Cu-plated/oxidized particles were further analyzed by TEM. Figure 4a presents a high-angle annular dark-field scanning TEM (HAADF-STEM) image with EDS elemental mapping, which reveals a continuous CuO outer layer on top of an underlying Co-rich oxide layer with minor Cu and Er oxides. Figure 4b shows the TEM image of the selected region, with selected-area electron diffraction (SAED) patterns confirming the crystallinity of CuO and CoO. Figure 4c presents the high-resolution TEM (HRTEM) image and the fast Fourier transform (FFT) pattern of the CuO layer, indicating well-crystallized CuO(111). These results indicate that a well-crystallized CuO layer with large crystallites forms on the outer surface, whereas the subsurface Co-, Er-, and Cu-oxide layers exhibit some missing regions, which could be due to a weak interface between the subsurface oxide layer and the outmost surface of the CuO layer.

### Mechanism of HPB performance improvement

**Fig. 5:**
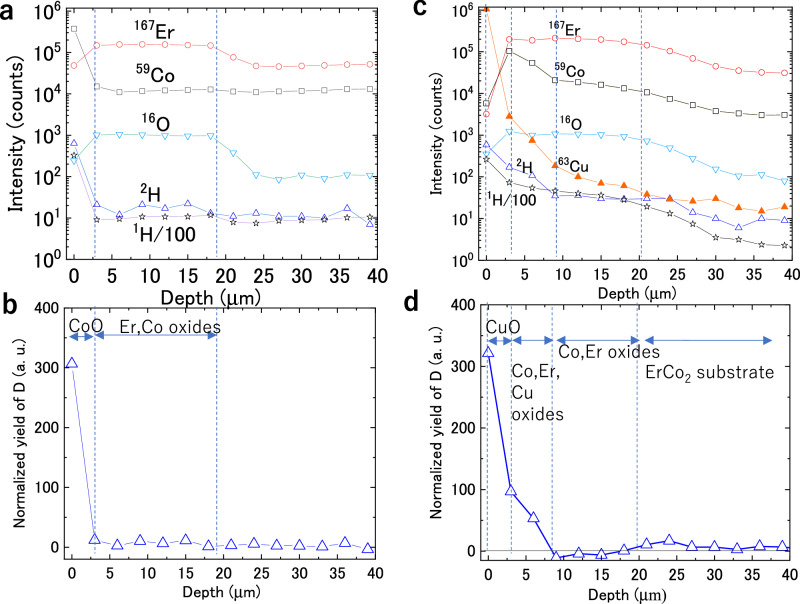
Analysis of hydrogen permeation behavior via time-of-flight secondary ion mass spectrometry (ToF-SIMS). **a** ToF-SIMS depth profiles of ^167^Er, ^59^Co, ^16^O, ^2^H, and ^1^H/100 in the surface layer of the oxidized ErCo_2_ particles after D_2_ exposure. **b** Normalized yield of D of the oxidized ErCo_2_ particles obtained by excluding the background H signal: D yield = detected ^2^H signal − detected ^1^H signal/100. **c** ToF-SIMS depth profiles of ^167^Er, ^59^Co, ^16^O, ^63^Cu, ^2^H, and ^1^H/100 in the surface layer of the Cu-plated/oxidized ErCo_2_ particles after D_2_ exposure. **d** Normalized yield of D of the Cu-plated/oxidized ErCo_2_ particles. Note that the vertical axis in (**a**) and (**c**) is the number of secondary ions counted and does not correspond to the elemental composition ratio. Source data are provided as a Source Data file.

Hydrogen permeation involves adsorption, dissociation, and diffusion^41–43^. To determine the rate-limiting step for ErCo_2_ with the CuO surface layer, ToF-SIMS was conducted to evaluate the deuterium distribution in the surface oxide layer after exposure to D_2_. Figure 5a shows the ToF-SIMS depth profiles of ^167^Er, ^59^Co, ^16^O, ^2^H, and ^1^H/100 in the oxidized ErCo_2_ particles after D_2_ exposure. In the outermost ~3 µm, the peak intensity of ^59^Co was higher, that of ^167^Er was lower, and that of ^16^O was higher than that of the deeper layers ( ~ 40 µm), suggesting the formation of a Co-enriched oxide layer, consistent with SEM results (Fig. 3). Beneath this layer, up to ~20 µm, the peak intensities of ^167^Er, ^59^Co, and ^16^O were relatively constant, whereas with further increase of the depth, the peak densities of ^167^Er and ^16^O decreased, while that of ^59^Co remained constant, indicating the presence of an Er-rich oxide layer—again consistent with the SEM results (Fig. 3). The calculated D concentration in the CoO layer significantly reduced, reaching nearly zero at the interface of the next layer (Fig. 5b), suggesting that deuterium was primarily blocked by the CoO layer.

Figure 5c shows the ToF-SIMS depth profiles of ^167^Er, ^59^Co, ^16^O, ^63^Cu, ^2^H, and ^1^H/100 in the Cu-plated/oxidized ErCo_2_ particles after D_2_ exposure. The outermost surface exhibited strong signals for ^63^Cu, ^2^H, and ^1^H/100, along with a relatively high ^16^O intensity. Moreover, the ^59^Co intensity was at similar levels, and the ^167^Er signal was weak compared to their intensities at deeper layers ( ~ 40 μm). With an increase in depth, the intensities of the ^63^Cu, ^2^H, and ^1^H/100 signals rapidly decreased, while those of the ^167^Er, ^59^Co, and ^16^O signals increased, reaching a maximum at ~4 μm. Beyond this depth, the ^59^Co signal intensity gradually declined, while the ^167^Er and ^16^O signal intensities remained constant up to ~20 μm, after which all intensities declined. These findings align with the SXRD, SEM, and TEM results, confirming the formation of a CuO outer layer (several micrometers thick) followed by mixed Cu–Co–Er oxide layers. Figure 5d shows that the D concentration of the CuO layer significantly decreased and reached zero in the underlying mixed oxide layer. This suggests that the CuO layer prevents hydrogen permeation, with additional blocking from the mixed Cu–Co–Er oxides.

These results indicate that both CoO and CuO have a significant inhibition effect on hydrogen permeation. However, as shown in Fig. 1, the oxidized particles with the Co(Er)O surface layer did not allow sufficient hydrogen permeation blocking. This was likely due to the presence of fine cracks in the Co(Er)O oxide layer formed on some particles (Fig. 3d), which resulted in hydrogen permeation into particles. In contrast, the CuO layer was denser (Fig. 4) and exhibited a stronger hydrogen blocking effect.

**Fig. 6:**
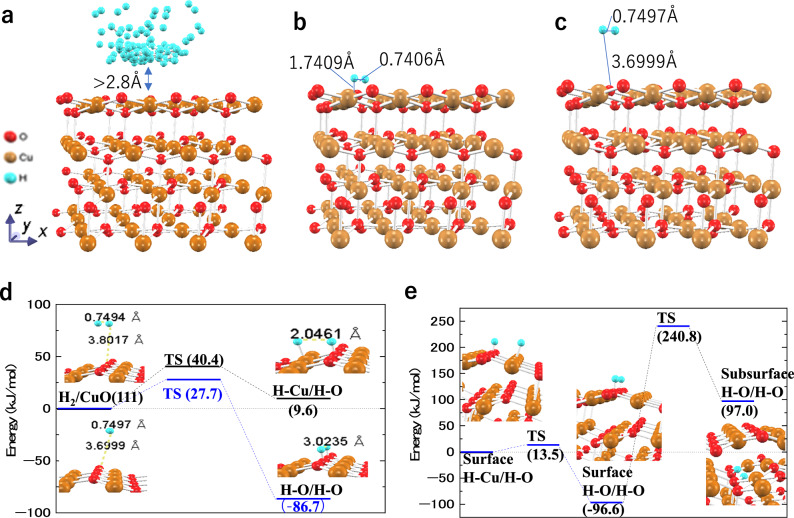
Analysis of hydrogen adsorption, dissociation, and diffusion on CuO(111) surface via DFT calculation. **a** Optimized positions of H_2_ molecules at all initial adsorption sites (shown in Fig. S4a), showing that all H_2_ molecules moved away from the CuO(111) surface, to a distance exceeding 2.6 Å. **b** H_2_ set at the initial Cu^4^–Cu^4^ bridge site ⓾ shown in Fig. S12a, with an orientation parallel to the surface and vertical to the bridge direction. **c** Optimized positions of the H_2_ molecule at the initial adsorption site shown in (**b**). **d** Activation barriers for H_2_ dissociation to form two H–O bonds (H-O/H-O) or one H–Cu and one H–O bond (H-Cu/H-O) (TS: transition state). **e** Activation barriers for H-atom diffusion from H–Cu/H–O to H–O/H–O on the surface and from surface H–O/H–O to subsurface H–O/H–O.

DFT calculations were performed to further investigate the hydrogen adsorption, dissociation, and diffusion on the CuO(111) surface. The adsorption energies and optimized geometries were calculated for an H_2_ molecule at various sites on CuO(111), including three-coordinated O (hereafter denoted as O^3^) and Cu (as Cu^3^), four-coordinated O (as O^4^) and Cu (as Cu^4^), 11 bridge sites, and 8 hollow sites (Fig. S12a). Five molecular orientations were considered: one perpendicular to the CuO(111) surface, two parallel, and two tilted approximately 45° toward the z-axis from the two parallel orientations (Fig. S12c). Initially, H_2_ was positioned ~1.5 Å from the surface, with an H–H bond length of 0.74 Å. After optimization, the H_2_ molecule moved away from the surface (2.8–6 Å) (Fig. 6a), with minimal bond length change (0.74–0.75 Å) and low adsorption energies (−2.71–2.36 kJ/mol) (Table S2_1 and S2_2). For example, a H_2_ molecule initially at the Cu^4^–Cu^4^ bridge site (shown as ⑩ Bridge-Cu^4^-Cu^4^ in Fig. S12a) and vertical to the bridge direction was set 1.7409 Å from the nearest surface atom (Fig. 6b) and moved to 3.6999 Å from the nearest surface atom after optimization. In contrast, the H–H bond length changed only from 0.7406 to 0.7497 Å (Fig. 6c), and the adsorption energy was only 0.68 kJ/mol (Table S2_1). The results indicate that H_2_ prefers physisorption and requires an energy barrier for chemisorption and dissociation on the CuO(111) surface.

The activation barriers for H_2_ dissociation at the optimized sites were calculated for two cases: (i) both H atoms adsorbed onto adjacent O^3^ atoms (forming two H–O bonds) and (ii) one H atom adsorbed onto O^3^ and the other onto Cu^3^ (forming H–O and H–Cu bonds). The activation barriers ranged from 27.7 to 152.4 kJ/mol for H–O bond formation and from 40.4 to 63.8 kJ/mol for H–O and H–Cu bond formation (Fig. S13). The lowest activation energy (27.7 kJ/mol) was found at the Cu^4^–Cu^4^ bridge site for H–O bond formation, while 40.4 kJ/mol was the lowest for H–O and H–Cu bond formation. This suggests that H_2_ dissociation favored H–O bond formation (Fig. 6d). In contrast, the diffusion barrier for H migration from H–Cu to H–O was much lower (13.5 kJ/mol) (Fig. 6e), suggesting that H atoms diffuse easily on the CuO surface. Moreover, the energy barrier for H diffusion from the surface to subsurface was 337.4 kJ/mol (obtained from the difference between the total energy of TS (240.8 kJ/mol) and that of the H–O/H–O absorbed surface state (–96.6 kJ/mol, Fig. 6e), indicating that subsurface diffusion is rate-limiting.

The effects of H_2_ molecule surface coverage on H_2_ adsorption structure on CuO (111) surface were examined by setting various surface coverage rates of H_2_ molecules on CuO(111) surface. The results revealed that H_2_ molecules moved away from the surface at all surface coverage rates (0.0625–1 ML, Fig. S14). The effect of H-atom surface coverage on H adsorption structure on CuO(111) was also examined by setting various surface coverage rates of H atoms on the CuO(111) surface. The results revealed that H atoms most readily adsorb onto O^3^ atoms, followed by Cu^3^ atoms, with the increase of H coverage (Fig. S15). The adsorption energy of an H atom on the CuO (111) surface decreased with the increase of H coverage up to 0.5 ML (Fig. S16). Furthermore, when the H surface coverage exceeds 0.5 ML, additional H atoms can no longer adsorb onto the surface. They detach from the surface and form hydrogen molecules (Figs. S15 c.4 and d.4).

The hydrogen atom solid solubility limit in CuO was evaluated by calculating the solution energies when inserting H atoms sequentially up to 9 atoms into interstitial octahedral sites in a larger slab model comprising 256 atoms (Figs. S17 and S18). The H solubility at ΔG_sol = 0 was determined as 0.01377 at% by extrapolation, indicating an extremely low H solubility in CuO at 298 K and 1.27 MPa (Fig. S19). These DFT results align with the ToF-SIMS findings (Fig. 5d), revealing that the CuO layer effectively blocks hydrogen permeation.

We successfully synthesized an ErCo_2_ magnetic refrigeration material with excellent HPB performance at 1.27 MPa and ~296 K through electroless Cu plating followed by oxidation. The CuO layer effectively blocked hydrogen permeation because of its high activation energy for H diffusion ( ~ 337 kJ/mol). This low-cost and efficient approach can be applied to other materials that require HPB properties near room temperature.

## Methods

### Fabrication of ErCo_2_ spherical particles

A master alloy ingot of ErCo_2_ was prepared using high-frequency vacuum melting equipment with pure raw materials of Er (99.9%, Nippon Yttrium Co., Ltd.) and Co (99.9%, Sumitomo Metal Mining Co., Ltd.). ErCo_2_ spherical particles were then fabricated from the ingot using an EIGA process^33–35^. The ingot, serving as an electrode rod, was inductively melted without a crucible, enabling the production of high-purity spherical particles. After atomization, the particles were sieved, and those with diameters of 212–355 μm were collected and homogenized at 1123 K for seven days in an Ar gas atmosphere for subsequent experiments.

### Oxidation treatments and electroless Cu plating

Oxidation treatments were conducted on both the as-homogenized and Cu-plated particles using a box-type electric furnace in air. The oxidation conditions were varied within the ranges of 473 to 773 K and 0.1 to 0.5 h. Oxidation at 773 K for 0.5 h was more effective for forming the CuO layer. The samples were heated to 773 K at a rate of ~15 K/min, held at this temperature for 0.5 h, and then slowly cooled to room temperature in the furnace.

Electroless Cu plating was performed using a plating solution with the composition listed in Table S3, at a pH of 12–12.5. The plating was conducted at 248 K for 1.5 h with magnetic stirring. Prior to plating, the ErCo₂ particle surfaces were cleaned by immersion in 4 vol% HCl aqueous solution at 298 K for 20 s, followed by rinsing with purified water. This pre-cleaning step was essential for ensuring uniform Cu deposition, as some particles failed to be plated without it (Fig. S20). SEM and EDS analyses revealed that a very thin Er–Co oxide layer existed on the surface of as-homogenized ErCo_2_ particles, formed during the homogenization heat treatment process or subsequent storage at room temperature in air. This oxide layer is believed to affect the copper plating process and needs to be removed by pre-cleaning. The short pre-cleaning time of 20 s was used to avoid unnecessary sample elution (Table S4).

### Evaluation test of HPB performance

The HPB performance of ErCo₂ particles, both before and after Cu plating, was evaluated using a Sieverts-type instrument (Suzuki Syokan Co., Ltd.). A schematic of the hydrogen exposure system and detailed experimental setup are provided in the Supporting Information (Fig. S1). For each measurement, 0.5 g of particles was used. Hydrogen gas was introduced at an initial pressure of ~1.27 MPa after vacuuming with a rotary pump for at least 15 min. The average relative error of the pressure gauge (PG-50KU-F, KYOWA Electronic Instruments Co., Ltd., Japan) is ±0.5%. The calibration sheet of the gauge shows its rate output is 1997 μV/V, and the calibration constant is 0.00245 MPa/ 1μV/V. The pressure change in the sealed cell was monitored for up to seven days at 293–298 K to assess the HPB properties of the particles. We performed the D_2_ exposure experiment at the same temperature and pressure as that for the hydrogen exposure experiments, and the duration was also very close, i.e., 137.5 h for oxidized particles and 139 h for Cu-plated/oxidized particles.

The amount of hydrogen adsorbed by the ErCo₂ sample (Δ*n*, mol) at each time point was calculated using Eq. (1), derived from the ideal gas law:

1 $$\triangle n=(\frac{{P}_{0}V}{R{T}_{0}}-\frac{{P}_{i}V}{R{T}_{i}})$$

where *P*_0_ and *T*_0_ are the initial hydrogen pressure (Pa) and temperature (K) in the cell, respectively, while *P*_i_ and *T*_i_ represent the pressure and temperature at the i-th measurement. *V* is the total volume of the cell, reservoir, and gas pipeline (m³), and *R* is the molar gas constant (8.314462 J K⁻¹ mol⁻¹).

The hydrogen absorption capacity of the ErCo₂ particles was calculated as:

2 $$	 {{\rm{Absorbed}}}\; {{\rm{hydrogen}}}\; ({{\rm{mass}}}\%) \\ 	={{\rm{weight}}}\; {{\rm{of}}}\; {{\rm{absorbed}}}\; {{\rm{hydrogen}}}/{{\rm{weight}}}\; {{\rm{of}}}\;{{{\rm{ErCo}}}}_{2}\times 100$$

### Characterization

The morphology and composition of ErCo_2_ particles, before and after Cu plating and oxidation treatments, were analyzed using SEM (JEOL, JSM-7000F, JSM-6500F) and TEM (JEOL, JEM-ARM200F) coupled with X-ray energy-dispersive spectroscopy. Cross-sectional SEM samples were prepared by mechanically polishing the particles affixed to a conductive adhesive. TEM samples were prepared from SEM cross-sections using a focused ion beam technique, with details provided in the Supporting Information (Fig. S21). The crystalline structures of ErCo₂ particles at various stages of treatment were analyzed via SXRD (SPring-8, BL02B2). The wavelength of the incident beam (λ) was 0.775980 Å.

To assess the effects of Cu coating and oxidation on the magnetic properties of ErCo₂ particles, temperature-dependent magnetization (M–T) measurements were conducted using a Quantum Design SQUID magnetometer. Measurements were taken from 2 to 60 K under applied magnetic fields ranging from 0.001 to 5 T. All the *M*(*T*) measurements were performed under field-cooling processes. The temperature sweep rate was 0.6 K per min; the mass of the samples was approximately 3–4 mg. Demagnetization corrections were not performed in this study because it is difficult to evaluate the exact demagnetization factors for a group of particles. The demagnetization effect does not significantly affect the evaluation of Δ*S*_M_ for large magnetic fields such as 5 T^44^. The magnetic entropy change (∆*S*_*M*_) was calculated using the following equation^45^.

3 $$-\triangle {S}_{M}=-{\mu }_{0}{\int }_{0}^{H}{\left(\frac{\partial M}{\partial T}\right)}_{H}{dH}$$

where *μ*_0_ is the permeability of vacuum, *H* is the external magnetic field, *T* is the temperature, and *M* is the magnetization. The measurement error of Δ*S*_M_ is mainly from the weight error of the particle samples used in the measurements, which is estimated to be approximately 1–2%.

ToF-SIMS analysis of the particles after the deuterium exposure test was performed using a time-of-flight secondary ion mass spectrometer (PHI, TRIFT V nanoTOF). A Ga emitter with 30 kV was used to sputter the particle surface. Deuterium (an isotope of hydrogen) was selected because it exhibits diffusion behavior similar to that of hydrogen^46,47^ and has a detection limit 1–2 orders of magnitude lower than that of hydrogen in ToF-SIMS^48,49^. D_2_ also eliminates interference from trace hydrogen originally present in the sample and chamber. The D concentration was derived from the ^2^H signal by subtracting the background signal of ^1^H/100 intensity, which was confirmed to appear at the ^2^H position in all measurements.

### DFT calculation

The adsorption energies and the dissociation energy of molecular hydrogen on the CuO (111) surface were calculated using the PHASE0 first-principles program package based on DFT (https://azuma.nims.go.jp/)^50^. The exchange-correlation energy was described using the generalized gradient approximation (GGA)^51–53^. A slab model comprising 128 atoms in four layers (4 × 8 atoms per layer) was used with a vacuum layer of 15 Å to prevent interactions between periodic images. The Brillouin zone was sampled using a 2 × 2 × 1 k-point mesh, with cut-off energies of 450 eV for the wave function and 4050 eV for the charge density. The state determination was performed using a convergence criterion of 5 × 10^–6^ eV for total energy and a force criterion of 0.01 eV/Å for force action.

The zero-point energy (ZPE) correction was performed by calculating vibration modes using the linear response function of PHASE0 after optimizing the slab model and each adsorption state, and the ZPE energy was calculated as follows:

4 $${{\rm{ZPE}}}=\frac{1}{2}{\sum }_{i=1}^{n}\left(h{\gamma }_{i}\right)$$

where ${\gamma }_{i}$ is the frequency of each actual value vibration mode, *n* is the total numbers of actual value vibration mode, and *h* is Planck constant (4.135667696 × 10^-15 ^eV·s). By this method, the ZPE value of the free H_2_ molecule was calculated to be 0.273436 eV, which agrees well with the literature value^54^.

The adsorption energy of a single molecule on the surface (*E*_*abs*_) was calculated as follows:

5 $${E}_{{ads}}=({E}_{{slab}}+{{{\rm{ZPE}}}}_{{slab}})+({E}_{{H}_{2}}+{{{\rm{ZPE}}}}_{{H}_{2}})-({E}_{{H}_{2}/{slab}}+{{{\rm{ZPE}}}}_{{H}_{2}/{{\rm{slab}}}})$$

where ${E}_{{slab}}$ is the total energy of the slab, ${E}_{{H}_{2}}$ is the total energy of an isolated hydrogen molecule, ${E}_{{H}_{2}/{slab}}$ is the energy of the slab with adsorbed hydrogen, ${{{\rm{ZPE}}}}_{{slab}}$ is the ZPE of the slab, ${{{\rm{ZPE}}}}_{{H}_{2}}$ is the ZPE of a free H_2_ molecule, and ${{{\rm{ZPE}}}}_{{H}_{2}/{slab}}$ is the ZPE of the slab with adsorbed hydrogen. A larger *E*_*ads*_ indicates stronger hydrogen adsorption.

The activation energies for H_2_ dissociation and atom diffusion on CuO(111) surface were determined using the climbing image nudged elastic band method^55^. The transition states were determined by interpolating 6 images between the initial and final states. The transition state determination was performed using a convergence criterion of 5 × 10^–6^ eV for total energy and a force criterion of 0.01 eV/Å for force action. The k-point mesh was 2×2×1. The cut-off energies were 450 eV for the wave function and 4050 eV for the charge density.

The effects of H_2_ surface coverage on H_2_ adsorption structure on CuO (111) surface were examined by setting various surface coverage rates of H_2_ molecules on CuO(111) surface (0.0625–1 ML) (Fig. S14). In the initial state, the H_2_ molecules were placed at positions approximately 1.5 Å above each outermost O or Cu atom, with their orientation parallel to the *x*-axis. The effect of H surface coverage on H adsorption structure on CuO (111) surface was examined by setting various surface coverage rates of H atoms on CuO(111) surface (0.0625–1ML) (Fig. S15). In the initial state, the H atoms were placed at positions approximately 1.5 Å above each outermost O or Cu atom. The slab model and calculation conditions for these surface coverage effects were the same as those for the single H_2_ molecule on the CuO(111) surface.

The hydrogen atom solid solubility limit in CuO was evaluated by calculating the solution energies as follows, when inserting H atoms sequentially up to 9 atoms into interstitial octahedral sites in a larger slab model comprising 256 atoms (Fig. S17). Structural optimization of the slab model was first performed with all Cu and O atoms movable. Subsequently, the total energies of each state with solid solution H atoms were calculated by fixing the Cu and O atoms in the optimized model, and only the H atoms were movable. The Brillouin zone was sampled using a 1×2×3 k-point mesh, with cut-off energies of 450 eV for the wave function and 1800 eV for the charge density. The state determination was performed using a convergence criterion of 1 × 10^–5^ eV for total energy and a force criterion of 0.01 eV/Å for force action. The solution energy for solid solution of H atoms into CuO (${\triangle G}_{{sol}}$) was calculated as follows:

6 $${\triangle G}_{{sol}}=	 ({E}_{({CuO}+{nH})}+{{{\rm{ZPE}}}}_{({CuO}+{nH})})-({E}_{{CuO}}+{{{\rm{ZPE}}}}_{{CuO}})\\ 	 -\frac{n}{2}({E}_{{H}_{2}}+{{{\rm{ZPE}}}}_{{H}_{2}}+{\mu }_{{H}_{2}}(T,p))$$

where *n* is the numbers of H atom (*n* = 1 ~ 9), ${E}_{({CuO}+{nH})}$ and ${{{\rm{ZPE}}}}_{({CuO}+{nH})}$ are the total energy and ZPE of CuO with solubilized H atoms, ${E}_{{CuO}}$ and ${{{\rm{ZPE}}}}_{{CuO}}$ are the total energy and ZPE of CuO, ${E}_{{H}_{2}}$ and ${{{\rm{ZPE}}}}_{{H}_{2}}$ are the total energy and ZPE of free H_2_ molecule, and ${\mu }_{{H}_{2}}(T,p)$ is the chemical potential of gaseous H_2_ contributed by temperature (*T*) and pressure (*p*) as follows.

7 $${\mu }_{{H}_{2}}\left(T,p\right)={\mu }_{{H}_{2}}(T,{p}^{\circ} )+{k}_{B}T{\mathrm{ln}}(\frac{p}{p^\circ })$$

The H concentration $({c}_{H})$ was estimated as follows:

8 $${c}_{H}\approx \exp (-\frac{{\triangle G}_{{sol}}}{{k}_{B}T})$$

where ${k}_{B}$ is Boltzmann’s constant (1.380649×10^–23^ J/K), *T* is absolute temperature (K), *p*° is the reference pressure, and *p* is the actual pressure. Moreover, ${\mu }_{{H}_{2}}(T,p^\circ )$ is the chemical potential of H_2_ at the reference pressure (1 atm), which was obtained from the NIST standard reference database (10.18434/T4D303).

## Supplementary information

Supplementary Information

Transparent Peer Review file

## Source data

Source Data

## Data Availability

All relevant data are included in the article and its supplementary information files. Source data are provided with this paper. Further data are available from the corresponding author upon request. Source data are provided with this paper.
