# Supplementary material for: Enhancing hydrogen permeation barrier performance of ErCo2 magnetic refrigeration material via surface oxide layer formation
Source: Nat Commun. 2026 Apr 7;17:4952. doi: 10.1038/s41467-026-71547-0 (PMC13233819; doi:10.1038/s41467-026-71547-0)
Supplement: Supplementary file 1 — Supplementary Information [file 41467_2026_71547_MOESM1_ESM.pdf]

Supplementary information for

Enhancing hydrogen permeation barrier performance of ErCo2 magnetic refrigeration material via surface oxide layer formation

Ya Xu[1]*, Keiji Oyoshi[1+], Haruka Yoshikawa[1,2++], Hiroyuki Takeya[1], Hiroshi Amekura[1], Takafumi D. Yamamoto[3+++], Yoshitaka Matsushita[4], Alexei A. Belik[3], Miyoko Tanaka[4], Akiko T. Saito[5], Koji Kamiya[1], Yoshihiko Takeda[1]

[1]Research Center for Energy and Environmental Materials (GREEN), National Institute for Materials Science (NIMS), 3-13 Sakura, Tsukuba, Ibaraki, 305-0003, Japan
[2]Faculty of Advanced Engineering, Tokyo University of Science, 6-3-1 Niijyuku, Katsushika, Tokyo 125-8585, Japan
[3]Research Center for Materials Nanoarchitectonics (MANA), National Institute for Materials Science (NIMS), Namiki 1-1, Tsukuba, Ibaraki, 305-0044, Japan
[4]Research Network and Facility Services Division, National Institute for Materials Science (NIMS), Sengen 1-2-1, Tsukuba, Ibaraki, 305-0047, Japan
[5]Research Center for Magnetic and Spintronic Materials, National Institute for Materials Science (NIMS), 3-13 Sakura, Tsukuba, Ibaraki, 305-0003, Japan

*Corresponding author, xu.ya@nims.go.jp

[+] Present affiliation: Research Network and Facility Services Division, National Institute for Materials Science (NIMS), Tsukuba, Japan
[++] Present affiliation: Mitsubishi Kakoki Kaisha, Ltd., Kanagawa, Japan
[+++] Present affiliation: Department of Materials Science and Technology, Tokyo University of Science, Tokyo, 125-8585, Japan

This supplementary information file includes:
Figures S1-21
Tables S1-4

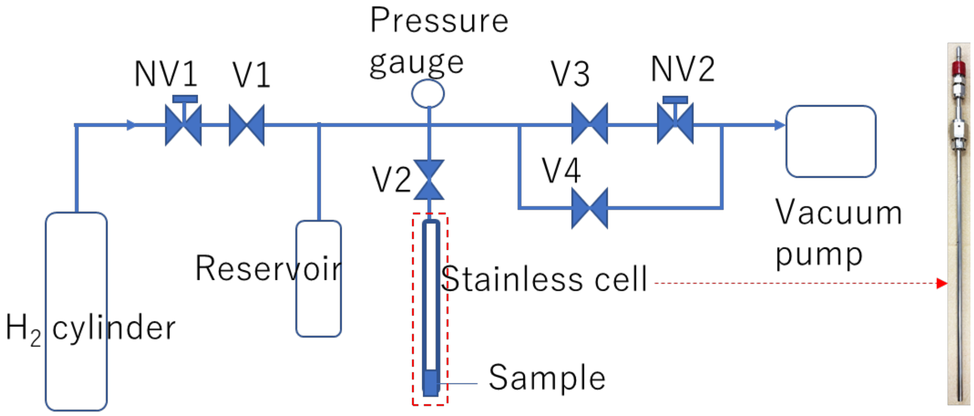

Fig. S1. Schematic of the Sieverts-type hydrogen exposure experiment system. NV1 and NV2: Needle valve; V1-4: valve.

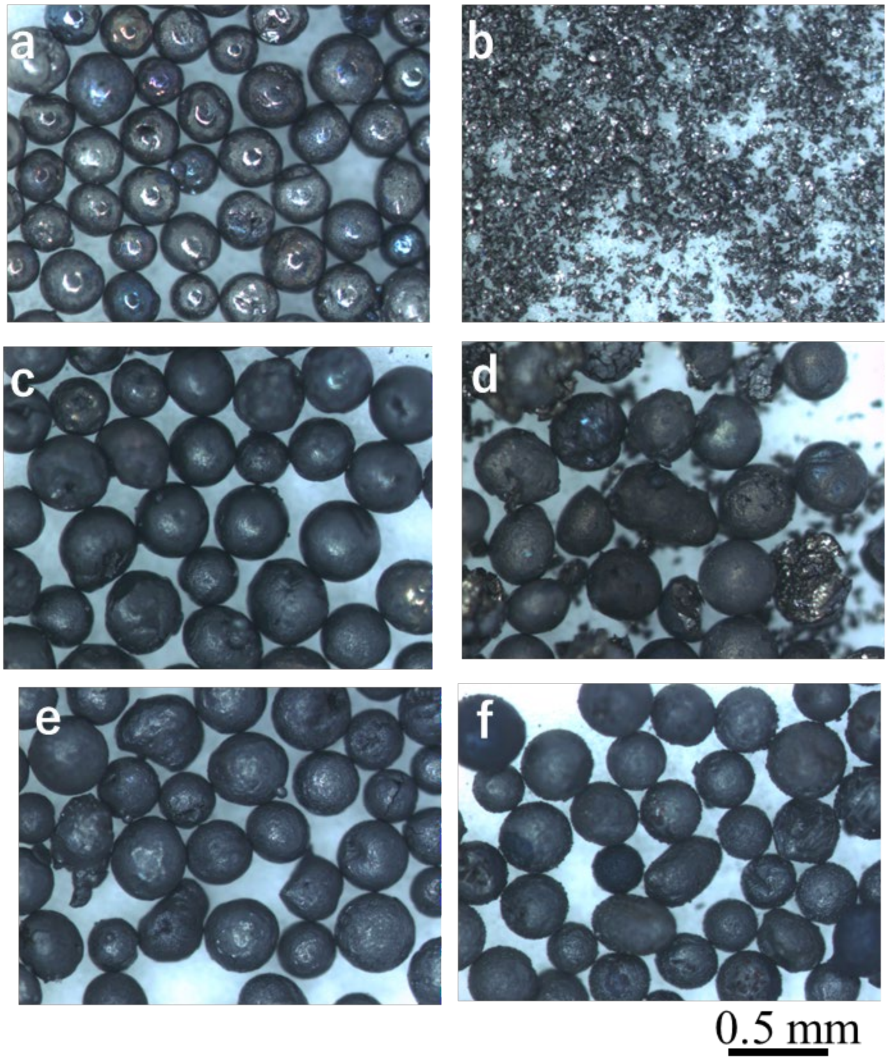

Fig. S2. Observation of shape change of $ErCo_2$ particles before and after hydrogen exposure experiment using stereo microscope. **a**. as-homogenized particles before hydrogen exposure; **b**. As-homogenized particles after hydrogen exposure. **c**. oxidized particles. **d**. oxidized particles after hydrogen exposure. **e**. Cu-plated/oxidized particles. **f**. Cu-plated/oxidized particles after hydrogen exposure.

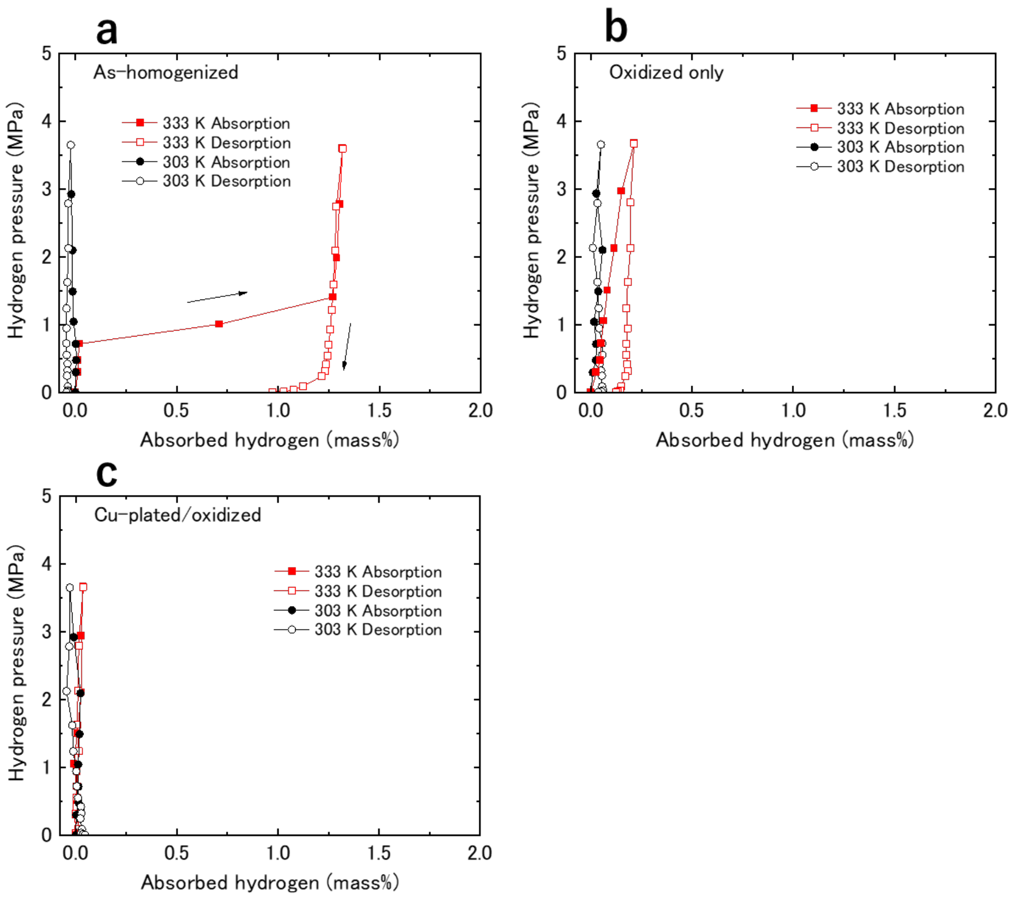

Fig. S3. Pressure-composition-temperature (PCT) measurement results of $ErCo_2$ particles. **a**. the as-homogenized samples; **b**. the samples after oxidized at 773 K for 0.5 h in air (oxidized only); **c**. the samples after Cu-plated at 348 K for 0.17 h followed by the oxidation at 773 K for 0.5 h in air (Cu-plated/oxidized).

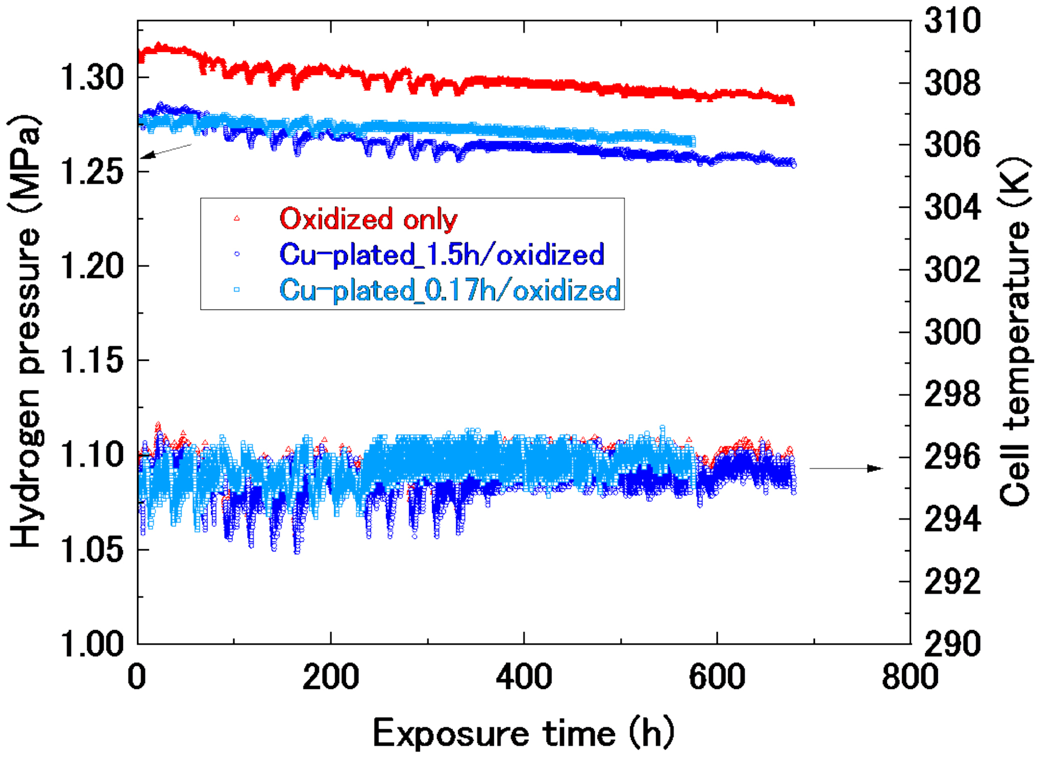

Fig. S4. Hydrogen pressure and cell temperature changes during the long-term hydrogen exposure test for the oxidized only, Cu-plated_0.17h/oxidized, and Cu-plated_1.5h/oxidized $ErCo_2$ particles. Numerous small pressure fluctuations during the measurement process are due to variations in cell temperature (room temperature).

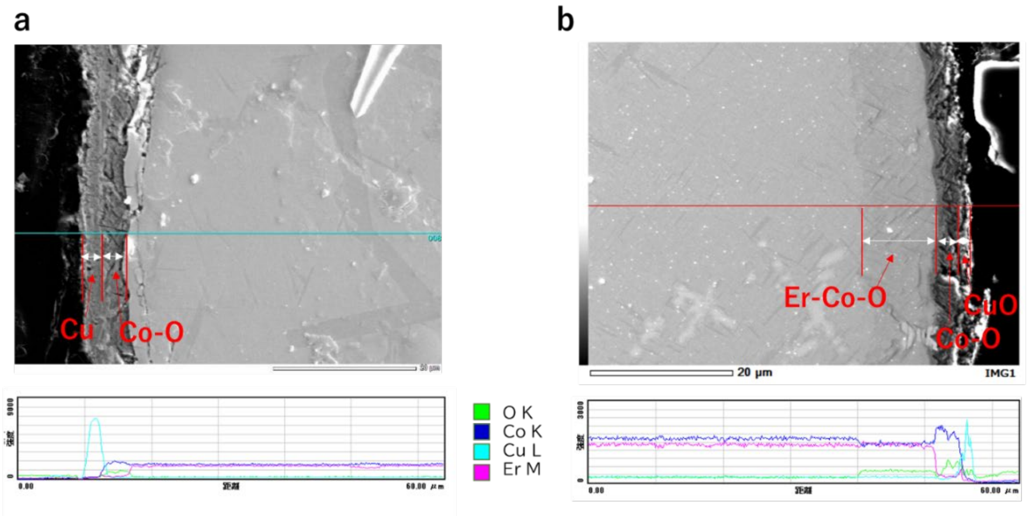

Fig. S5. SEM images and EDS line analysis results of the cross-section of the particle with Cu plating for 0.17 h at 348 K (Cu-plated_0.17h $ErCo_2$) (**a**) and after subsequent oxidation treatment (Cu-plated_0.17h/oxidized $ErCo_2$) (**b**).

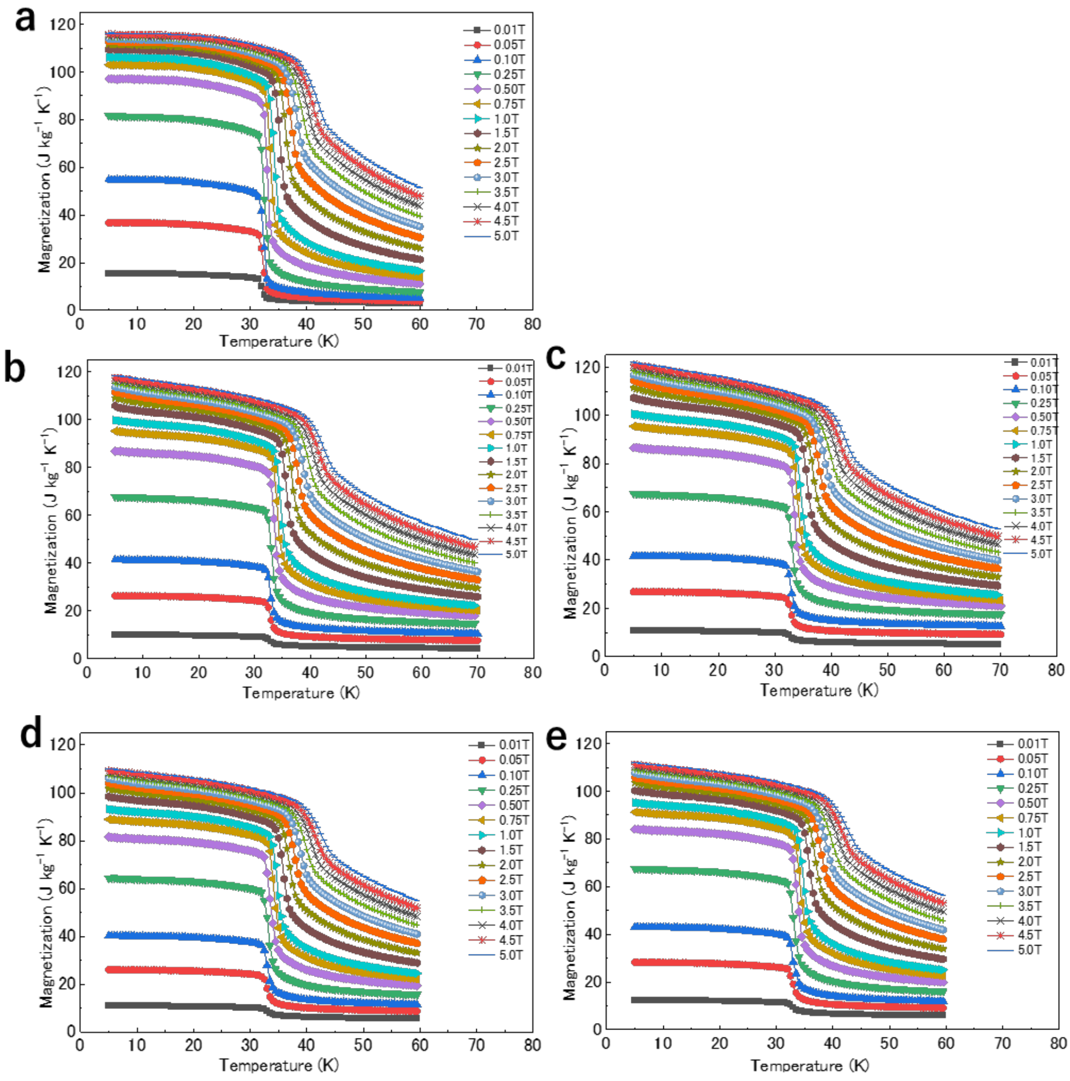

Fig. S6. M-T curves under varying field up to 5 T for the as-homogenized $ErCo_2$ (**a**), oxidized $ErCo_2$ before H_2 exposure (**b**) and after H_2 exposure (**c**), and Cu-plated/oxidized $ErCo_2$ H_2 exposure (**d**) and after H_2 exposure (**e**).

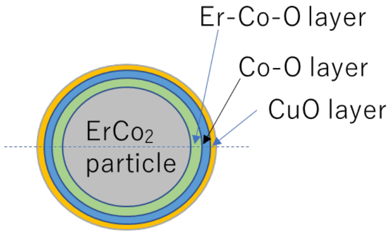

	Thickness (μm)	Volume (cm^3)	Density (g/cm^3)	Mass (g)
$ErCo_2$ Particle	251.6(diameter)	8.33509E-06	10.35	8.62682E-05
Er-Co-O layer	10	2.14989E-06	8	1.71991E-05
Co-O layer	7	1.7064E-06	6.5	1.10916E-05
CuO layer	4	1.06805E-06	6.31	6.7394E-06

Fig. S7. Schematic cross-section of a Cu-plated/oxidized $ErCo_2$ particle, and estimated weight of each layer. The thickness is the average value obtained from the SEM observation data by measuring 3-5 particles. (For the mass calculation, the density of Er-Co-O layer was assumed to be 8 g/cm^3 which is less than that of $ErCo_2$ (10.35 g/cm^3) but higher than that of CoO (6.5 g/cm^3).)

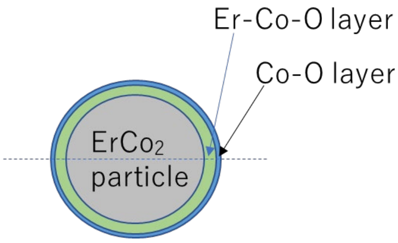

	Thickness (μm)	Volume (cm^3)	Density (g/cm^3)	Mass (g)
$ErCo_2$ Particle	267.6(diameter)	1.00285E-05	10.35	1.03795E-04
Er-Co-O layer	13	3.21632E-06	8	2.57305E-05
Co-O layer	2	5.48751E-07	6.5	3.56688E-06

Fig. S8. Schematic cross-section of an oxidized $ErCo_2$ particle, and estimated weight of each layer. The thickness is the average value obtained from the SEM observation data by measuring 3-5 particles. (For the mass calculation, the density of Er-Co-O layer was assumed to be 8 g/cm^3 which is less than that of $ErCo_2$ (10.35 g/cm^3) but higher than that of CoO (6.5 g/cm^3).)

a

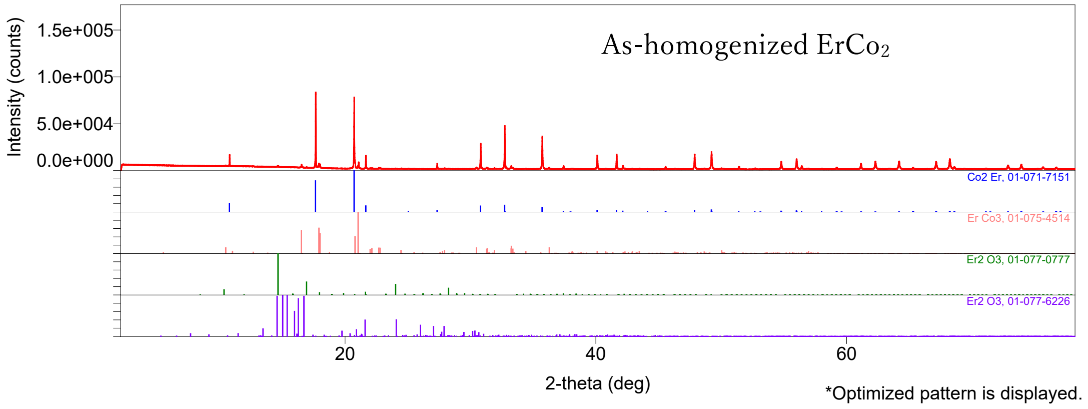

b

**Phase**	**Space group**	**DB card numbers** ICDD(PDF-2)	**Lattice parameters**				**Crystallite size** (Å)	**Content** (mass%)
			a (Å)	b (Å)	c (Å)	β(deg.)		
$ErCo_2$	227 : Fd-3m, choice-2	01-071-7151	7.1548223 (10)	= a	= a	-	1455(78)	93.92(16)
$ErCo_3$	166 : R-3m, hexagonal	01-075-4514	4.9832(7)	= a	24.239(5)	-	469(138)	5.44(16)
Er_2O_3	206 : Ia-3	01-077-0777	10.544851 (18)	= a	= a	-	271(38)	0.45(2)
Er_2O_3	12 : C12/m1, unique-b	01-077-6226	13.82(5)	3.444(11)	8.56(3)	100.36(16)	163(23)	0.19(4)

Fig. S9. Qualitative analysis results of synchrotron X-ray diffraction (SXRD) measurement of as-homogenized $ErCo_2$. **a**. SXRD profile and peak profiles of identified phases. **b**. Crystal structure, lattice parameters, crystallite size, and content of identified phases.

Measurement conditions:

X-ray: Synchrotron (SPring-8 BL02B2, λ=0.775980 Å)

Scan mode: STEP; Duration time: 300 sec; Step width: 0.0060 deg.; Scan axis: 2θ-θ; Scan range: 2.08-78.2 deg.

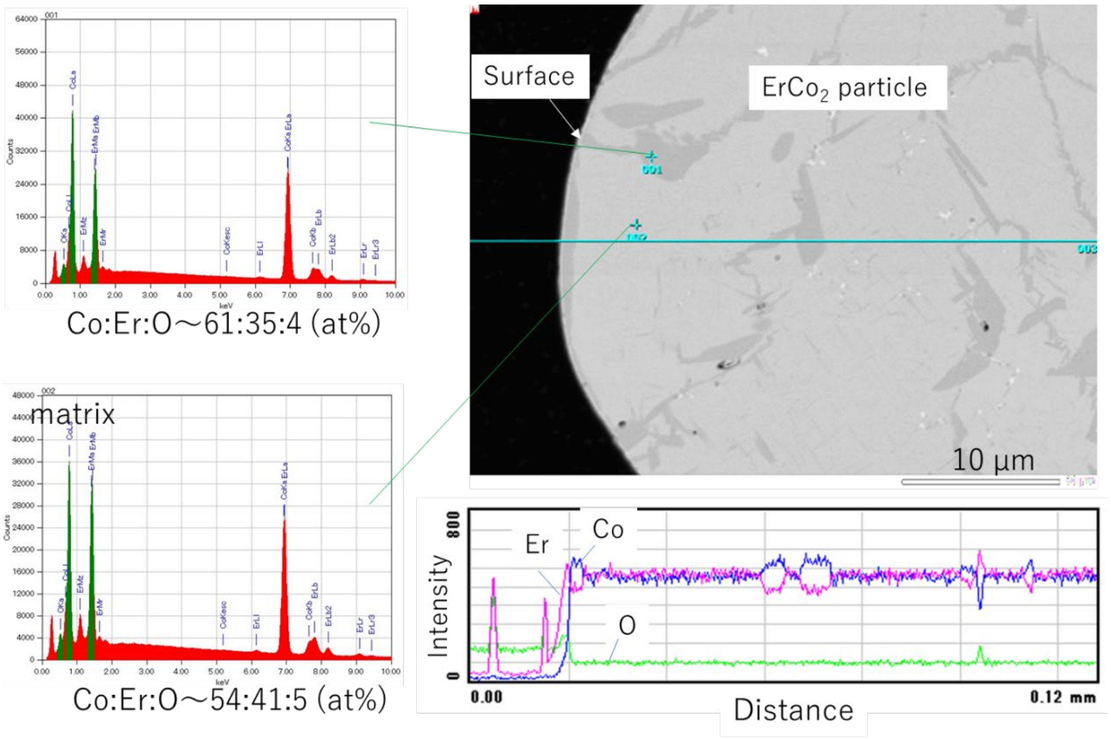

Fig. S10. Secondary electron (SE) image of cross-section of as-homogenized $ErCo_2$ particle and corresponding line Energy-dispersive X-ray spectroscopy (EDS) analysis results. Spot EDS analysis results at area with dark contrast and at substrate were also shown.

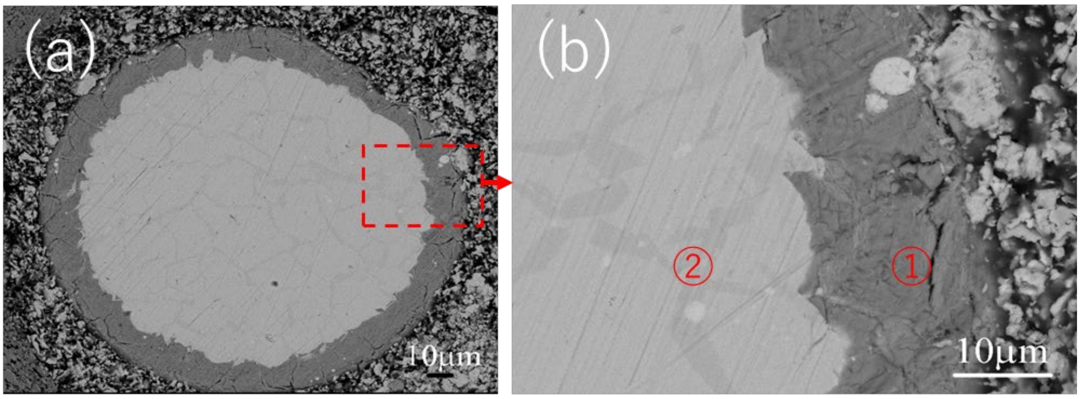

(c)

Position	O (at%)	Co (at%)	Er (at%)
①	42	23	35
②	8	32	60

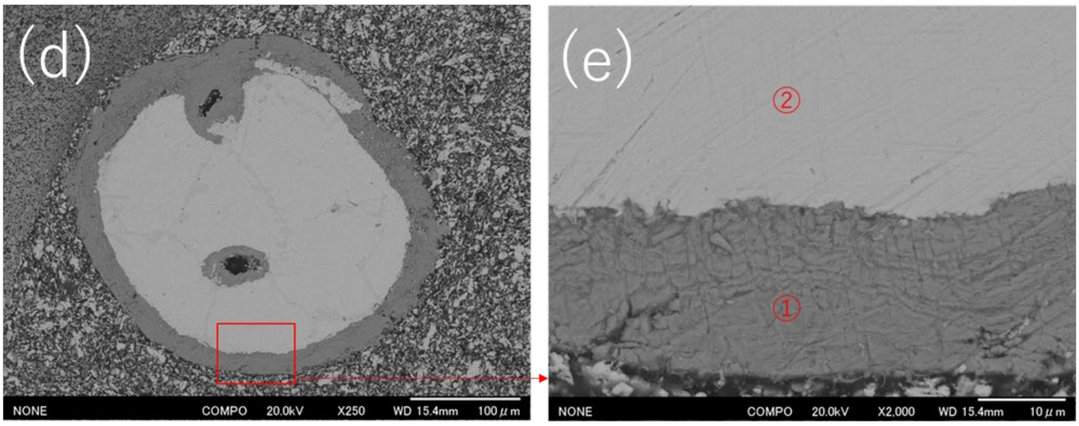

(f)

Position	O (at%)	Co (at%)	Er (at%)
①	39	36	25
②	9	33	58

Fig. S11. **a**. Back scattered electron (BSE) image of cross-section of a $ErCo_2$ particle after acid treatment in 4% HCl solution for 20 seconds; **b**. BSE image of the surface layer region; **c**. EDS spot analysis results of the positions marked in **b; d**. BSE image of cross-section of a $ErCo_2$ particle after acid treatment in 4% HCl solution for 600 seconds; **e**. BSE image of the surface layer region; **f**. EDS spot analysis results of the positions marked in **e**.

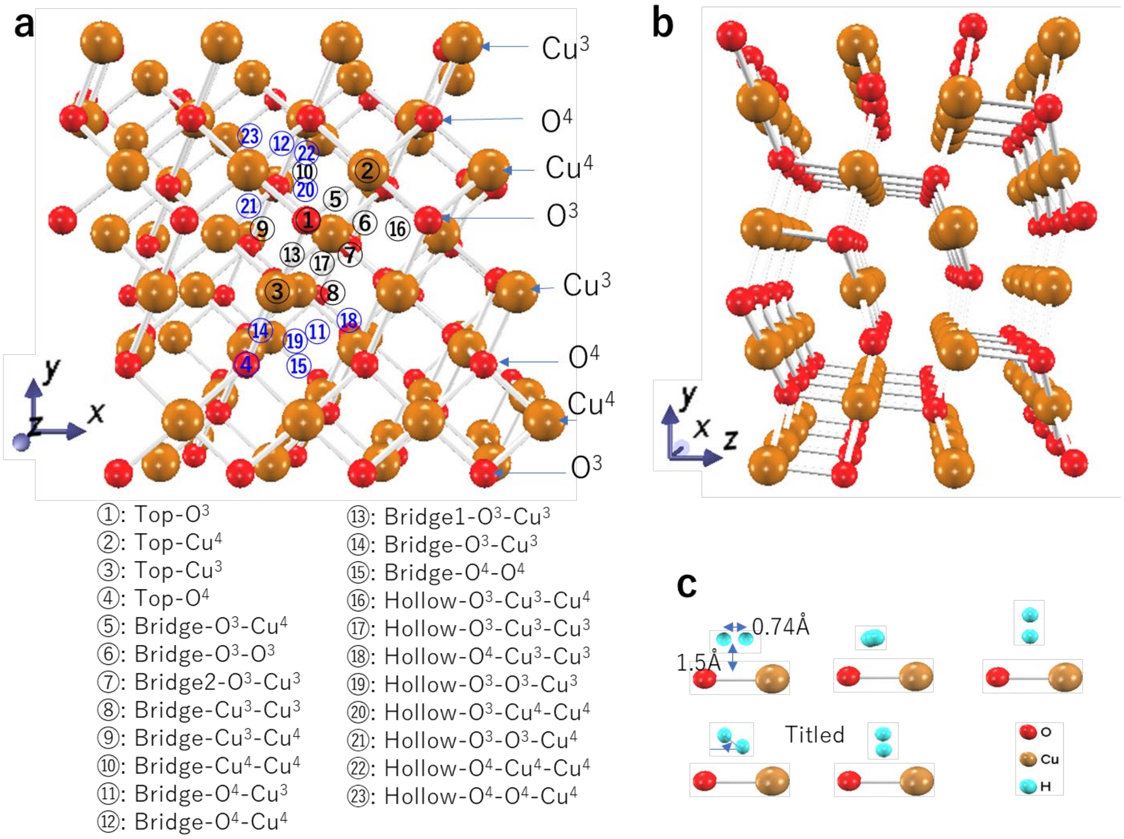

Fig. S12. Various adsorption sites on the slab model of a p(4×2) CuO(111) surface. **a**. top-view. **b**. left-view. **c**. five orientations of the H_2 molecules set in the initial state: one perpendicular to the surface, two parallel to the surface: at the bridge site they were parallel or perpendicular to the bridge; at the hollow site, they were parallel to the x- or y-axis, and two tilted approximately 45°toward to z-axis from the two orientations parallel to the surface. The interatomic distance of H_2 molecules was set to 0.74 Å and the distance between the hydrogen molecules and the CuO(111) surface to about 1.5 Å in the initial state.

O^3: three-coordinated O atom; O^4: four-coordinated O atom; Cu^3: three-coordinated Cu atom; Cu^4: four-coordinated Cu atom.

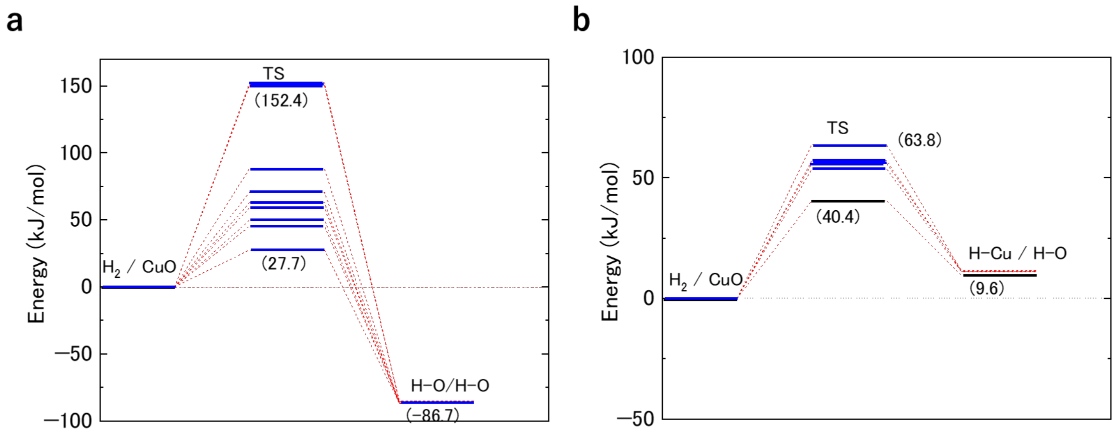

Fig. S13. Potential energy profiles for the dissociation of H_2 started from different adsorption sites (initial states) to two kinds of final states: **a**. two H-O bonds. **b**. one H-Cu and one H-O bond.

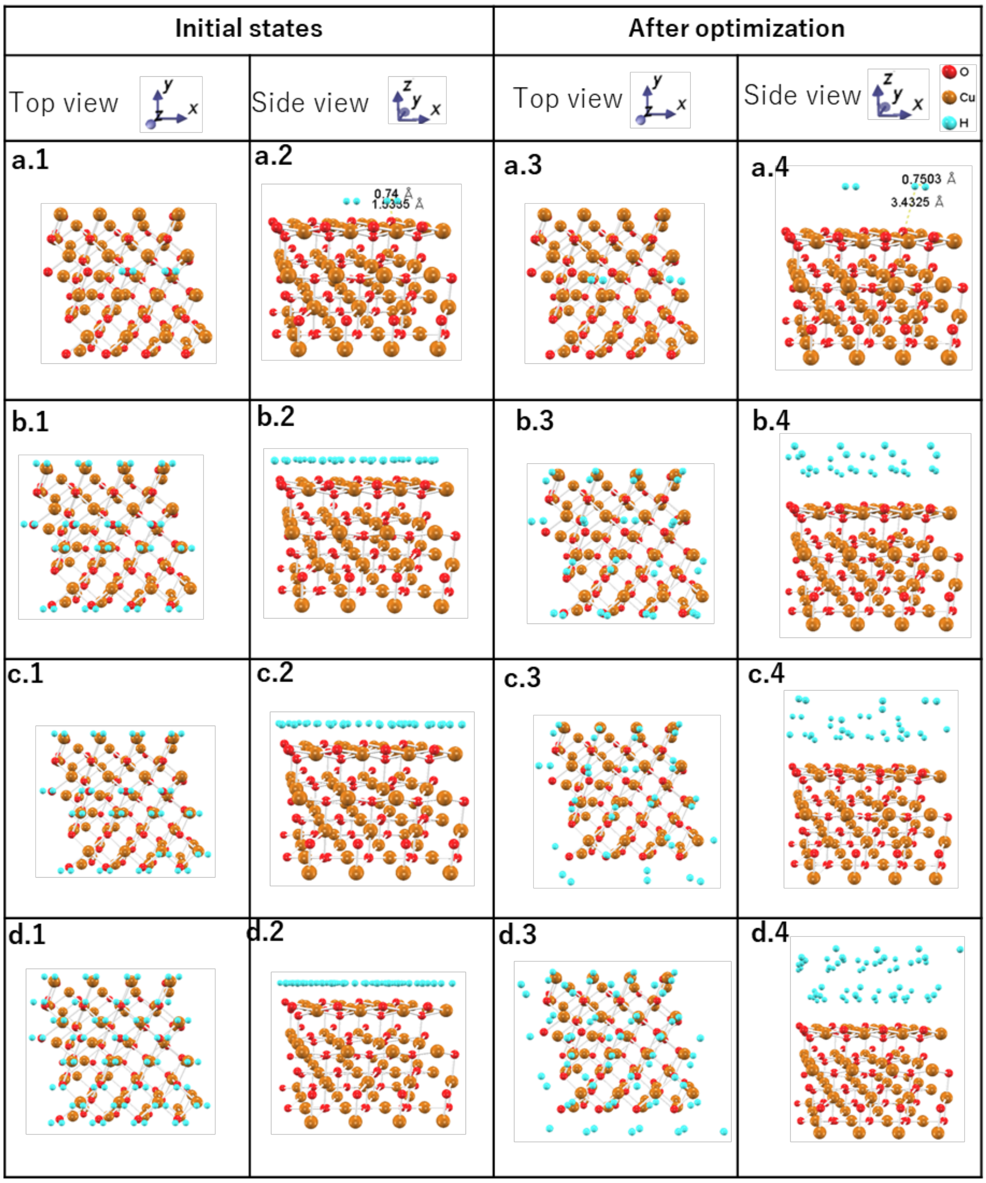

Fig. S14. Effect of H_2 surface coverage on H_2 adsorption structure on CuO (111) surface. **a.1~4**: 0.0625 ML, **a.1** (top view) and **a.2** (side view): initial state, **a.3** (top view) and **a.4** (side view): after optimization; **b.1~4**: 0.5 ML, **b.1** (top view) and **b.2** (side view): initial state; **b.3** (top view) and **b.4** (side view): after optimization; **c.1~4**: 0.5625 ML, **c.1** (top view) and **c.2** (side view): initial state; **c.3** (top view) and **c.4** (side view): after optimization; **d.1~4**: 1.0 ML, **d.1** (top view) and **d.2** (side view): initial state; **d.3** (top view) and **d.4** (side view): after optimization. The initial distance between H_2 and CuO(111) surface was set as approximately 1.5 Å, and the distance between H atoms in H_2 molecule was approximately 0.74 Å. After optimization, the distance between H_2 and CuO surface was larger than 2.8 Å, and the distance between H atoms in H_2 molecule was approximately 0.75 Å.

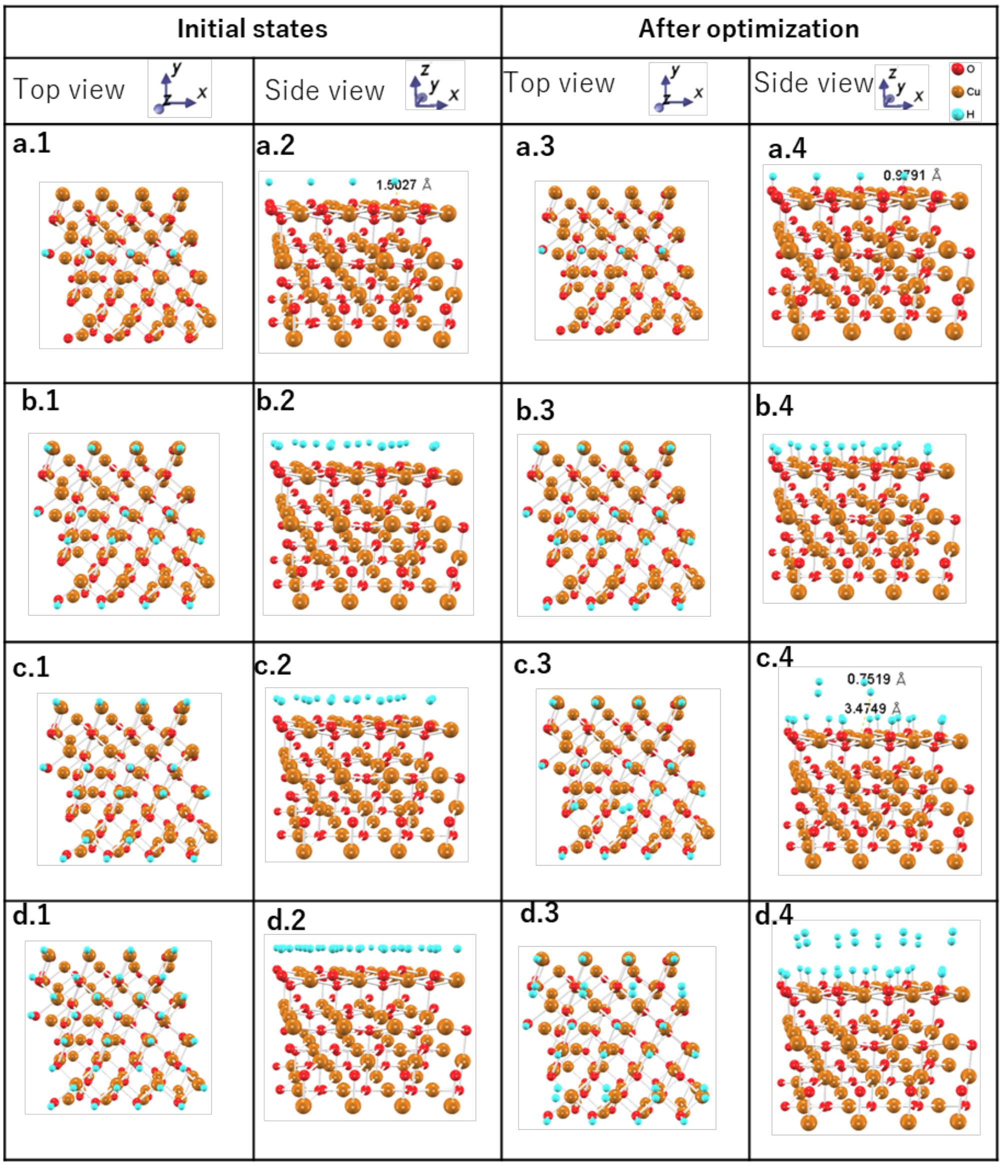

Fig. S15. Effect of H surface coverage on H adsorption on CuO (111) surface. **a.1~4**: 0.0625 ML, **a.1** (top view) and **a.2** (side view): initial state, **a.3** (top view) and **a.4** (side view): after optimization; **b.1~4**: 0.5 ML, **b.1** (top view) and **b.2** (side view): initial state; **b.3** (top view) and **b.4** (side view): after optimization; **c.1~4**: 0.5625 ML, **c.1** (top view) and **c.2** (side view): initial state; **c.3** (top view) and **c.4** (side view): after optimization; **d.1~4**: 1.0 ML, **d.1** (top view) and **d.2** (side view): initial state; **d.3** (top view) and **d.4** (side view): after optimization. The initial distance between H and CuO surface was set as approximately 1.5 Å, and the H atoms were preferentially set on the top of O^3 and Cu^3 atoms, and then on the top of O^4 and Cu^4 atoms. After optimization, the distance between the H and the O^3 atom was approximately 0.98 Å, and the distance between the H atom and the Cu^3 atom was approximately 1.5 Å.

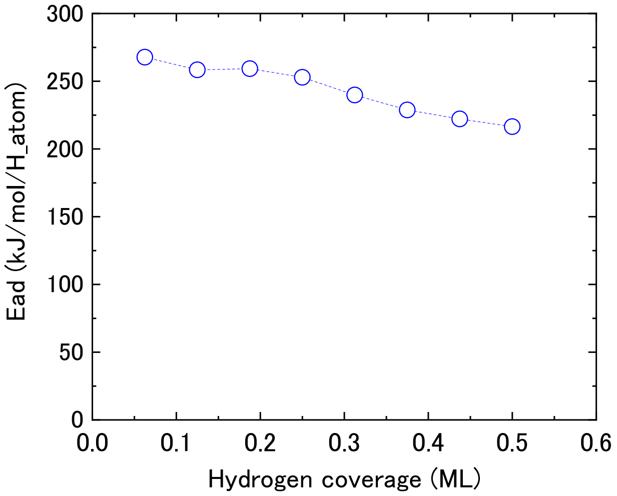

Fig. S16. Adsorption energy of a H atom on the CuO (111) surface plotted as a function of H coverage (ML).

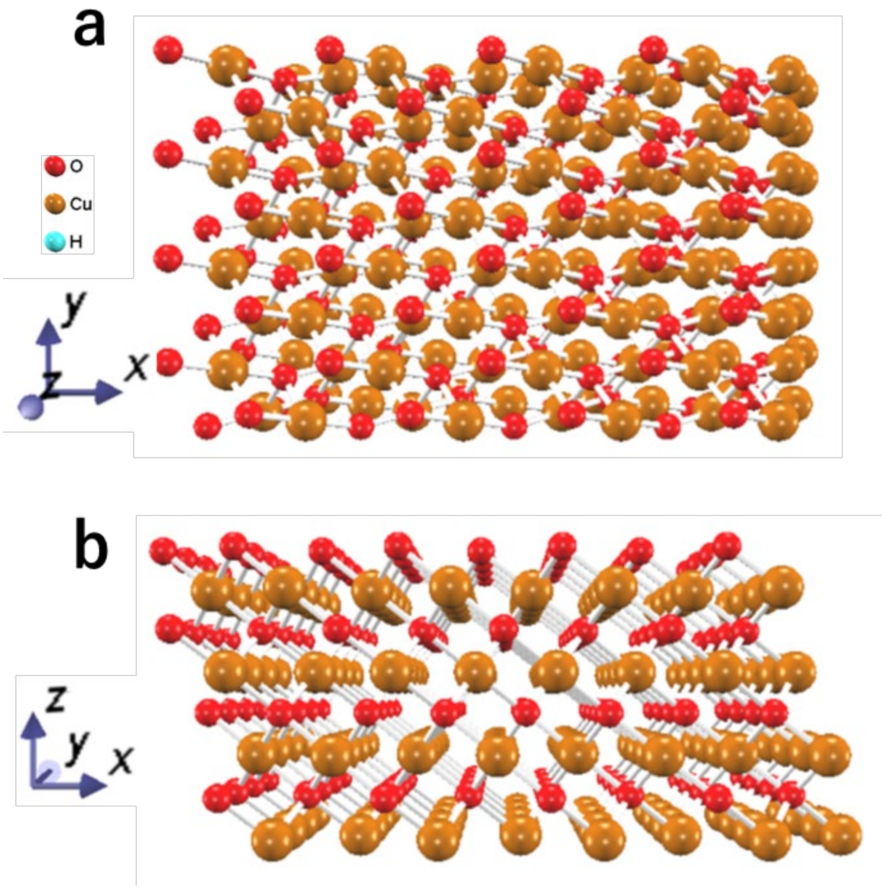

Fig. S17. Schematic representation of the CuO slab model consisted of 256 Cu and O atoms (32 x 8) used for hydrogen solution. **a**. Top view; **b**. Side view.

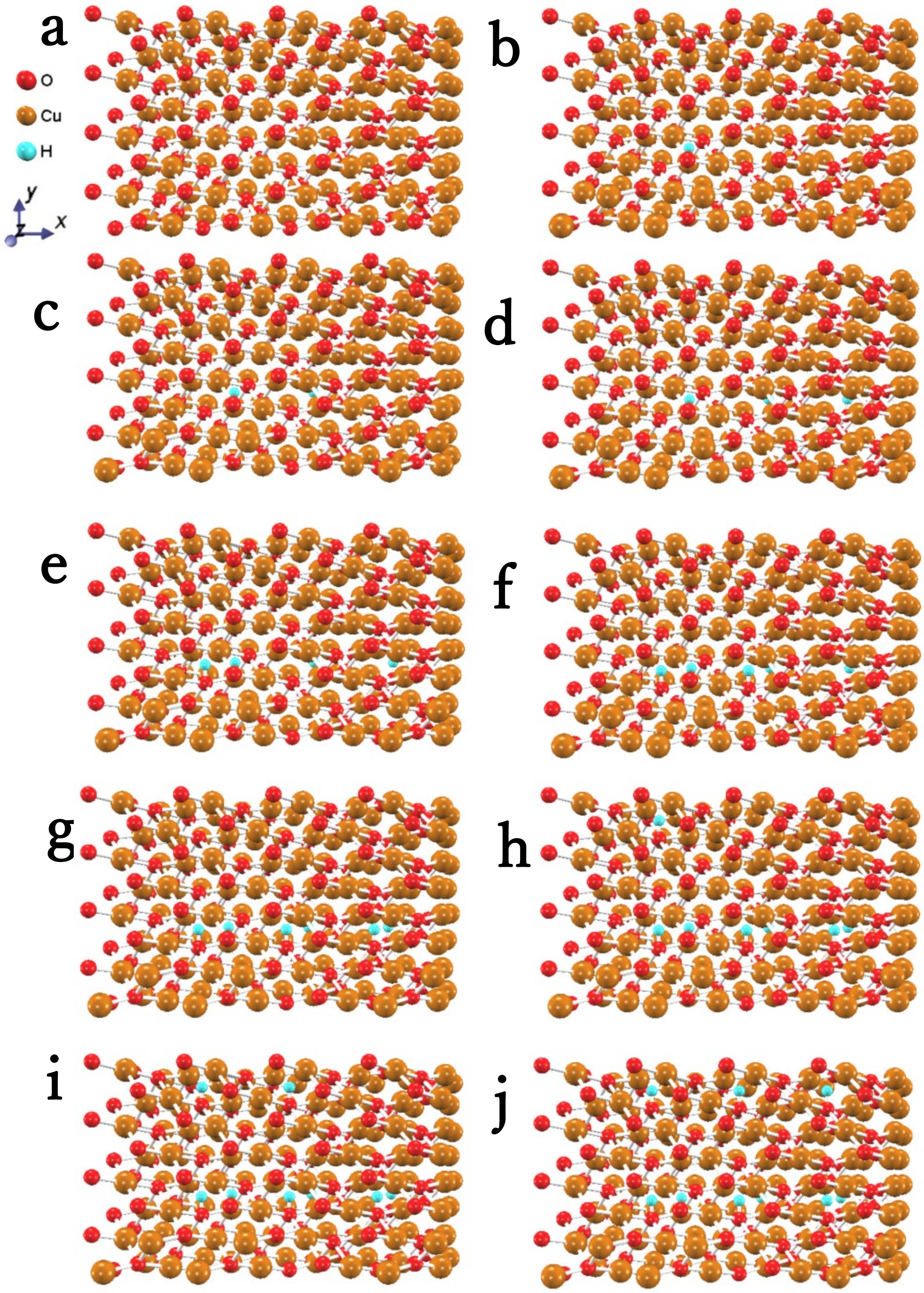

Fig. S18. Optimized structures of CuO with H atoms inserted into interstitial octahedral sites. **a.** CuO slab model consisted of 256 Cu and O atoms before inserting H; **b.** the CuO with inserting one H; **c.** the CuO with two H inserting; **d.** the CuO with inserting three H; **e.** the CuO with inserting four H; **f.** the CuO with inserting five H; **g.** the CuO with inserting six H; **h.** the CuO with inserting seven H; **i.** the CuO with inserting eight H; **j.** the CuO with inserting nine H.

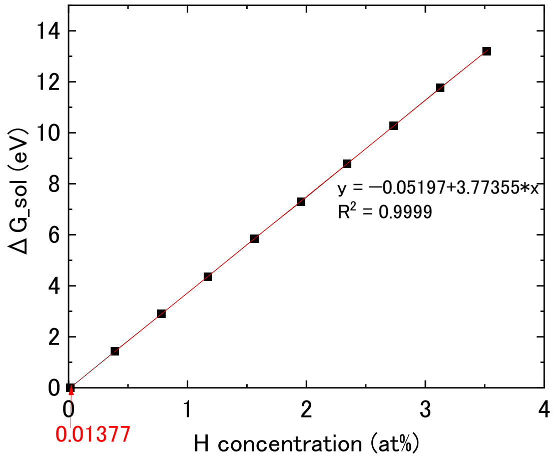

Fig. S19. Solubility energy (ΔG_sol) at various hydrogen concentrations in CuO at 298 K and 1.27 MPa. These values exhibit excellent linearity. The H solubility at ΔG_sol = 0 was determined as 0.01377 at% by extrapolation.

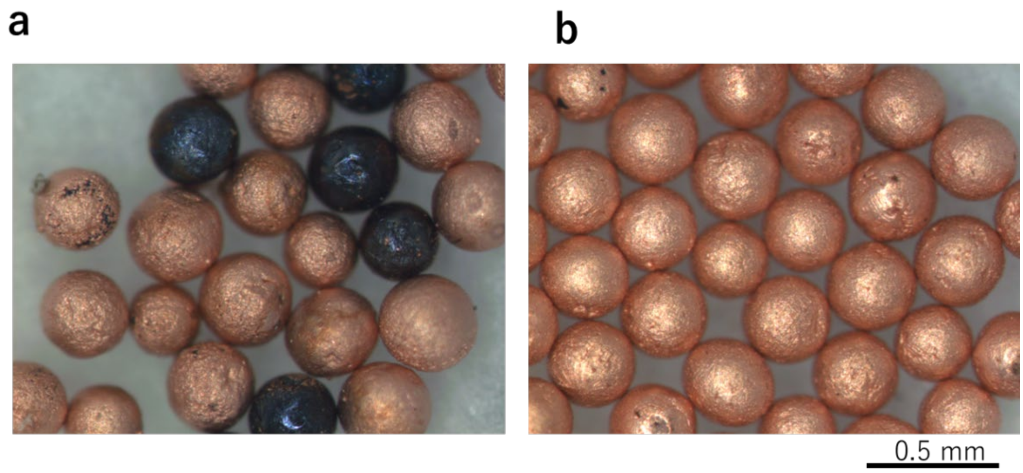

Fig. S20. Stereomicroscope observation of Cu plated $ErCo_2$ particles without (**a**) and with (**b**) pre-cleaning by immersion in 4 vol% HCl aqueous solution at 298 K for 20 seconds.

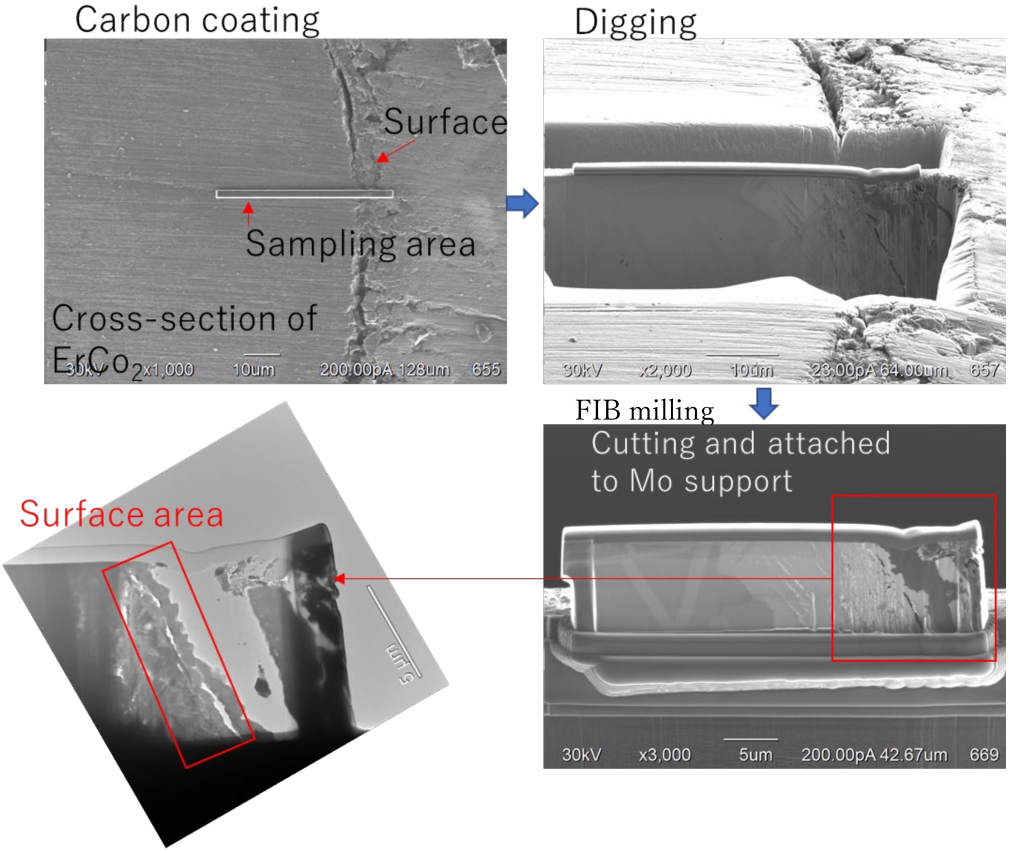

Fig. S21. Preparation of Cu-plated/oxidized $ErCo_2$ cross-sectional sample for TEM analysis by focused ion beam (FIB) technique.

Table S1. long-term hydrogen exposure test results of oxidized, Cu-plated_0.17h/oxidized, and Cu-plated_1.5h/oxidized $ErCo_2$ particles around room temperature.

Sample	Initial pressure P_i (MPa)	Finial pressure P_f (MPa)	Test period (h)	Decrease rate $((P_i-P_f)/P_i)$ (%)
oxidized	1.314	1.287	677.75	2.055
Cu-plated_0.17h/oxdized	1.277	1.266	575.67	0.861
Cu-plated_1.5h/oxdized	1.276	1.253	679.37	1.803

Table S2_1. Adsorption energies of H_2 on various sites of CuO(111) shown in Fig. S6a with orientations perpendicular or parallel to the surface, and the H-H distance and positions relative to CuO(111) surface in the initial state and after optimization. H-Cu* distance is the nearest distance between H-atom in H_2 and Cu-atom in surface; H-O* distance is the nearest distance between H-atom in H_2 and O-atom in surface. The optimization calculations were performed under the condition that the positions of Cu and O atoms were fixed and only the H atoms were mobile. The "x", "y", and "z" in the first left column mean that the orientation of H_2 is parallel to the x-axis, y-axis, and z-axis, and the "p" and "v" mean that the orientation of H_2 is parallel or vertical to the orientation of bridge. The initial positions with "+" mark in the first left column were used for the CI-NEB calculation. All values of adsorption energy are corrected using zero-point energy (ZPE).

Initial position (The No. correspond to Fig. S6a)	Adsorption energy (kJ/mol)	Initial H-H distance (Å)	Optimized H-H distance (Å)	Initial H-Cu* distance (Å)	Optimized H-Cu* distance (Å)	Initial H-O* distance (Å)	Optimized H-O* distance (Å)
①x^+	2.707934622	0.74	0.7515	2.4435	3.7804	1.5449	2.8749
①y^+	0.43992425	0.74	0.7494	2.5748	4.4898	1.5428	3.8017
①z^+	1.871749617	0.74	0.7517	2.6701	3.6772	1.4999	2.7803
②x	0.843295767	0.718	0.7506	1.4548	3.608	1.9293	3.7021
②y	0.812926054	0.764	0.7513	1.4592	3.8867	2.0806	4.0478
②z	0.5364446	0.74	0.7498	1.4084	5.3749	2.1443	4.6426
③x	0.637045318	0.74	0.7502	1.5449	3.5922	2.038	3.772
③y	1.27979685	0.7225	0.7507	1.5428	3.5294	1.8925	3.9238
③z	0.335467166	0.74	0.7523	1.4999	2.8974	2.1314	3.0343
④x	0.064864946	0.74	0.7502	2.154	3.8909	1.5449	3.8894
④y	-3.294733298	0.7225	0.75	2.0131	5.6831	1.5428	6.4442
④z	0.728087047	0.74	0.7502	2.171	3.7657	1.4999	4.1248
⑤p	1.099010752	0.74	0.7533	1.4781	3.7338	1.7281	2.7805
⑤v	1.779113283	0.7401	0.7505	2.0324	3.7897	2.1201	3.3108
⑤z^+	0.963559496	0.74	0.7518	1.5795	3.1664	1.9514	2.8146
⑥p^+	-0.120532688	0.74	0.7484	2.237	5.8932	1.8481	5.7012
⑥v	1.406477671	0.7401	0.7505	2.0324	3.7769	2.1188	3.4277

⑥z	1.129831581	0.74	0.7529	2.2132	3.3876	2.0828	3.0607
⑦p^{+}	1.818227977	0.74	0.7516	1.9478	3.309	1.6842	3.4366
⑦v	-2.32297713	0.7355	0.7523	2.2264	2.8395	1.8861	3.013
⑦z	1.875613505	0.74	0.7514	2.2059	3.4554	1.8883	3.0494
⑧p	3.131155569	0.74	0.7523	1.8463	2.9487	2.0422	3.1516
⑧v^{+}	0.675254069	0.7401	0.7508	2.1199	4.0821	1.8563	4.3693
⑧z^{+}	2.39417099	0.74	0.7535	2.084	3.1623	2.1396	3.1792
⑨p	1.50766897	0.7377	0.7535	2.0887	3.9485	1.2819	3.3665
⑨v^{+}	0.170919028	0.74	0.7501	1.8534	4.5595	1.5839	5.4809
⑨z^{+}	2.024102632	0.74	0.7519	2.0924	3.4521	1.5501	2.7609
⑩p	1.238015837	0.74	0.7532	2.1556	3.2244	1.5144	3.2232
⑩v^{+}	0.59175944	0.7406	0.7497	1.7733	4.131	1.7409	3.7328
⑩z	1.411244316	0.74	0.7533	2.0428	3.2458	1.6728	3.2013
⑪p	-8.235262292	0.7339	0.7507	1.7087	4.3817	2.0092	4.791
⑪v	-2.376663477	0.7354	0.7521	1.986	2.9402	2.2177	3.838
⑪z	0.19903389	0.74	0.7502	1.918	3.3659	2.6378	3.6293
⑫p	1.428446637	0.7213	0.7516	1.4946	3.6421	1.9152	3.5174
⑫v	1.797904254	0.7447	0.7513	1.6232	3.55	2.0137	3.1198
⑫z^{+}	0.285090098	0.74	0.7501	1.6124	4.8206	1.9898	5.0264
⑬p	2.133478302	0.7284	0.7514	1.7162	3.1679	1.397	2.9367
⑬v	1.593070358	0.7446	0.7511	1.9831	3.09	1.718	3.2656
⑬z	0.702699607	0.74	0.7551	1.9312	6.2165	1.6403	6.0467
⑭p	-6.363200746	0.7432	0.7496	1.4877	4.744	1.842	4.9566
⑭v	2.204570408	0.7438	0.7517	1.6616	2.974	1.9336	3.7046
⑭z	1.175168615	0.74	0.7517	1.6478	3.0083	1.9407	3.4517
⑮p	-7.192028077	0.7648	0.7516	2.0423	3.094	2.2215	3.6984
⑮v	1.26693721	0.7368	0.7512	2.1665	3.2449	2.0836	3.7781
⑮z	1.954403712	0.74	0.7515	2.2047	3.1202	2.2041	3.6268
⑯x	0.093501264	0.7414	0.7503	2.0959	4.1804	1.4149	3.6448
⑯y	0.474061031	0.7419	0.7501	2.0446	4.5016	1.4771	4.3878
⑯z	-1.156994574	0.74	0.7499	2.2796	4.9667	1.434	4.9324
⑰x	0.548805553	0.74	0.7515	2.2122	3.1959	1.563	3.7225
⑰y	-0.789317294	0.7363	0.75	1.9372	5.3728	1.8015	4.7601
⑰z	2.035044246	0.74	0.7514	2.116	3.4541	1.73	3.1281
⑱x	1.572064363	0.74	0.7499	1.9767	6.4263	1.7844	6.0925

⑱y	-0.628455592	0.74	0.7499	1.6619	3.7396	1.9326	3.986
⑱z	2.555772258	0.74	0.7507	1.9378	3.2877	1.9342	3.4295
⑲x	1.03467322	0.74	0.7505	1.6337	3.5851	2.0256	3.8967
⑲y	1.034646965	0.74	0.7505	1.6337	3.5851	2.0256	3.8957
⑲z	1.406298132	0.74	0.7498	1.6301	6.4885	3.7203	6.1456
⑳x	1.709635421	0.74	0.7516	2.12	3.2586	1.4435	3.0626
⑳y	0.230943949	0.74	0.7497	2.067	5.9255	1.4669	6.0579
⑳z	3.80941411	0.74	0.7515	2.0675	3.2961	1.4669	2.977
㉑x	0.952196921	0.74	0.75	1.979	4.5213	1.8304	4.6319
㉑y	1.572434554	0.74	0.7511	2.1408	3.6895	1.8824	3.2829
㉑z	3.333448685	0.74	0.7512	2.1496	3.4838	1.835	3.1172
㉒x	-0.155528342	0.74	0.751	2.0417	4.2253	1.6961	4.8285
㉒y	1.711641945	0.74	0.7505	1.7288	3.5249	1.8322	3.9464
㉒z	2.184117085	0.74	0.75	2.0142	3.8057	1.8435	3.8066
㉓x	1.351608621	0.74	0.7511	1.4167	3.3005	2.1331	3.9374
㉓y	1.74461055	0.74	0.7501	1.4367	3.4053	2.1241	3.8163
㉓z	3.270793208	0.74	0.7519	1.4281	3.4643	2.4997	3.7938

Table S2_2. Adsorption energies of H_2 on various titled sites of CuO(111) shown in Fig. S6a with the orientations at an angle of approximately 45°to the CuO(111) surface, and the H-H distance and positions relative to CuO(111) surface in the initial state and after optimization. H-Cu* distance is the nearest distance between H-atom in H_2 and Cu-atom in surface; H-O* distance is the nearest distance between H-atom in H_2 and O-atom in surface. The optimization calculations were performed under the condition that the positions of Cu and O atoms were fixed and only the H atoms were mobile. The "x45" and "y45" in the first left column mean that the orientation of H_2 is titled approximately 45°toward to z-axis from the initial orientations parallel to the x-axis and y-axis, and the "p45" and "v45" mean that the orientation of H_2 is titled approximately 45°toward to z-axis from the initial orientations parallel or vertical to the orientation of bridge. All values of adsorption energy are ZPE-corrected.

Initial position (The No. correspond to Fig. S6a)	Adsorption energy (kJ/mol)	Initial H-H distance (Å)	Optimized H-H distance (Å)	Initial H-Cu* distance (Å)	Optimized H-Cu* distance (Å)	Initial H-O* distance (Å)	Optimized H-O* distance (Å)
①x45	0.760656009	0.744	0.7497	2.4826	8.3182	1.5449	8.1403
②x45	3.444324007	0.7405	0.7522	1.4547	2.8502	1.9774	3.1643
③x45	3.444324007	0.7432	0.7522	1.5449	2.8502	2.038	3.1643
④p45	3.415944844	0.74	0.7524	2.0857	3.3541	1.397	2.9246
④v45	1.488188323	0.74	0.7498	1.9503	4.9845	1.6453	4.6154
⑤p45	3.900853712	0.7409	0.7522	2.2144	2.982	1.4781	2.8416
⑤v45	3.747557666	0.74	0.752	1.9514	3.1775	1.5795	2.8356
⑥p45	2.145342858	0.74	0.7511	2.2508	3.4321	1.8481	3.0217
⑥v45	0.933600282	0.7408	0.751	2.0324	3.5105	2.1152	3.2063
⑦p45	1.601487865	0.7403	0.7498	1.4328	7.6122	1.6842	7.8149
⑦v45	3.665030315	0.7401	0.7515	2.2264	3.1652	1.8861	3.1502
⑧p45	1.509977335	0.7407	0.7438	1.8463	7.7895	2.2932	7.7895
⑧v45	1.580525112	0.7409	0.752	2.1199	3.1231	2.8162	3.7057
⑨p45	-0.108650449	0.7401	0.7499	2.1595	4.8402	1.2819	4.124
⑨v45	-0.62347521	0.7406	0.7506	1.9084	3.9031	1.5839	3.678
⑩p45	1.669487769	0.7417	0.7511	2.1436	3.387	1.5144	3.5457
⑩v45	1.990806038	0.7475	0.7515	1.7733	3.4024	1.6852	2.97

⑪x45	3.717422031	0.741	0.7512	2.3893	3.5031	1.4149	2.9405
⑪y45	3.643643003	0.7401	0.7513	1.991	3.4594	1.4854	2.833
⑫x45	1.917247422	0.7405	0.7519	2.2197	3.2312	1.563	2.9073
⑫y45	3.058913173	0.7408	0.7517	2.2303	3.3727	1.7353	2.9329

Table S3. Composition of aqueous electroless Cu-plating solution.

**Substances**	**Concentration (mol/dm^3)**
$CuSO_4 \cdot 5H_2O$	0.03
Formaldehyde (37% HCHO)	0.1
NaOH	0.23
EDTA・2Na	0.04
NaCN	$2.04*10^{-4}$
α,α'-Dipyridyl	$6.26*10^{-6}$

Table S4. Thickness of formed surface layer and weight decrease of the particles after different treatment times using a 4 vol% HCl aqueous solution at 298 K. The thickness of surface layer was measured from the SEM images of cross-section of particles. The weight decrease of particles was obtained by measuring the weights before and after treatment. The Er/Co atomic ratio was obtained by EDS analysis of the cross-section of particles.

Treatment time (sec)	Thickness of surface layer (μm)	Weight decrease of particles (wt%)	Er/Co in surface layer (atomic ratio)	Er/Co in substrate (atomic ratio)
20	5～20	7.4	1.5	1.9
600	5～20	23.5	0.7	1.7
