# Supplementary material for: Enhancing hydrogen permeation barrier performance of ErCo2 magnetic refrigeration material via surface oxide layer formation
Source: Nat Commun. 2026 Apr 7;17:4952. doi: 10.1038/s41467-026-71547-0 (PMC13233819; doi:10.1038/s41467-026-71547-0)
Supplement: Supplementary file 2 — Transparent Peer Review file [file 41467_2026_71547_MOESM2_ESM.pdf]

nature portfolio

Peer Review File

Enhancing hydrogen permeation barrier performance of ErCo2 magnetic refrigeration material via formation of a surface oxide layer

Corresponding Author: Dr Ya Xu

This file contains all reviewer reports in order by version, followed by all author rebuttals in order by version.

Attachments originally included by the reviewers as part of their assessment can be found at the end of this file.

Version 0:

Reviewer comments:

Reviewer #1

(Remarks to the Author)
The manuscript is generally well written and presents some interesting findings. However, the interpretation of certain results and their correlation with the material properties require more in-depth analysis and clarification. Additionally, the manuscript appears to be narrowly targeted at specialists in magnetocaloric materials for hydrogen liquefaction, rather than the broader, interdisciplinary audience of Nature Communications. After careful evaluation, I do not find the overall quality and novelty of the work sufficient for publication in Nature Communications. Instead, the paper would be more suitable for a specialized journal focused on magnetocaloric materials, pending substantial revision. Below are detailed comments:

1. Questionable Novelty of the Work
The novelty claimed by the authors is not sufficiently justified. In both the introduction and the final paragraph before the Methods section, the authors state: "we demonstrated a novel method for synthesizing $ErCo_2$ magnetic refrigeration materials with excellent HPB performance." However, the synthesis method employed — EIGA — is a well-established technique, as acknowledged in the authors' own references (26–28). Similarly, electroless Cu plating is a well-known method.
Furthermore, the use of Cu or CuO as hydrogen permeation barriers is not new. For instance: Liu et al. (Corrosion Science 221 (2023) 111314) demonstrated the effectiveness of Cu coatings on carbon steels to reduce hydrogen permeation. And studies involving CuO in combination with other oxides for hydrogen protection of cobalt composite membranes also exist (https://doi.org/10.4028/www.scientific.net/DDF.297-301.483).
The mechanism proposed in the manuscript — that Cu-based coatings act as hydrogen barriers — is consistent with prior studies. Therefore, it is unclear how the authors justify the novelty of their approach. Although the results may be of interest to researchers in hydrogen liquefaction, the current level of innovation does not meet the standard of Nature Communications.

2. Inadequate Evaluation of Magnetocaloric Performance
The manuscript provides very limited data on the magnetocaloric properties of the materials. For example:
• Only M–T curves under a low magnetic field are presented. Why are there no M–T curves under varying fields up to 5 T?
• Key performance indicators such as thermal hysteresis changes, adiabatic change of temperature, or consideration on how heat exchange is influenced after surface modifications are not discussed.
• The microstructural data indicate that the protective surface layer is under 10 µm, while the particles are approximately 0.3 mm in diameter. How does such a thin layer lead to a 30% reduction in magnetic entropy change? The authors' explanations and microstructural evidence do not convincingly support the observed magnetocaloric behavior.

3. Questionable Microstructural Characterization and Interpretation
There are inconsistencies and ambiguities in the microstructural analysis:
• Lines 180–182 claim that "a 20–30 µm thick intermediate layer appeared brighter than the outermost layer but darker than the interior," yet this is not evident in Figure 3.
• The description of the TEM data suggests the formation of "dense CuO with large crystallites," but the images do not

provide sufficient evidence to assess density or crystallite size. Drawing such conclusions from limited local TEM observations is problematic.
• The authors claim material loss during TEM sample preparation due to brittleness. However, numerous studies have successfully prepared brittle materials for TEM without such loss. The missing regions could be due to a weak interface between the oxide and underlying layers, rather than sample preparation artifacts.
4. Incomplete and Unclear Hydrogen Diffusion Analysis
The methodology for assessing hydrogen diffusion is inadequately described:
• The D_2 exposure conditions (e.g., duration, temperature, pressure) are not clearly specified. If they differ from the hydrogen exposure conditions, how can the results be directly compared?
• Why were alternative techniques not employed to quantify hydrogen content and distribution immediately following hydrogen exposure?
• Figure 1 shows some hydrogen absorption even in the Cu-plated/oxidized sample. However, the correlation between hydrogen absorption data and the D_2 exposure/ToF-SIMS measurements is unclear.

In conclusion, the manuscript does not meet the standards for publication in Nature Communications. The novelty is not convincingly demonstrated, key material characterizations are insufficient or inadequately interpreted, and the relevance of some experimental results is not clearly established. I recommend reconsideration for a more specialized journal, contingent upon addressing the points outlined above.

Reviewer #2

(Remarks to the Author)
This manuscript contains some interesting results, which may be useful for researchers working in this field. Generally the article is well organized, the authors quite well refer to other literature data.
In my opinion, this article is suitable for publication. However, there are some minor attention which must be addressed before the final decision. Below I present some minor comments.

1. Is ΔS_M the only parameter considered when selecting a suitable magnetocaloric material? What about ΔT_{ad} ? I believe that the introduction should present the magnetocaloric properties of the studied compound in detail — that is, all available data from the literature, not only ΔS_M .
2. In what type of cooling cycle is the proposed refrigerant intended to be used? It might be worth enriching the introduction with reports on other types of magnetocaloric materials, for example, in layered form. It should be added in the introduction that in various forms magnetocaloric materials as promising materials for helium or hydrogen liquefaction are studied, e.g. as composites, for example pleas take a look at recent work: DOI: 10.1039/d4tc02247e or doi.org/10.1016/j.ijhydene.2024.09.082
3. Under what conditions was M(T) measured? ZFC or FC?
4. The temperature sweep rate for the presented M(T) curves should be given. What was the mass of the samples for magnetic measurements ? Were isothermal magnetization curves for the samples studied corrected due to the internal demagnetizing field ?
5. The presented $\Delta S_M(T)$ dependencies generally indicate that ΔS_M decreased after applying the proposed barrier. This should be clearly emphasized. Moreover, the figure shows that after exposure, ΔS_M increases. This fact should be explained. What was the measurement error of ΔS_M?
6. Fig.1a no description of the insert.

I will make a further decision after responding to the above comments.

Reviewer #3

(Remarks to the Author)
In this manuscript titled “Enhancing hydrogen permeation barrier performance of $ErCo_2$ magnetic refrigeration material via surface oxide layer formation”, the authors investigate $ErCo_2$ as a magneto-caloric material for magnetic refrigeration, with potential application in liquid hydrogen production. Due to their small atomic radius, hydrogen atoms can easily diffuse into host materials, leading to property degradation and, in severe cases, hydrogen embrittlement. To address this, the authors explore the hydrogen permeation barrier (HPB) performance of $ErCo_2$ particles by applying electroless copper plating followed by oxidation, resulting in a CuO layer several micrometers thick. This surface modification is shown to effectively suppress hydrogen ingress and maintain the structural integrity of the material. The study addresses an important challenge in the development of hydrogen-compatible magnetic refrigeration materials and provides useful data regarding coating-based hydrogen permeation barrier (HPB) strategies. While the manuscript reports encouraging results, the methods and techniques employed are largely established, and thus the level of technical innovation is limited. Unless these substantive issues are addressed clearly, the manuscript does not reach the level to be considered for publication in Nature Communications.

1. Why was electroless Cu plating chosen? The authors should explain why coatings with inherently low hydrogen diffusivity (e.g., Al_2O_3, Y_2O_3, Er_2O_3, La_2O_3, or SiO_2) were not used. It is not enough to say that PVD/CVD methods struggle to form uniform HPB layers on sub-500 μm $ErCo_2$ particles, please provide a thorough explanation/justification. Moreover, electroless Ni or Co plating is also feasible.

2. The authors should conduct further experimental tests on H2 permeation of the Cu-plated/oxidized particles, including pressure–composition–temperature (PCT) curves, intrinsic hydrogen diffusion coefficients, hydrogen permeation flux measurements, and in particular long-term hydrogen permeation stability.

3. The manuscript does not provide a systematic analysis of how different Cu coating thicknesses and the formation of surface oxides thicknesses influence hydrogen permeation behavior, nor does it address the potential impact on the magnetocaloric effect of ErCo2. Include thickness-dependent investigations to better understand the impact on functional performance.

4. Why did the authors choose an initial hydrogen pressure of approximately 1.27 MPa, rather than a different or higher value (e.g., 3 MPa)? Is there a specific thermodynamic or kinetic consideration behind this choice?

5. In Fig. 1a and 1b, the H_2 absorption curves for the oxidized ErCo2 particles and the Cu-plated/oxidized particles do not intersect under the presented exposure times. Could the authors extend the H2 exposure duration to check whether these curves might intersect or partially overlap at longer times? Additionally, would simply oxidizing the ErCo2 surface (without Cu plating) be sufficient to slow hydrogen permeation while causing only a minimal decrease in magnetization and magnetic entropy change?

6. In lines 102 ~ 104, the authors infer that ErCo2 particles were fully penetrated and completely transformed into hydrides based solely on stereomicroscope observations of particle fragmentation. This conclusion may be premature, as morphological changes alone are insufficient to confirm complete hydrogenation.

7. Why weren't the magnetization and magnetic entropy change measured for the oxidized ErCo2 particles or those exposed to H2, as shown in Fig. 1c and 1d? These data would allow a clearer comparison with the as-homogenized and Cu-coated/oxidized samples, and help clarify how surface oxidation affects magnetocaloric performance.

8. After the Cu plating–oxidation treatment on ErCo2, the magnetic entropy change (ΔSM) decreased significantly from 33 $J{\cdot}kg^{-1}{\cdot}K^{-1}$ to 24 $J{\cdot}kg^{-1}{\cdot}K^{-1}$. What is the underlying reason for this reduction? Furthermore, is it possible to design surface treatments (e.g., coatings or modifications) that can preserve or even enhance the magnetic entropy change while still effectively preventing hydrogen embrittlement?

9. For the SEM (JEOL JSM-7000F, JSM-6500F) and TEM (JEOL JEM-ARM200F) characterizations coupled with X-ray EDS, could the authors please specify the accelerating voltage and beam current used?

10. The manuscript mentions that TOF-SIMS analysis was performed using a 30 kV Ga+ ion source for sputtering the surface of ErCo2 particles. However, the primary ion beam current was not reported. Could the authors clarify the beam current used during analysis? This parameter critically affects sputtering rate, signal sensitivity, and surface damage, especially when analyzing non-planar particle surfaces.

11. In the DFT calculations, the authors did not specify the energy convergence threshold and the force convergence criterion used during the slab model optimization. Similarly, the convergence settings applied in the CI-NEB (climbing image nudged elastic band) calculations were not given. It is recommended that the authors provide these critical computational parameters to ensure the reproducibility and reliability of the results.

12. In line 187, the statement "forming an initial Co-rich oxide layer (Fig. S5)" seems incorrect, because the oxide layer is more likely Er-rich according to the EDS results shown in Fig. S5c.

13. In line 230, the sentence "while the 59Co intensity remained at similar levels" appears to be inaccurate. According to Fig. 5c, the ToF-SIMS depth profile of 59Co shows an initial increase followed by a decrease as the analysis progresses into deeper layers. The authors are advised to revise this description to more accurately reflect the observed trend.

14. In Fig. 5b, there appears to be a typo in the y-axis label — "od" should likely be "of". The authors are advised to correct this for clarity and accuracy.

15. In lines 221–223, the statement "Beneath this layer, up to ~20 μm, the peak intensities of 167Er and 16O increased, while that of 59Co decreased" appears to be inaccurate. According to the ToF-SIMS depth profiles shown in Fig. 5b, the intensities of 167Er, 16O, and 59Co seem to remain relatively constant or slightly decrease in the 3–20 μm depth range. Please have the author confirm again.

16. In lines 234–235, the authors state, "while the 167Er and 16O signal intensities remained constant up to ~20 μm." However, in Fig. 5c both 67Er and 16O intensities clearly show a gradual decline with increasing depth up to ~20 μm. Could the authors please clarify or correct this description so that it accurately reflects the observed trend?

17. In Fig. 5d, the normalized D (deuterium) yield for the Cu-plated/oxidized ErCo2 particles shows fluctuations between approximately 15 μm and 26 μm depth, whereas one would expect the D signal to be zero beyond the oxidation layer. Could the authors please explain the origin of these D-signal fluctuations in that depth range and clarify why D is still detected where it should theoretically be absent?

18. In the DFT calculations, only the adsorption and dissociation of a single H2 molecule on the CuO(111) surface were

considered. However, under experimental conditions multiple H_2 molecules adsorb, dissociate, and diffuse concurrently, leading to coverage-dependent interactions. Could the authors discuss how multi-molecule effects (e.g., surface coverage, H–H lateral interactions, and diffusion pathways) might alter the adsorption/dissociation energetics? Would considering cluster models, larger supercells, or molecular dynamics simulations be necessary to capture these effects?

19. To accurately predict H_2 adsorption and dissociation on CuO(111), zero-point energy corrections should be included.

20. In the subtitle of Fig. 6a, the phrase "Optimized positions of H_2 molecules at all initial adsorption sites (shown in Fig. S4a)" appears to be incorrect—shouldn't it refer to Fig. S6a instead? Additionally, the wording "showing that all H_2 molecules moved away from the CuO(111) surface, to a distance exceeding 2.6 Å" seems inconsistent with the data, as the distances actually exceed 2.8 Å. Please verify and correct these details.

21. Three molecular orientations were considered: one perpendicular to the CuO(111) surface and two parallel, but the tilted side-on orientation was not examined. Could the authors comment on whether this tilted geometry might be energetically favorable, and if so, please provide its adsorption energy and optimized structure.

22. In lines 312~314, for the statement "For each measurement, 0.5 g of particles was used. Hydrogen gas was introduced at an initial pressure of ~1.27 MPa after vacuuming with a rotary pump for at least 15 minutes," could the authors clarify whether the samples were first purged with an inert gas to remove residual air? Additionally, what vacuum pressure was reached before introducing hydrogen?

23. In the caption of Fig. 6, the description for subfigure (c) is missing.

24. DFT calculations indicate a high barrier for H diffusion into the CuO(111) subsurface. However, the authors could also calculate hydrogen solubility in the CuO layer to show that it is low, further supporting its enhanced resistance to hydrogen embrittlement.

Reviewer #4

(Remarks to the Author)
This paper reported the formation of oxide layer on the surface of ErCo2 sphere as hydrogen permeation barrier. By comparing the performance of CuO layer and the in situ generated oxide layer of ErCo2, the authors found that CuO is more efficient for hydrogen permeation protection. This finding may provide some valuable insights for protecting the magnetic refrigeration
materials from hydrogen embrittlement. But there are still some questions needed to be further clarified.
1.Some important content that can emphasize the novelty of this work is suggested to be included as follows
1)Is the hydrogen permeation issue a key challenge faced by the magnetic refrigeration materials?
2)Are there any other reports on developing the hydrogen permeation barrier for magnetic refrigeration material? If so, how about their performance?
3)The background for choosing CuO instead of other materials as the hydrogen permeation barrier should be provided, as there are many materials have been reported as efficient hydrogen permeation barriers, such as ZrO2, Er2O3, α-Al2O3, CrxN, et al. These materials have higher chemical stability in the hydrogen environment than CuO. What is the principle for choosing the alternative hydrogen resistant materials for ErCo2? Except the hydrogen permeation resistance, are there any other characteristics or functional properties required by the barriers?
2.As the hydrogen permeation barrier performance is evaluated by the hydrogen pressure measured in the equipment. The accuracy of the pressure gauge is very important, as the changes of the hydrogen pressure mentioned in the work are 0.01~0.02 MPa, the type of the pressure gauge used and its Instrument detection limit, the average relative error should be given.
3.It seems that∆SM was decreased with CuO layer on the sphere surface, is the thickness of CuO layer affect the ∆SM? Can the CuO thickness be controlled by the plating-oxidation method? If thinner the layer, will the ∆SM have a higher value?
4.The authors have tested the performance of the hydrogen permeation barriers under 1.27 MPa H2 at 296 K for 7 days. Is this pressure, temperature and time agreed with the condition faced by ErCo2 spheres when in practical applications? What is biggest pressure and the longest time for the ErCo2 spheres exposure to H2 toward hydrogen liquefaction? And how about the performance of CuO layer under higher pressures or for expanding times?
5.It is mentioned that the pre-cleaning step was essential for ensuring uniform Cu deposition. What is the reason? A detailed discussion about this phenomenon is absent. As the quality of the CuO layer is the key to determine its performance, therefore, the growth mechanism of Cu on the sphere surface and the method to improve its quality should be demonstrated in the text.
6.In DFT calculations, line 274 "suggesting that H atoms diffuse easily on the CuO surface", this conclusion cannot be easily understand. And how could the authors get the result "the energy barrier for H diffusion from the surface to subsurface was 337.4 kJ/mol"? These results need to be clarified more clearly in order to be understandable for the readers.

Reviewer #5

(Remarks to the Author)
I co-reviewed this manuscript with one of the reviewers who provided the listed reports. This is part of the Nature Communications initiative to facilitate training in peer review and to provide appropriate recognition for Early Career Researchers who co-review manuscripts.

Version 1:

Reviewer comments:

Reviewer #1

(Remarks to the Author)
The authors have addressed all the questions raised before. I would like to recommend the revised manuscript for publication in Nature Communications.

Reviewer #2

(Remarks to the Author)
The authors responded appropriately to my comments. Please find attached the manuscript, where I have marked the incorrect names of compounds in red.

Reviewer #3

(Remarks to the Author)
In the manuscript titled “Enhancing hydrogen permeation barrier performance of ErCo2 magnetic refrigeration material via surface oxide layer formation,” the authors reveal, through experimental analyses and first-principles calculations, the CuO layer serves as an effective hydrogen permeation barrier, enhancing the hydrogen resistance of $ErCo_2$ intermetallic compounds for magnetic refrigeration applications. The revised manuscript shows clear improvement after the first-round revision. However, several technical and formatting issues still need to be addressed.
(1) The “DFT calculation(s)” section contains font size inconsistencies and apparent character encoding errors, likely related to equation formatting.
(2) The authors should carefully correct these issues and conduct a thorough check of the entire manuscript to ensure consistency in formatting, figure captions, symbols, overall presentation, and figure resolution.

Reviewer #4

(Remarks to the Author)
The authors replied the reviewers’comments and carried out additional experiments and calculations. The reviewer agrees for its publication.

Reviewer #5

(Remarks to the Author)
I co-reviewed this manuscript with one of the reviewers who provided the listed reports. This is part of the Nature Communications initiative to facilitate training in peer review and to provide appropriate recognition for Early Career Researchers who co-review manuscripts.

Version 2:

Reviewer comments:

Reviewer #2

(Remarks to the Author)
The authors have revised the manuscript in accordance with my suggestions. The work can now proceed to the next stages of production.

Reviewer #3

(Remarks to the Author)
The revised manuscript has been improved to the level that can be considered for publication.

Open Access This Peer Review File is licensed under a Creative Commons Attribution 4.0 International License, which permits use, sharing, adaptation, distribution and reproduction in any medium or format, as long as you give appropriate credit to the original author(s) and the source, provide a link to the Creative Commons license, and indicate if changes were made.
In cases where reviewers are anonymous, credit should be given to 'Anonymous Referee' and the source.
The images or other third party material in this Peer Review File are included in the article's Creative Commons license, unless indicated otherwise in a credit line to the material. If material is not included in the article's Creative Commons license and your intended use is not permitted by statutory regulation or exceeds the permitted use, you will need to obtain permission directly from the copyright holder.
To view a copy of this license, visit https://creativecommons.org/licenses/by/4.0/

RESPONSE TO REVIEWERS' COMMENTS

Reviewer #1 (Remarks to the Author):

The manuscript is generally well written and presents some interesting findings. However, the interpretation of certain results and their correlation with the material properties require more in-depth analysis and clarification. Additionally, the manuscript appears to be narrowly targeted at specialists in magnetocaloric materials for hydrogen liquefaction, rather than the broader, interdisciplinary audience of Nature Communications. After careful evaluation, I do not find the overall quality and novelty of the work sufficient for publication in Nature Communications. Instead, the paper would be more suitable for a specialized journal focused on magnetocaloric materials, pending substantial revision. Below are detailed comments:

1. Questionable Novelty of the Work
The novelty claimed by the authors is not sufficiently justified. In both the introduction and the final paragraph before the Methods section, the authors state: "we demonstrated a novel method for synthesizing $ErCo_2$ magnetic refrigeration materials with excellent HPB performance." However, the synthesis method employed — EIGA — is a well-established technique, as acknowledged in the authors' own references (26–28). Similarly, electroless Cu plating is a well-known method.
Furthermore, the use of Cu or CuO as hydrogen permeation barriers is not new. For instance: Liu et al. (Corrosion Science 221 (2023) 111314) demonstrated the effectiveness of Cu coatings on carbon steels to reduce hydrogen permeation. And studies involving CuO in combination with other oxides for hydrogen protection of cobalt composite membranes also exist (https://doi.org/10.4028/www.scientific.net/DDF.297-301.483).
The mechanism proposed in the manuscript — that Cu-based coatings act as hydrogen barriers — is consistent with prior studies. Therefore, it is unclear how the authors justify the novelty of their approach. Although the results may be of interest to researchers in hydrogen liquefaction, the current level of innovation does not meet the standard of Nature Communications.

Response: We appreciate reviewer #1's thorough evaluation of our manuscript. We acknowledge that the following statement insufficiently justifies the novelty of our work: "we demonstrated a novel method for synthesizing $ErCo_2$ magnetic

refrigeration materials with excellent HPB performance." The novelty of our work is on the synthesis $ErCo_2$ magnetic refrigeration materials with excellent HPB performance using well-established techniques, i.e., EIGA, Cu plating, and oxidation treatment.

The literature cited by reviewer #1 (Corrosion Science 221 (2023) 111314) reported the effectiveness of Cu coatings on carbon steels to reduce hydrogen permeation. However, we confirmed in our study that the Cu coating alone on magnetic refrigeration materials is not effective to reduce hydrogen permeation. In fact, we investigated the HPB performance of Cu-plated $ErCo_2$ without oxidation during the same hydrogen exposure test as for the Cu-plated/oxidized $ErCo_2$ and found that the Cu-plated $ErCo_2$ particles showed similar HPB behavior as as-homogenized $ErCo_2$ particles, demonstrating that Cu coating alone is insufficient as a hydrogen permeation barrier for magnetic refrigeration materials.

As for the use of CuO as a hydrogen permeation barrier: although the literature cited by the reviewer #1 reported the hydrogen permeation rate of Al_2O_3–CuO–ZnO-based cobalt composite membrane (https://doi.org/10.4028/www.scientific.net/DDF.297-301.483), there was no indication that CuO might be a potential hydrogen barrier material. To our knowledge, there is no literature reporting CuO as a hydrogen barrier material, either experimentally or through theoretical calculations. Therefore, this work is clearly novel.

We revised the related description to clarify more the novelty in the Introduction and final paragraph.

(In the Introduction)

"Compared to these coating methods, formation of CuO layer by copper plating followed by oxidation treatment offers great advantages in terms of cost and efficiency. CuO has not previously been recognized as an HPB material, and this study has demonstrated for the first time that it possesses sufficient HPB properties near room temperature."

(In the final paragraph)

"We successfully synthesized an $ErCo_2$ magnetic refrigeration materials with excellent HPB performance at 1.27 MPa and ~296 K through electroless Cu plating followed by oxidation."

2. Inadequate Evaluation of Magnetocaloric Performance

The manuscript provides very limited data on the magnetocaloric properties of the materials. For example:

• Only M–T curves under a low magnetic field are presented. Why are there no M–T curves under varying fields up to 5 T?

Response: We measured the M–T curves under varying fields up to 5 T for the as-homogenized, oxidized, and Cu-plated/oxidized samples before and after H_2 exposure (Fig. r1) to calculate magnetic entropy change (ΔS_M) using Eq. 3. In the revised manuscript, we have cited the following M–T curves under various magnetic fields up to 5 T for these samples and compiled them in the Supplemental Information (Fig. S).

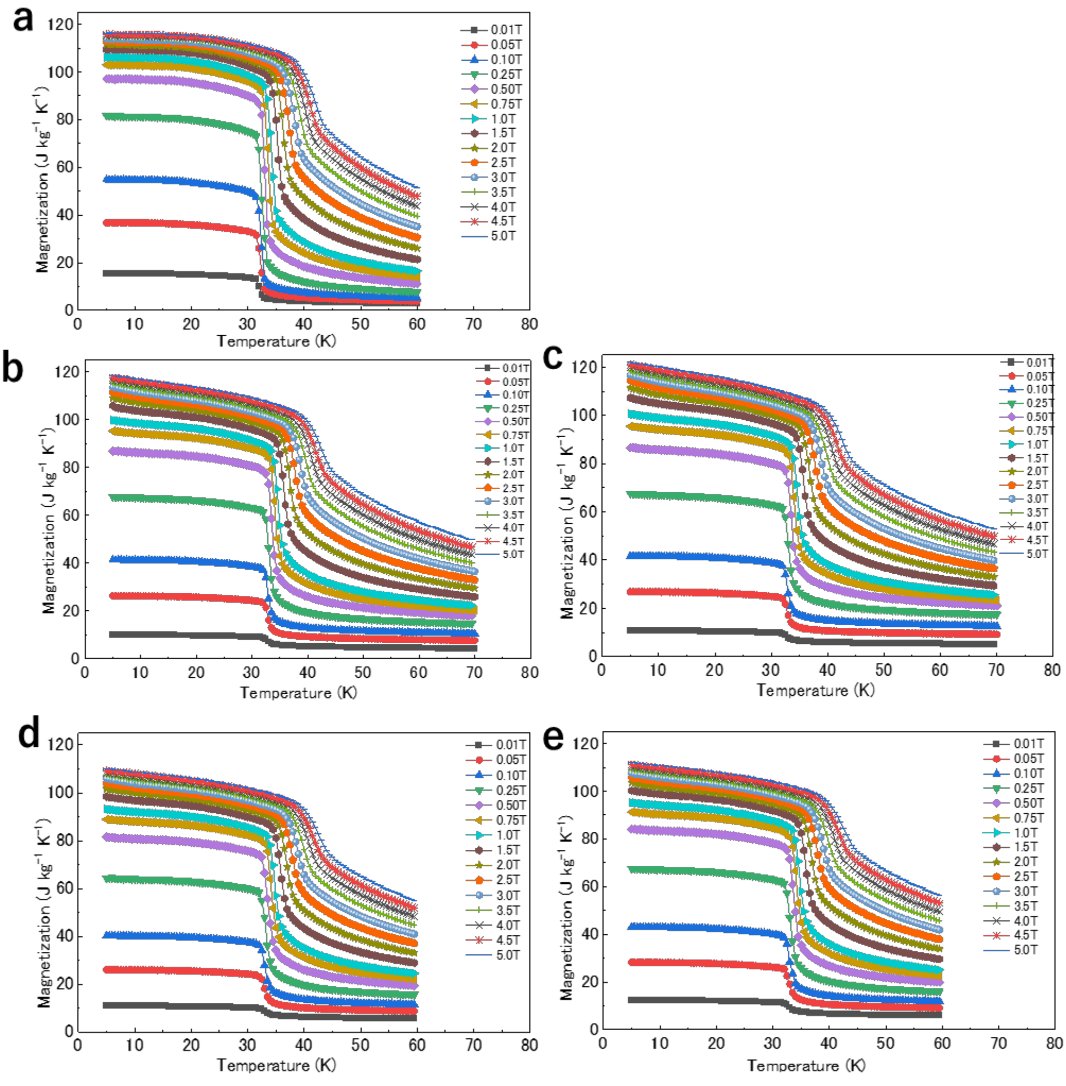

Fig. r1. M–T curves under varying field up to 5 T for the as-homogenized $ErCo_2$ (**a**),

oxidized $ErCo_2$ before H_2 exposure (**b**) and after H_2 exposure (**c**), and Cu-plated/oxidized $ErCo_2$ H_2 exposure (**d**) and after H_2 exposure (**e**).

• Key performance indicators such as thermal hysteresis changes, adiabatic change of temperature, or consideration on how heat exchange is influenced after surface modifications are not discussed.

Response: We certainly recognize the importance of evaluating key performance indicators beyond magnetic entropy change (ΔS_M), as the reviewer pointed out. However, this manuscript focuses on enhancing the HPB performance of magnetocaloric materials and evaluate the HPB performance of the prepared samples. Therefore, this study focused on the effect of HPB coating on ΔS_M, the most fundamental performance metric for magnetocaloric materials. Evaluating other metrics is considered a promising direction for future research. These points are emphasized in the revised manuscript.
We also acknowledge the importance of clarifying the impact of the surface modification on heat exchange. We have assessed its impact by measuring the thermal diffusivity of $ErCo_2$, oxidized $ErCo_2$, and Cu-plated/oxidized $ErCo_2$ plate with a size of approximately 6×6×1 mm^3 at 300 K using a laser flash apparatus (NETZSCH, LFA 467HyperFlash). The difference in thermal diffusivities of these samples was less than 2% (Table r1), which falls below the device's maximum measurement error (3%), indicating that surface modification (Cu plating and oxidation treatment) does not significantly affect the thermal diffusivity of $ErCo_2$ plates. However, since this device cannot measure the thermal diffusivity of micro-spherical samples, directly evaluating the effect of surface modification on the thermal diffusivity of the small $ErCo_2$ particles remains a future challenge.

Table r1. Thermal diffusivity of $ErCo_2$, oxidized $ErCo_2$, and Cu-plated/oxidized $ErCo_2$ plates (The plate size is 6×6×1 mm^3).

Sample	Thermal diffusivity at 300 K (10^{-6} m^2 s^{-1})
As-homogenized $ErCo_2$	3.57
Oxidized $ErCo_2$	3.54
Cu-plated/oxidized $ErCo_2$	3.62

• The microstructural data indicate that the protective surface layer is under 10 μm, while the particles are approximately 0.3 mm in diameter. How does such a thin layer lead to a 30% reduction in magnetic entropy change? The authors' explanations and microstructural evidence do not convincingly support the observed magnetocaloric behavior.

Response: The reduction in ΔS_M is due to the decrease of the weight fraction of $ErCo_2$ main phase in the $ErCo_2$ particle after Cu-plating and/or oxidation treatments. As the ΔS_M data in the present study are shown in unit of J per K per total mass of $ErCo_2$ particles, its magnitude decreases proportionally to the weight fraction of the $ErCo_2$ phase. From the microstructural observation data, we obtained an average thickness of each phase by measuring 3–5 particles and evaluated the weight fraction of each phase in the $ErCo_2$ particle: the innermost $ErCo_2$ phase, the Er–Co–O layer, the Co–O layer, and the outermost CuO layer (Fig. r2). The results show that the decrease of the weight fraction of the $ErCo_2$ phase is approximately 29% after Cu-plating and oxidation, which roughly corresponds to the 30% decrease in magnetic entropy change. For the oxidized-only sample, we also confirmed that the decrease of the weight fraction of the $ErCo_2$ phase corresponds to the decrease in magnetic entropy via the same process (Fig. r3). We have added the above explanation in the revised manuscript and Figs. r2 and r3 in the Supporting Information.

"The reduction in ΔS_M is due to the decrease of the weight fraction of $ErCo_2$ main phase in the $ErCo_2$ particle after Cu plating and/or oxidation treatments. As the ΔS_M data in the present study are shown in unit of J per K per total mass of $ErCo_2$ particles, its magnitude decreases proportionally to the weight fraction of the $ErCo_2$ phase. From the microstructural analysis results shown in Fig. 3, we obtained an average thickness of each phase by measuring 3–5 particles and evaluated the weight fraction of each phase in the $ErCo_2$ particle: the innermost $ErCo_2$ phase, the Er–Co–O layer, the Co–O layer, and the outermost CuO layer (Fig. S7). The weight fraction of the $ErCo_2$ phase decreased by approximately 29% after Cu plating and oxidation, which roughly corresponds to the 30% decrease in magnetic entropy change. For the oxidized-only sample, we also confirmed that the decrease of the weight fraction of the $ErCo_2$ phase corresponds to the decrease in magnetic entropy via the same process (Fig. S8)."

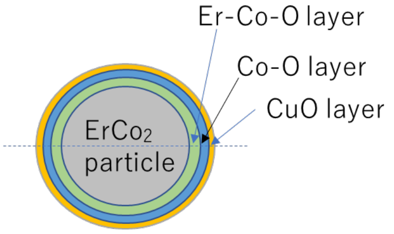

	Thickness (μm)	Volume (cm^3)	Density (g/cm^3)	Mass (g)
$ErCo_2$ Particle	251.6(diameter)	8.33509E-06	10.35	8.62682E-05
Er-Co-O layer	10	2.14989E-06	8	1.71991E-05
Co-O layer	7	1.7064E-06	6.5	1.10916E-05
CuO layer	4	1.06805E-06	6.31	6.7394E-06

Fig. r2. Schematic cross-section of a Cu-plated/oxidized $ErCo_2$ particle, and estimated weight of each layer. For the mass calculation, the density of Er–Co–O layer was assumed to be 8 g/cm^3 which is less than that of $ErCo_2$ (10.35 g/cm^3) but higher than that of CoO (6.5 g/cm^3).

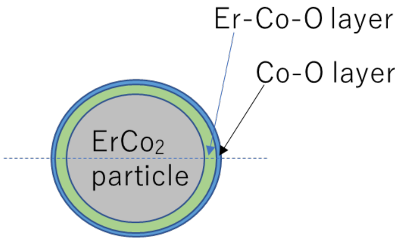

	Thickness (μm)	Volume (cm^3)	Density (g/cm^3)	Mass (g)
$ErCo_2$ Particle	267.6(diameter)	1.00285E-05	10.35	1.03795E-04
Er-Co-O layer	13	3.21632E-06	8	2.57305E-05
Co-O layer	2	5.48751E-07	6.5	3.56688E-06

Fig. r3. Schematic cross-section of a Cu-plated/oxidized $ErCo_2$ particle, and estimated weight of each layer. For the mass calculation, the density of Er–Co–O layer was assumed to be 8 g/cm^3 which is less than that of $ErCo_2$ (10.35 g/cm^3) but higher than that of CoO (6.5 g/cm^3).

3. Questionable Microstructural Characterization and Interpretation

There are inconsistencies and ambiguities in the microstructural analysis:

• Lines 180–182 claim that “a 20–30 μm thick intermediate layer appeared brighter than the outermost layer but darker than the interior,” yet this is not evident in Figure

3.

Response: We appreciate the reviewer for pointing out the typo error. We corrected the thickness of the intermediate layer in the manuscript as follows:
"a 5- to 20-μm-thick intermediate layer appeared brighter than the outermost layer but darker than the interior."

• The description of the TEM data suggests the formation of "dense CuO with large crystallites," but the images do not provide sufficient evidence to assess density or crystallite size. Drawing such conclusions from limited local TEM observations is problematic.

Response: We revised the description of "dense CuO with large crystallites," to "well-crystalized CuO," in the text.

• The authors claim material loss during TEM sample preparation due to brittleness. However, numerous studies have successfully prepared brittle materials for TEM without such loss. The missing regions could be due to a weak interface between the oxide and underlying layers, rather than sample preparation artifacts.

Response: We agree that the missing regions could be due to a weak interface between the oxide and underlying layers. We revised the related description in the manuscript as follows.

"These results indicate that a well-crystalized CuO layer with large crystallites forms on the outer surface, whereas the subsurface Co-, Er-, and Cu-oxide layers exhibit some missing regions, which could be due to a weak interface between the subsurface oxide layer and the outmost surface of the CuO layer."

4. Incomplete and Unclear Hydrogen Diffusion Analysis
The methodology for assessing hydrogen diffusion is inadequately described:
• The D_2 exposure conditions (e.g., duration, temperature, pressure) are not clearly specified. If they differ from the hydrogen exposure conditions, how can the results

be directly compared?

Response: We performed the D_2 exposure experiment at the same temperature and pressure as that for the hydrogen exposure experiments, and the duration was also very close, i.e., 137.5 h for oxidized particles and 139 h for Cu-plated/oxidized particles. To describe it more accurately, the original description in the Methods (lines 316–317) "Similar experiments were conducted using deuterium for subsequent TOF-SIMS analysis." has been amended as follows.

"We performed the D_2 exposure experiment at the same temperature and pressure as that for the hydrogen exposure experiments, and the duration was also very close, i.e., 137.5 h for oxidized particles and 139 h for Cu-plated/oxidized particles."

• Why were alternative techniques not employed to quantify hydrogen content and distribution immediately following hydrogen exposure?

Response: We attempted to quantify the hydrogen content and distribution using ToF-SIMS after hydrogen exposure test. However, clear results could not be obtained probably due to the lower analysis precision of hydrogen compared to deuterium and the difficulty to distinguish between originally existing hydrogen and adsorbed hydrogen in the sample.

• Figure 1 shows some hydrogen absorption even in the Cu-plated/oxidized sample. However, the correlation between hydrogen absorption data and the D_2 exposure/ToF-SIMS measurements is unclear.

Response: Because quantitative analysis of deuterium was difficult by ToF-SIMS, we could not obtain an accurate correlation between the hydrogen absorption data and the D_2 exposure/ToF-SIMS measurements.

Reviewer #2 (Remarks to the Author):

This manuscript contains some interesting results, which may be useful for researchers working in this field. Generally the article is well organized, the authors quite well refer to other literature data.

In my opinion, this article is suitable for publication. However, there are some minor attention which must be addressed before the final decision. Below I present some minor comments.

1. Is ΔSM the only parameter considered when selecting a suitable magnetocaloric material? What about ΔTad ? I believe that the introduction should present the magnetocaloric properties of the studied compound in detail — that is, all available data from the literature, not only ΔSM .

Response: We appreciate reviewer #2's constructive comments, which helped improve the quality of our manuscript. We acknowledge that the adiabatic temperature change ΔT_{ad} is also an important parameter for evaluating magnetocaloric materials. According to previous works [H. Wada et al., Cryogenics 39 (1999) 915-919; J. Alloys Compd. 316 (2001) 245-249.], the ΔT_{ad} of $ErCo_2$ is about 10 K under a 5T-field variation. In the revised Introduction of the manuscript, we have referred to the ΔT_{ad} data available from the literature as follows.

"$ErCo_2$ exhibits a $\Delta S_M > 20$ J kg^{-1} K^{-1} and an adiabatic temperature change (ΔT_{ad}) of ~10 K under a 5 T field change at the Curie temperature of ~32 K[9-11], making it suitable for hydrogen liquefaction."

Added reference:

11. Wada, H., Tanabe, Y., Shiga, M., Sugawara, H. & Sato, H. Magnetocaloric effects of Laves phase Er(CoNi) compounds. J Alloy Compd 316, 245-249, doi:Doi 10.1016/S0925-8388(00)01305-0 (2001).

2. In what type of cooling cycle is the proposed refrigerant intended to be used? It might be worth enriching the introduction with reports on other types of magnetocaloric materials, for example, in layered form. It should be added in the introduction that in various forms magnetocaloric materials as promising materials for helium or hydrogen liquefaction are studied, e.g. as composites, for example pleas take a look at recent work: DOI: 10.1039/d4tc02247e or doi.org/10.1016/j.ijhydene.2024.09.082

Response: The proposed refrigerant is intended to be used in an active magnetic

regenerator (AMR) cycle. We understand that various forms such as composites could be options for the shape optimization of magnetocaloric materials for hydrogen liquefaction via magnetic refrigeration with the AMR cycle. As suggested by the reviewer, we have cited recent works in the Introduction and accordingly modified the sentences. Notably, the method for HPB coating proposed in this study may be applicable not only to particles but also to other material forms. This point has been highlighted in the revised manuscript.

"Magnetic refrigeration offers a more energy-efficient alternative to conventional gas compression/expansion refrigeration technology[4-7], and the efficiency of such refrigeration systems can be further improved using magnetic refrigeration materials with large magnetocaloric effects. Numerous magnetic refrigeration materials with large magnetocaloric effects have been developed, such as $DrNi_2$[8], $ErCo_2$[9-11], $ErNi_2$[12], $Dr_{1-x}Er_xNi_2$[13], HoB_2[14], and $HoAl_2$[15]. However, the development of magnetic refrigeration materials with a large magnetic entropy change (ΔS_M) over a broad temperature range (77–20 K) is challenging[16-20]. A layered composite magnetic refrigerant of HoB_2, $DyNi_2$, and $TbNi_2$ has been developed and obtained an average ΔS_M value of 4.7 J kg^{-1} K^{-1} under a 2 T field change in a broad temperature range of 7.5–53.4 K[21]."

Added references:

8. Cwik, J. *et al.* Magnetocaloric effect in Laves-phase rare-earth compounds with the second-order magnetic phase transition: Estimation of the high-field properties. *Acta Mater* **133**, 230-239, doi:10.1016/j.actamat.2017.05.054 (2017).

12. Llamazares, J. L. S., Ibarra-Gaytán, P., Sánchez-Valdés, C. F., Ríos-Jara, D. & Alvarez-Alonso, P. Magnetocaloric effect in ErNi melt-spun ribbons. *J Rare Earth* **38**, 612-616, doi:10.1016/j.jre.2019.07.011 (2020).

13 Cwik, J. *et al.* Magnetic and magnetocaloric properties of DyErNi solid solutions and their promise for hydrogen liquefaction. *J Mater Chem C* **12**, 14421-14432, doi:10.1039/d4tc02247e (2024).

14 de Castro, P. B. *et al.* Machine-learning-guided discovery of the gigantic magnetocaloric effect in HoB near the hydrogen liquefaction temperature. *Npg Asia Mater* **12**, doi:ARTN 3510.1038/s41427-020-0214-y (2020).

15 Campoy, J. C. P., Plaza, E. J. R., Coelho, A. A. & Gama, S. Magnetoresistivity as a probe to the field-induced change of magnetic entropy in Al compounds (=Pr,Nd,Tb,Dy,Ho,Er). *Phys Rev B* **74**, doi:ARTN 13441010.1103/PhysrevB.74.134410 (2006).

3. Under what conditions was M(T) measured? ZFC or FC?

Response: All the *M*(*T*) measurements were performed under field-cooling processes. This point has been clarified in the Methods section of the revised manuscript.

"All the *M*(*T*) measurements were performed under field-cooling processes."

4. The temperature sweep rate for the presented M(T) curves should be given. What was the mass of the samples for magnetic measurements? Were isothermal magnetization curves for the samples studied corrected due to the internal demagnetizing field ?

Response: The experimental conditions for the *M*(*T*) measurements are as follows: the temperature sweep rate was 0.6 K per min; the mass of the samples was about 3–4 mg. In addition, demagnetization corrections were not performed in this study because it is difficult to evaluate the exact demagnetization factors for a group of the particles. Besides, a previous work [e.g., H. N. Bez et al., J. Magn. Magn. Mater. 458 (2018) 301-309.] has shown that the demagnetization effect does not significantly affect the evaluation of ΔS_M for large magnetic fields such as 5 T, which was the field used for this study. These points have been clarified in the experimental section in the revised manuscript as follows.

"The temperature sweep rate was 0.6 K per min; the mass of the samples was approximately 3–4 mg. Demagnetization corrections were not performed in this study because it is difficult to evaluate the exact demagnetization factors for a group of the particles. The demagnetization effect does not significantly affect the evaluation of ΔS_M for large magnetic fields such as 5 T[44]."

Added References:

"44 Bez, H. N., Yibole, H., Pathak, A., Mudryk, Y. & Pecharsky, V. K. Best practices in evaluation of the magnetocaloric effect from bulk magnetization measurements. J Magn Magn Mater **458**, 301-309, doi:10.1016/j.jmmm.2018.03.020 (2018)."

5. The presented ΔSM(T) dependencies generally indicate that ΔSM decreased after

applying the proposed barrier. This should be clearly emphasized. Moreover, the figure shows that after exposure, ΔSM increases. This fact should be explained. What was the measurement error of ΔSM?

Response: We apologize for the insufficient explanation about the decrease in ΔS_M after applying the proposed barrier. As mentioned in response to reviewer #1's comment, this decrease is due to the reduction in the weight fraction of $ErCo_2$ phase in the particle after the barrier coating (Figs. r2 and r3). The explanation has been added in the revised manuscript.
Meanwhile, the largest measurement error of ΔS_M in our study is due to the weight error of the particle samples used in the measurements, which is estimated to be approximately 1–2%. The difference in ΔS_M before and after hydrogen exposure falls within this error range; therefore, it cannot be concluded that ΔS_M increased after exposure. The following description has been added in the experimental section in the revised manuscript.

"The measurement error of ΔS_M is mainly from the weight error of the particle samples used in the measurements, which is estimated to be approximately 1–2%."

6. Fig.1a no description of the insert.

Response: We added the following description in the caption of Fig. 1a.
"Inset shows the hydrogen pressure change during the initial ten hours."

I will make a further decision after responding to the above comments.

Reviewer #3 (Remarks to the Author):

In this manuscript titled "Enhancing hydrogen permeation barrier performance of $ErCo_2$ magnetic refrigeration material via surface oxide layer formation", the authors investigate $ErCo_2$ as a magneto-caloric material for magnetic refrigeration, with potential application in liquid hydrogen production. Due to their small atomic radius, hydrogen atoms can easily diffuse into host materials, leading to property degradation and, in severe cases, hydrogen embrittlement. To address this, the

authors explore the hydrogen permeation barrier (HPB) performance of $ErCo_2$ particles by applying electroless copper plating followed by oxidation, resulting in a CuO layer several micrometers thick. This surface modification is shown to effectively suppress hydrogen ingress and maintain the structural integrity of the material. The study addresses an important challenge in the development of hydrogen-compatible magnetic refrigeration materials and provides useful data regarding coating-based hydrogen permeation barrier (HPB) strategies. While the manuscript reports encouraging results, the methods and techniques employed are largely established, and thus the level of technical innovation is limited. Unless these substantive issues are addressed clearly, the manuscript does not reach the level to be considered for publication in Nature Communications.

1. Why was electroless Cu plating chosen? The authors should explain why coatings with inherently low hydrogen diffusivity (e.g., Al2O3, Y2O3, Er2O3, La2O3, or SiO2) were not used. It is not enough to say that PVD/CVD methods struggle to form uniform HPB layers on sub-500 µm ErCo2 particles, please provide a thorough explanation/justification. Moreover, electroless Ni or Co plating is also feasible.

Response: We appreciate reviewer #3's insightful and valuable comments, which helped improve the quality of our manuscript. In fact, we have attempted to use materials with low hydrogen diffusivity, such as Al_2O_3 and Cr_2O_3, for forming HPB layer on magnetic refrigeration materials. Specifically, we asked a company to form Cr_2O_3 layer on $ErCo_2$ particles via thermal spraying, however, it was proved difficult to prevent particle aggregation and to control the uniformity of the coating layer on the small $ErCo_2$ particles via thermal spraying. Fig. r4 shows the SEM image of $ErCo_2$ coated with Cr_2O_3 via thermal spraying, indicating that aggregation of particles occurred although the Cr_2O_3 might be formed on the surface.

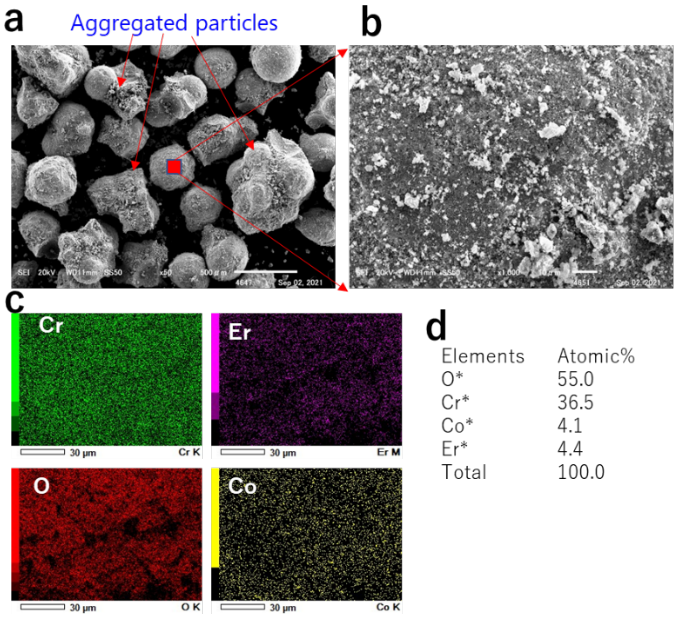

Fig. r4. **a**. SEM image of $ErCo_2$ particles coated with Cr_2O_3 via thermal spraying. **b**. Close-up of the surface of a single particle. **c**. EDS elemental mapping of **b**. **d**. Quantitative analysis results for components in the region of **b**.

Furthermore, we commissioned a company specializing in PVD to form an Al_2O_3 layer onto the $ErCo_2$ particles via a barrel PVD method. Specifically, this involves placing $ErCo_2$ particles within a multi-faceted barrel and rotating the barrel during the coating process. However, it was found difficult to form Al_2O_3 layer uniformly on the surface of small $ErCo_2$ particles with significant surface irregularities.

Compared to the aforementioned methods, copper plating followed by oxidation treatment offers great advantages in terms of cost and efficiency. CuO has not previously been recognized as an HPB material, and this study has demonstrated for the first time that it possesses sufficient HPB properties at temperatures near room temperature.

In addition, regarding the feasibility of electroless Ni or Co plating, we attempted to do electroless Ni plating onto $ErCo_2$ particles. However, due to the acidity of commonly used Ni plating solution, a large amount of Er dissolved during plating, causing the particles to pulverize. However, should a proper alkaline plating solution be achievable, the utilization of Ni plating may be possible. Further research on this matter is ongoing.

We have added the following description in the introduction.

“We attempted to form an Al_2O_3 layer onto the $ErCo_2$ particles via a barrel PVD method. $ErCo_2$ particles were placed within a multi-faceted barrel, which was rotated during the coating process. However, it was difficult to form Al_2O_3 layer uniformly on irregularly surfaced small $ErCo_2$ particles. We also attempted to form Cr_2O_3 layer on $ErCo_2$ particles via thermal spraying. However, particle aggregation is difficult to prevent and the coating uniformity is hard to control on the small $ErCo_2$ particles via thermal spraying.“

2. The authors should conduct further experimental tests on H2 permeation of the Cu-plated/oxidized particles, including pressure–composition–temperature (PCT) curves, intrinsic hydrogen diffusion coefficients, hydrogen permeation flux measurements, and in particular long-term hydrogen permeation stability.

Response: We have performed PCT measurements for the as-homogenized, oxidized, and Cu-plated/oxidized $ErCo_2$ particles. We have also performed the long-term hydrogen exposure tests up to 28 days for the oxidized and Cu-plated/oxidized $ErCo_2$ particles. The following results have been obtained and added in the Results and Discussion section and Supplementary Information.

The pressure–composition–temperature (PCT) measurements were performed for the as-homogenized, oxidized, and Cu-plated/oxidized samples (Fig. r5). For the as-homogenized samples, no obvious hydrogen absorption was observed up to 4 MPa at 303 K, whereas the amount of hydrogen absorption reached approximately 1.3 mass% at 333 K. The PCT results are consistent with the hydrogen exposure measurement results, considering that the PCT measurement process was completed in less than an hour (0.57 h) at 303 K and hydrogen absorption commenced in the as-homogenized particles during the hydrogen exposure experiments (Fig. 1a). In addition, the amount of hydrogen absorption obtained from the PCT measurement at 333 K agrees well with the value of 1.311 mass% obtained from the hydrogen exposure experiment. Furthermore, it was confirmed that the absorbed hydrogen maintained approximately 1 mass% even after the pressure was reduced to a low level (<0.005 MPa), indicating that the hydrogen absorbed by $ErCo_2$ does not desorb easily.

For the oxidized samples, no obvious hydrogen absorption was observed at 303 K, and only a small amount of hydrogen absorption (~0.2 mass%) was observed at 333 K (Fig. r5b). For the Cu-plated/oxidized samples, no apparent hydrogen absorption was observed at both 303 and 333 K (Fig. r5c). These PCT measurements are

consistent with those of hydrogen exposure experiments (Fig. 1a and 1b), indicating that the HPB performance was improved by the oxidation and further enhanced by Cu-plated/oxidation treatment.

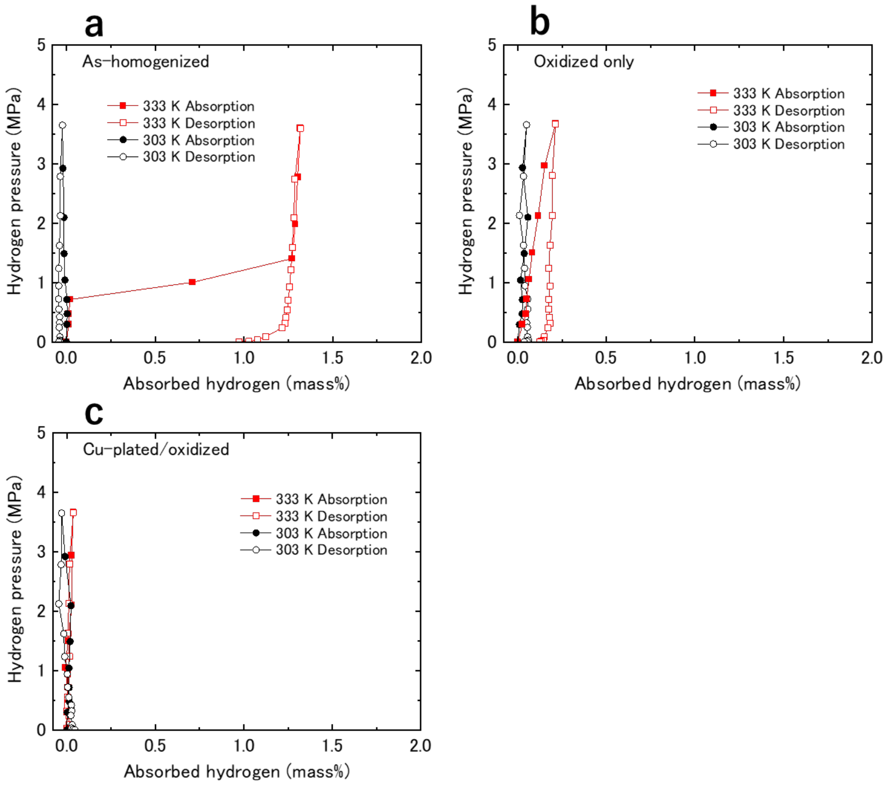

Fig. r5. PCT measurement results of $ErCo_2$ particles. **a**. as-homogenized samples; **b**. samples after oxidization at 773 K for 0.5 h in air (oxidized only); **c**. samples after Cu-plated at 348 K for 0.17 h followed by oxidation at 773 K for 0.5 h in air (Cu-plated/oxidized).

Furthermore, we performed long-term hydrogen exposure experiments at room temperature up to 28 days for evaluating long-term hydrogen permeation stability of oxidized and Cu-plated/oxidized $ErCo_2$ particles. Particles after different Cu-plating time (0.17 and 1.5 h) were used for examining the influence of thickness of CuO layer on HPB performance (Fig. r6). The initial pressure differed slightly among samples due to variations in cell capacity and piping capacity for each sample, that is, 1.314 MPa for oxidized $ErCo_2$, 1.277 MPa for Cu-plated_0.17h/oxidized $ErCo_2$, and 1.276

MPa for Cu-plated_0.17h/oxidized $ErCo_2$. All the three samples underwent hydrogen absorption very slowly during the whole test period, demonstrating that the oxidized and Cu-plated/oxidized $ErCo_2$ have good long-time HPB stability. Specifically, the Cu-plated/oxidized samples exhibit slightly better HPB characteristics than the oxidized sample (Table. r1). Furthermore, the sample with shorter Cu plating time (0.17 h) shows HPB characteristics no less than that with long Cu plating time (1.5 h). As the thickness of CuO layer formed on the sample with Cu plating time of 0.17 h was approximately 1.5 µm (Fig. r7), this result indicates that a 1.5-µm-thick CuO layer can provide a sufficient HPB effect.

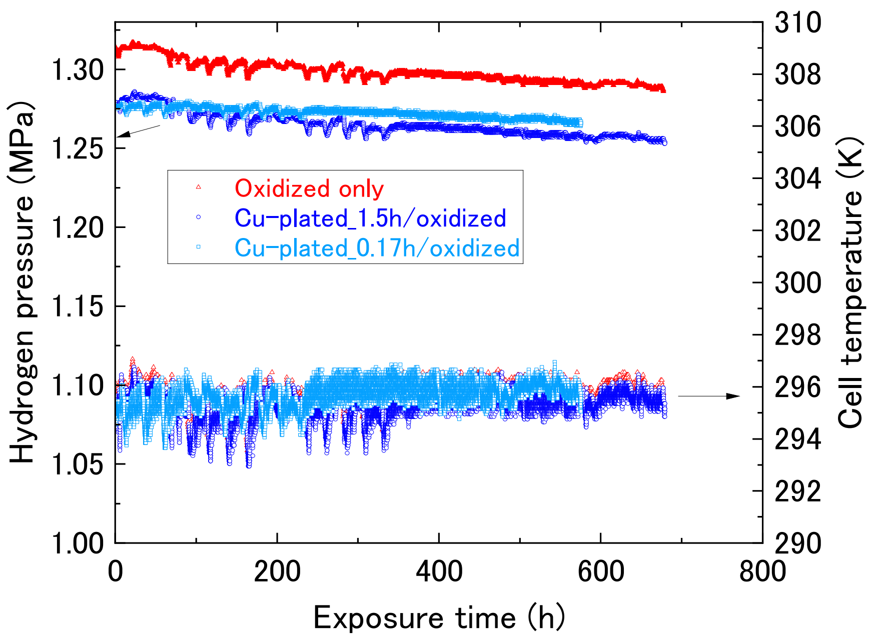

Fig. r6. Hydrogen pressure and cell temperature changes during the long-term hydrogen exposure test for the oxidized-only, Cu-plated_0.17h/oxidized, and Cu-plated_1.5h/oxidized $ErCo_2$ particles. Numerous small pressure fluctuations during the measurement process are due to variations in cell temperature (room temperature).

Table r2. Long-term hydrogen exposure experiment results

Sample	Initial pressure P_i (MPa)	Finial pressure P_f (MPa)	Test period (h)	Decrease rate $((P_i-P_f)/P_i)$ (%)
oxidized	1.314	1.287	677.75	2.055
Cu-plated_0.17h/oxdized	1.277	1.266	575.67	0.861
Cu-plated_1.5h/oxdized	1.276	1.253	679.37	1.803

3. The manuscript does not provide a systematic analysis of how different Cu coating thicknesses and the formation of surface oxides thicknesses influence hydrogen permeation behavior, nor does it address the potential impact on the magnetocaloric effect of ErCo2. Include thickness-dependent investigations to better understand the impact on functional performance.

Response: As mentioned in the response to above comment, we examined the effect of the Cu coating thicknesses and the formation of surface oxide thickness on HPB performance by comparing the results of the samples subjected to a shorter Cu plating time of 0.17 h with those of the samples subjected to a Cu plating time of 1.5 h. Fig. r7 shows the SEM image and EDS line analysis results of the cross-section of the particle with Cu plating for 0.17 h (**a**) and after subsequent oxidization (**b**). The thickness of the Cu layer was approximately 2 µm, and the thickness of the CuO layer was estimated to be approximately 1.5 µm. The HPB performance of the Cu-plated_0.17h/oxidized $ErCo_2$ samples were evaluated by the PCT measurement (Fig. r5d) and the long-term hydrogen exposure test (Fig. r6). These results indicate that a CuO layer of 1.5 µm-thick effectively prevented the hydrogen permeation into $ErCo_2$ particles.

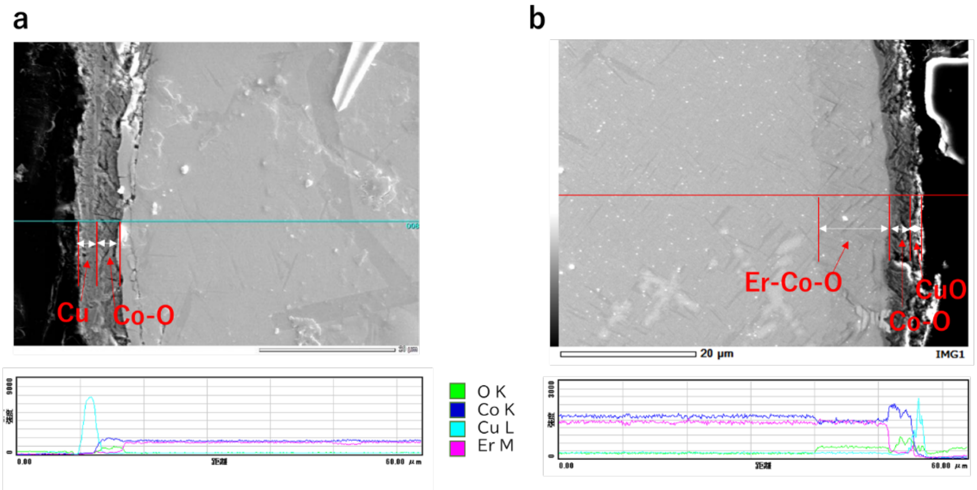

Fig. r7. SEM images and EDS line analysis results of the cross-section of the particle with Cu plating (**a**) for 0.17 h at 348 K (Cu-plated_0.17h $ErCo_2$) and (**b**) after subsequent oxidation treatment (Cu-plated_0.17h/oxidized $ErCo_2$).

4. Why did the authors choose an initial hydrogen pressure of approximately 1.27 MPa, rather than a different or higher value (e.g., 3 MPa)? Is there a specific thermodynamic or kinetic consideration behind this choice?

Response: Gases at 1 MPa or higher are classified as high-pressure gases and fall under the jurisdiction of Japan's High-Pressure Gas Safety Act. Magnetic refrigeration systems offer practical advantages by avoiding this classification. In fact, current magnetic refrigeration systems are designed to operate at pressures below 1 MPa. Therefore, the initial pressure of 1.27 MPa we selected is sufficient for current practical magnetic refrigeration systems. However, higher pressures are advantageous for improving energy efficiency and should be a future research topic.

5. In Fig. 1a and 1b, the H_2 absorption curves for the oxidized ErCo2 particles and the Cu-plated/oxidized particles do not intersect under the presented exposure times. Could the authors extend the H2 exposure duration to check whether these curves might intersect or partially overlap at longer times? Additionally, would simply oxidizing the ErCo2 surface (without Cu plating) be sufficient to slow hydrogen permeation while causing only a minimal decrease in magnetization and magnetic entropy change?

Response: We have performed long-term hydrogen exposure experiments up to 28 days at room temperature for evaluating long-term hydrogen permeation stability of oxidized and Cu-plated/oxidized $ErCo_2$ particles (Fig. r6). Both oxidized and Cu-plated/oxidized $ErCo_2$ particles showed good HPB performance during the long-term hydrogen exposure tests. Specifically, the Cu-plated/oxidized samples exhibit slightly better HPB characteristics than the oxidized sample (Table. r2). We confirmed that these curves do not intersect at longer times by normalizing these data using a same initial pressure (1.276 MPa).

As have been confirmed by the measurement of magnetic properties (Fig. 1), because the formation of Er-Co oxide layers also caused the decrease in magnetic entropy change (ΔS_M), simply oxidizing the $ErCo_2$ surface (without Cu plating) may not be sufficient for causing only a minimal decrease in magnetization and magnetic entropy change.

6. In lines 102 ~ 104, the authors infer that ErCo2 particles were fully penetrated and completely transformed into hydrides based solely on stereomicroscope observations of particle fragmentation. This conclusion may be premature, as morphological changes alone are insufficient to confirm complete hydrogenation.

Response: We revised the description of the $ErCo_2$ particles as follows.

"Stereomicroscopy reveal that the $ErCo_2$ particles pulverized after hydrogen exposure (Figs. S2a and S2b), which could be due to the large amount of absorbed hydrogen in $ErCo_2$."

7. Why weren't the magnetization and magnetic entropy change measured for the oxidized ErCo2 particles or those exposed to H2, as shown in Fig. 1c and 1d? These data would allow a clearer comparison with the as-homogenized and Cu-coated/oxidized samples, and help clarify how surface oxidation affects magnetocaloric performance.

Response: We have added the results of magnetization and magnetic entropy change measured for oxidized $ErCo_2$ before and after H_2 exposure test in Fig. 1, as also shown in Fig. r8. The necessary descriptions were added regarding those results.

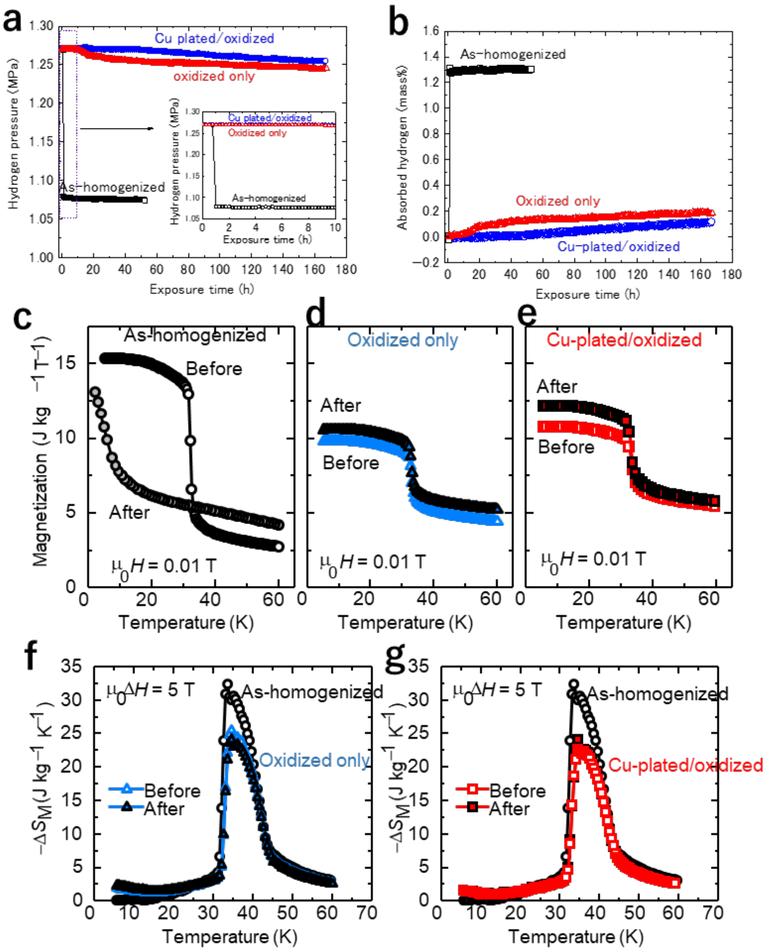

Fig. r8. **a.** Hydrogen pressure changes during the hydrogen exposure test for the as-homogenized, oxidized only, and Cu-plated/oxidized $ErCo_2$ particles. The hydrogen pressure change during the initial ten hours is inserted. **b.** Corresponding absorbed hydrogen amount calculated from the pressure changes. The hydrogen exposure test started with an initial hydrogen pressure in the cell of 1.27 MPa at approximately 296 K. **c.** Temperature dependence of magnetization at 0.01 T for the as-homogenized $ErCo_2$ particles. **d**. Temperature dependence of magnetization at 0.01 T for the oxidized $ErCo_2$ particles before and after hydrogen exposure test. **e**. Temperature dependence of magnetization at 0.01 T for the Cu-plated/oxidized $ErCo_2$ particles before and after hydrogen exposure test. **f.** Magnetic entropy change (ΔS_M) for $\mu_0 \Delta H$ = 5 T of the as-homogenized and oxidized $ErCo_2$ particles before and after the hydrogen exposure test. **g**. Magnetic entropy change (ΔS_M) for $\mu_0 \Delta H$ = 5 T of the as-homogenized and Cu-plated/oxidized $ErCo_2$ particles before and after the hydrogen exposure test (ΔS_M is not shown for the as-homogenized particles after the hydrogen

exposure test).

8. After the Cu plating–oxidation treatment on ErCo2, the magnetic entropy change (ΔSM) decreased significantly from 33 J·kg^{-1}·K^{-1} to 24 J·kg^{-1}·K^{-1}. What is the underlying reason for this reduction? Furthermore, is it possible to design surface treatments (e.g., coatings or modifications) that can preserve or even enhance the magnetic entropy change while still effectively preventing hydrogen embrittlement?

Response: As mentioned in our response to comment 2 of reviewer #1, the reduction in ΔS_M is due to the decrease of the weight fraction of $ErCo_2$ main phase in the $ErCo_2$ particle after Cu-plating and/or oxidation treatments. As the ΔS_M data in the present study are shown in unit of J per K per total mass of $ErCo_2$ particles, its magnitude decreases proportionally to the weight fraction of the $ErCo_2$ phase. From the microstructural observation data, we obtained an average thickness of each phase by measuring 3–5 particles and evaluated the weight fraction of each phase in the $ErCo_2$ particle: the innermost $ErCo_2$ phase; the Er–Co–O layer; the Co-O layer; and the outermost CuO layer (Fig. r2). The results show that the decrease of the weight fraction of the $ErCo_2$ phase is approximately 29% after Cu plating and oxidation, which roughly corresponds to the 30% decrease in magnetic entropy change. For the oxidized only sample, we also confirmed that the decrease of the weight fraction of the $ErCo_2$ phase corresponds to the decrease in magnetic entropy via the same process (Fig. r3). We have added the above explanation in the revised manuscript and Figs. r2 and r3 in the Supporting Information.

9. For the SEM (JEOL JSM-7000F, JSM-6500F) and TEM (JEOL JEM-ARM200F) characterizations coupled with X-ray EDS, could the authors please specify the accelerating voltage and beam current used?

Response: We have specified the accelerating voltage and beam current used for SEM and TEM in the revised manuscript.

10. The manuscript mentions that TOF-SIMS analysis was performed using a 30 kV Ga+ ion source for sputtering the surface of ErCo2 particles. However, the primary ion beam current was not reported. Could the authors clarify the beam current used

during analysis? This parameter critically affects sputtering rate, signal sensitivity, and surface damage, especially when analyzing non-planar particle surfaces.

Response: For the time-of-flight (ToF) measurements, we used the pulsed micro-beams of 30 keV Ga^+ ions with a pulse duration of 700 ps and repetition rate of 8.2 kHz. While the totally evaluated area of the samples was 100 μm^2, the area was divided to 256x256 segments and each of them was hit by the micro-beam of ~0.4 μm in diameter with scanning. Therefore, 65,536 segments of the sample were hit, but every part was hit only once per scanning of the evaluated area. The integrated beam current for each scanning was 8.5E10 ions/cm^2. To obtain a signal with good S/N, data, 23 scans were accumulated. The beam current of the data shown in Fig. 5 was 1.96E12 ions/cm^2. From the beam scanning, spatial mapping of elements was done before the measurements. We have confirmed that the evaluated area was homogenous and particle walls are free of cracks.

11. In the DFT calculations, the authors did not specify the energy convergence threshold and the force convergence criterion used during the slab model optimization. Similarly, the convergence settings applied in the CI-NEB (climbing image nudged elastic band) calculations were not given. It is recommended that the authors provide these critical computational parameters to ensure the reproducibility and reliability of the results.

Response: We have added in the Methods section the energy convergence threshold and the force convergence criterion used during the slab model optimization and for the CI-NEB.

"The state determination was performed using a convergence criterion of 5×10^{-6} eV for total energy and a force criterion of 0.01 eV/Å for force action."

"The activation energies for H_2 dissociation and atom diffusion on CuO(111) surface were determined using the climbing image nudged elastic band (CI-NEB) method[55]. The transition states were determined by interpolating 6 images between the initial and final states. The transition state determination was performed using a convergence criterion of 5×10^{-6} eV for total energy and a force criterion of 0.01 eV/Å for force action. The k-point mesh was 2×2×1. The cut-off energies were 450 eV for the wave function and 4050 eV for the charge density."

12. In line 187, the statement "forming an initial Co-rich oxide layer (Fig. S5)" seems incorrect, because the oxide layer is more likely Er-rich according to the EDS results shown in Fig. S5c.

Response: We revised the description of the oxide layer as "forming an oxide layer with a larger atomic ratio of Co/Er compared with that of subsurface region (Fig. S11)."

13. In line 230, the sentence "while the 59Co intensity remained at similar levels" appears to be inaccurate. According to Fig. 5c, the ToF-SIMS depth profile of 59Co shows an initial increase followed by a decrease as the analysis progresses into deeper layers. The authors are advised to revise this description to more accurately reflect the observed trend.

Response: We appreciate the reviewer's precise comments. We have revised the description to

"Moreover, the ^{59}Co intensity was at similar levels and the ^{167}Er signal was weak compared to their intensities at deeper layers (~40 μm)."

14. In Fig. 5b, there appears to be a typo in the y-axis label — "od" should likely be "of". The authors are advised to correct this for clarity and accuracy.

Response: We have corrected this mistake in Fig. 5b.

15. In lines 221–223, the statement "Beneath this layer, up to ~20 μm, the peak intensities of 167Er and 16O increased, while that of 59Co decreased" appears to be inaccurate. According to the ToF-SIMS depth profiles shown in Fig. 5b, the intensities of 167Er, 16O, and 59Co seem to remain relatively constant or slightly decrease in the 3–20 μm depth range. Please have the author confirm again.

Response: We appreciate the reviewer's comments on the ToF-SIMS data. We have corrected the description as follows:

"Beneath this layer, up to ~20 μm, the peak intensities of ^{167}Er, ^{59}Co, and ^{16}O were relatively

constant, whereas with further increase of the depth, the peak densities of ^{167}Er and ^{16}O decreased, while that of ^{59}Co remained constant,"

16. In lines 234–235, the authors state, "while the 167Er and 16O signal intensities remained constant up to ~20 μm." However, in Fig. 5c both 67Er and 16O intensities clearly show a gradual decline with increasing depth up to ~20 μm. Could the authors please clarify or correct this description so that it accurately reflects the observed trend?

Response: We compared the ^{167}Er, ^{59}Co, and ^{16}O signal intensities in Fig. 5c, and confirmed that the ^{167}Er and ^{16}O signal intensities remained constant up to ~20 μm while the ^{59}Co intensities gradually declined with increasing depth.

17. In Fig. 5d, the normalized D (deuterium) yield for the Cu-plated/oxidized ErCo2 particles shows fluctuations between approximately 15 μm and 26 μm depth, whereas one would expect the D signal to be zero beyond the oxidation layer. Could the authors please explain the origin of these D-signal fluctuations in that depth range and clarify why D is still detected where it should theoretically be absent?

Response: We have observed signals with the mass-number (MN) = 2 from samples without the deuteron exposure treatments. While the natural abundance of deuteron to proton is 1.5E-4, the intensity ratio of MN = 2 to MN = 1 (proton) was approximately 1/100. The MN=2 signal showed good correlation with the proton signal with the ratio of approximately 1/100. Therefore, the MN = 2 signal is inferred to consist of deuterium (D) and hydrogen molecules (H_2). The true deuterium amount was determined by subtracting 1/100 of the hydrogen atom signal intensity from the MN = 2 signal intensity. Without this subtraction, a non-negligible deuterium signal was observed even from samples not subjected to deuterium exposure treatment. We assumed the ratio of sputtered H_2 ion intensity to H ion intensity was constant at 1/100, though this ratio could vary depending on factors such as the sputtering of surface composition. The presence of H_2 molecules prevents the precise determination of D concentration. Negative D values observed at specific depths are considered within the error range.

18. In the DFT calculations, only the adsorption and dissociation of a single H2 molecule on the CuO(111) surface were considered. However, under experimental conditions multiple H_2 molecules adsorb, dissociate, and diffuse concurrently, leading to coverage-dependent interactions. Could the authors discuss how multi-molecule effects (e.g., surface coverage, H–H lateral interactions, and diffusion pathways) might alter the adsorption/dissociation energetics? Would considering cluster models, larger supercells, or molecular dynamics simulations be necessary to capture these effects?

Response: The effects of H_2 surface coverage on H_2 adsorption structure on CuO (111) surface were examined by setting various surface coverage rates of H_2 molecule on CuO(111) surface (0.0625–1 ML) (Fig. r9). The slab model and calculation conditions for these surface coverage effects were the same as that for the single H_2 molecule on the CuO(111) surface. In the initial state, the H_2 molecules were placed approximately 1.5 Å above each outermost O or Cu atom. The results revealed that H_2 molecules moved away from the surface at all surface coverage rates (0.0625–1 ML).

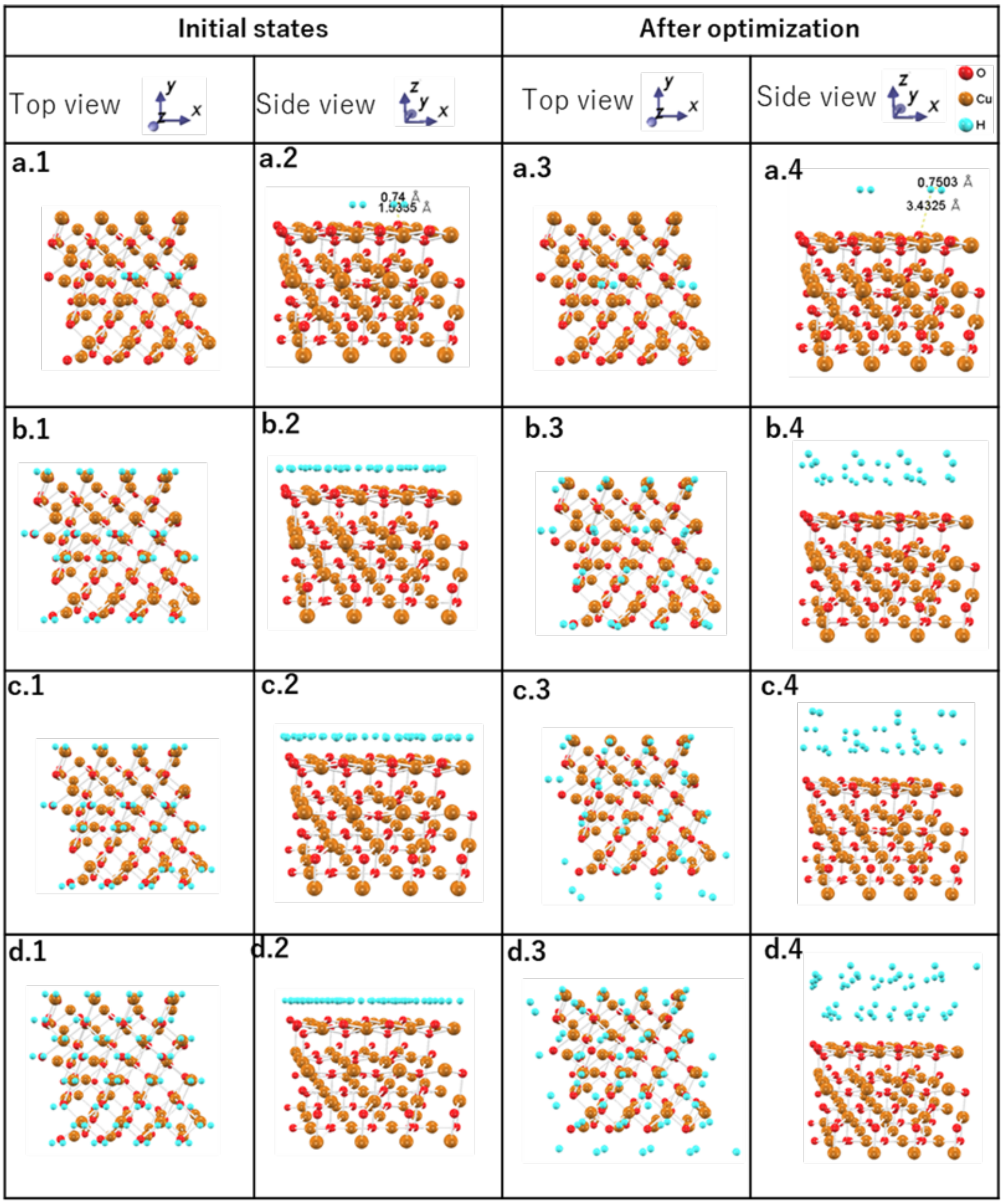

Fig. r9. Effect of H_2 surface coverage on H_2 adsorption structure on CuO (111) surface. **a.1–4**: 0.0625 ML, **a.1** (top view) and **a.2** (side view): initial state, **a.3** (top view) and **a.4** (side view): after optimization; **b.1–4**: 0.5 ML, **b.1** (top view) and **b.2** (side view): initial state; **b.3** (top view) and **b.4** (side view): after optimization; **c.1–4**: 0.5625 ML, **c.1** (top view) and **c.2** (side view): initial state; **c.3** (top view) and **c.4** (side view): after optimization; **d.1–4**: 1.0 ML, **d.1** (top view) and **d.2** (side view): initial state; **d.3** (top view) and **d.4** (side view): after optimization. The initial distance between H_2 and CuO(111) surface was approximately 1.5 Å, and the distance between H atoms in H_2 molecule was approximately 0.74 Å. After optimization, the distance between H_2 and CuO surface was larger than 2.8 Å, and the distance between H atoms in H_2 molecule

was approximately 0.75 Å.

The effect of H surface coverage on H adsorption structure on CuO (111) surface were examined by setting various surface coverage rates of H atom on CuO(111) surface (0.0625–1 ML) (Fig. r10). The slab model and calculation conditions for these surface coverage effects were the same as that for the single H_2 molecule on the CuO(111) surface. In the initial state, the H atoms were placed at positions approximately 1.5 Å above each outermost O or Cu atom. The results revealed that H atoms most readily adsorb onto O^3 atoms, followed by Cu^3 atoms. However, when the H surface coverage exceeds 0.5 ML, additional H atoms can no longer adsorb onto the surface. They detach from the surface and form hydrogen molecules. The adsorption energy of a H atom on the CuO (111) surface was calculated at various H coverage rates, indicating that the adsorption energy decrease with the increase of H coverage (Fig. r11).

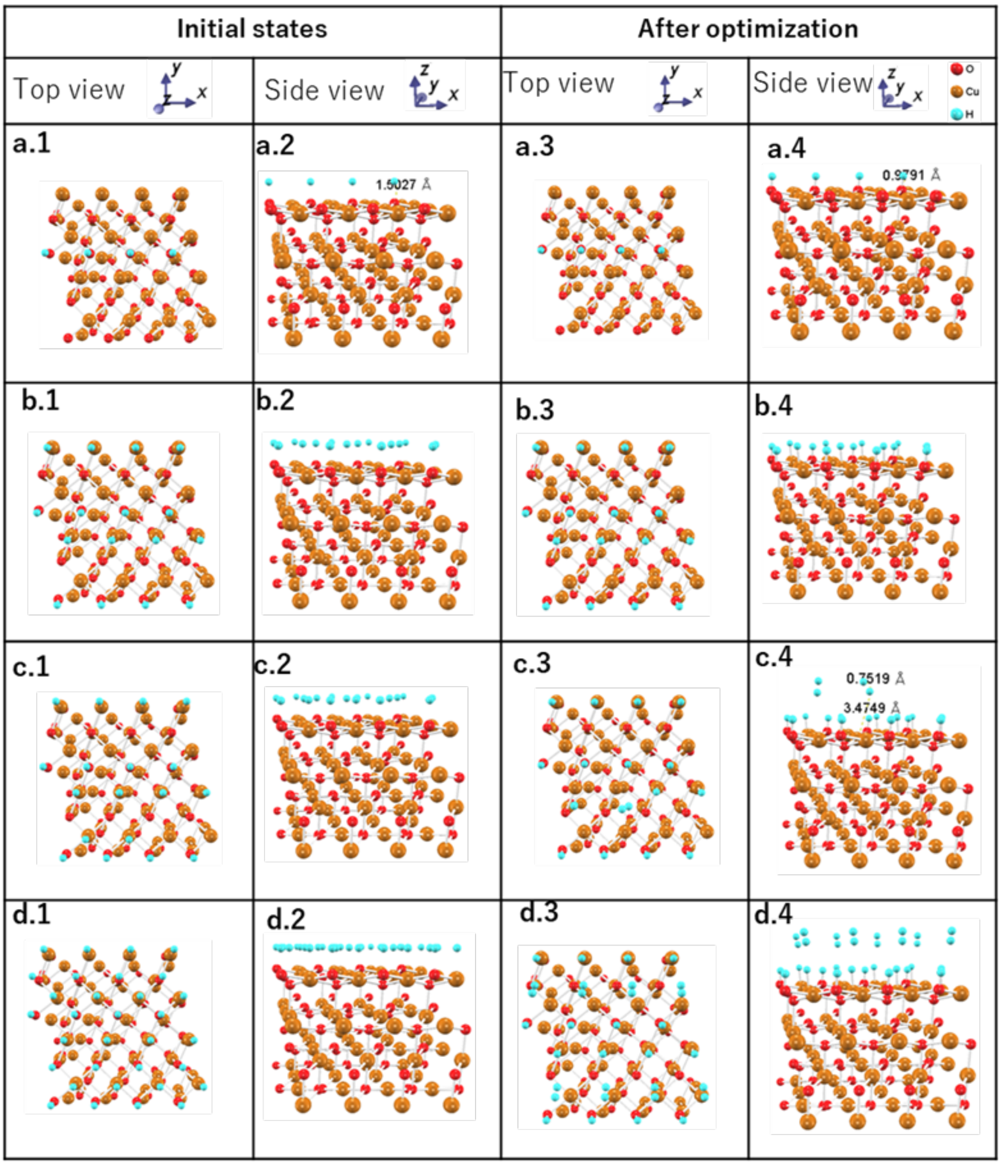

Fig. r10. Effect of H surface coverage on H adsorption on CuO (111) surface. **a.1–4**: 0.0625 ML, **a.1** (top view) and **a.2** (side view): initial state, **a.3** (top view) and **a.4** (side view): after optimization; **b.1–4**: 0.5 ML, **b.1** (top view) and **b.2** (side view): initial state; **b.3** (top view) and **b.4** (side view): after optimization; **c.1–4**: 0.5625 ML, **c.1** (top view) and **c.2** (side view): initial state; **c.3** (top view) and **c.4** (side view): after optimization; **d.1–4**: 1.0 ML, **d.1** (top view) and **d.2** (side view): initial state; **d.3** (top view) and **d.4** (side view): after optimization. The initial distance between H and CuO surface was set as approximately 1.5 Å, and the H atoms were preferentially set on the top of O^3 and Cu^3 atoms, and then on the top of O^4 and Cu^4 atoms. After optimization, the distance between the H and the O^3 atom was approximately 0.98 Å, and the distance between the H atom and the Cu^3 atom was approximately 1.5 Å.

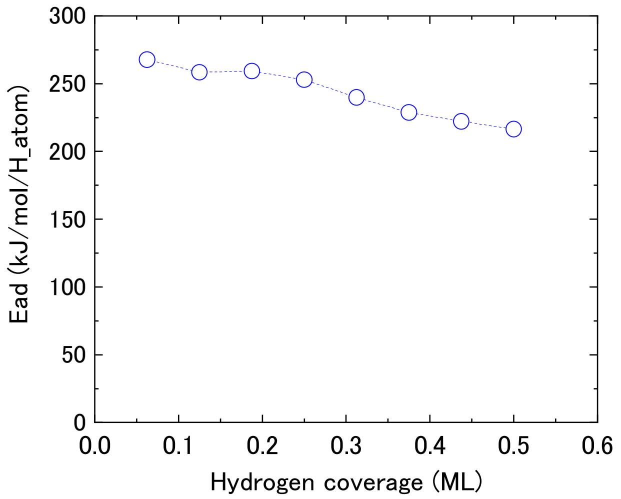

Fig. r11. Adsorption energy of a H atom on the CuO (111) surface as a function of H coverage (ML).

We have added the following descriptions in the manuscript and Figs. R9–11 in the Supplementary Information based on the above results.

"The effects of H_2 molecule surface coverage on H_2 adsorption structure on CuO (111) surface were examined by setting various surface coverage rates of H_2 molecule on CuO(111) surface. The results revealed that H_2 molecules moved away from the surface at all surface coverage rates (0.0625–1 ML, Fig. S14). The effect of H-atom surface coverage on H adsorption structure on CuO(111) were also examined by setting various surface coverage rates of H atom on CuO(111) surface. The results revealed that H atoms most readily adsorb onto O^3 atoms, followed by Cu^3 atoms with the increase of H coverage (Fig. S15). The adsorption energy of a H atom on the CuO (111) surface decreased with the increase of H coverage up to 0.5 ML (Fig. S16). Furthermore, when the H surface coverage exceeds 0.5 ML, additional H atoms can no longer adsorb onto the surface. They detach from the surface and form hydrogen molecules (Fig. S15)."

19. To accurately predict H_2 adsorption and dissociation on CuO(111), zero-point energy corrections should be included.

Response: We have revised the energy values by including the zero-point energy (ZPE) corrections. The following description for the calculation of ZPE was added in

the Methods section.

"The zero-point energy (ZPE) correction was performed by calculating vibration modes using the linear response function of PHASE0 after optimizing the slab model and each adsorption state, and the ZPE energy was calculated as follows:

$$ZPE = \frac{1}{2}\sum_{i=1}^{n}(h\gamma_i) \quad (4)$$

where γ_i is the frequency of each actual value vibration mode, n is the total numbers of actual value vibration mode, and h is Planck constant ($4.135667696\times10^{-15}$ eV·s). By this method, the ZPE value of free H_2 molecule was calculated to be 0.273436 eV, which agrees well with the literature value[54]."

20. In the subtitle of Fig. 6a, the phrase "Optimized positions of H_2 molecules at all initial adsorption sites (shown in Fig. S4a)" appears to be incorrect—shouldn't it refer to Fig. S6a instead? Additionally, the wording "showing that all H_2 molecules moved away from the CuO(111) surface, to a distance exceeding 2.6 Å" seems inconsistent with the data, as the distances actually exceed 2.8 Å. Please verify and correct these details.

Response: We are very grateful for the reviewer's precise comments. We have revised the Fig. number, and the typo from "2.6 Å" to "2.8 Å".

21. Three molecular orientations were considered: one perpendicular to the CuO(111) surface and two parallel, but the tilted side-on orientation was not examined. Could the authors comment on whether this tilted geometry might be energetically favorable, and if so, please provide its adsorption energy and optimized structure.

Response: We have examined the tilted side-on orientation (approximately 45° tilted from side-on orientation.) and added the results in the manuscript and Supplementary information (Fig. S12, Table S1–2).

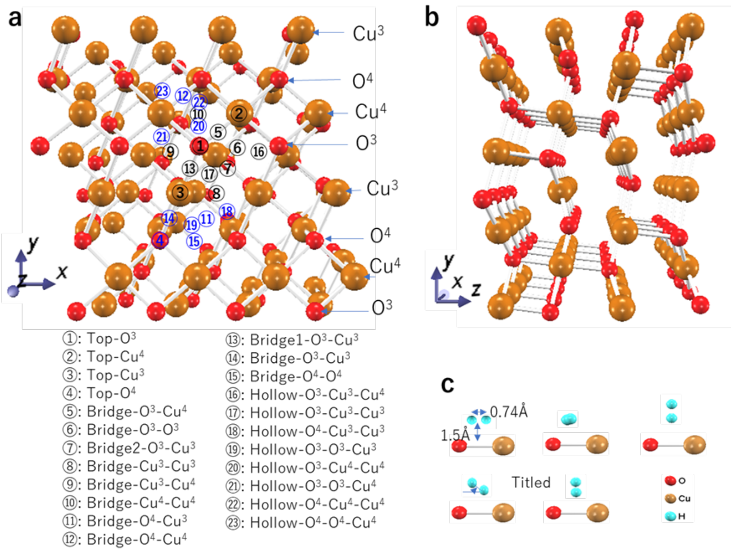

Fig. r12. Various adsorption sites on the slab model of a p(4×2) CuO(111) surface. **a**. top-view. **b**. left-view. **c**. five orientations of the H_2 molecules set in the initial state: one perpendicular to the surface; two parallel to the surface (at the bridge site, they were parallel or perpendicular to the bridge and at the hollow site, they were parallel to the x- or y-axis); and two tilted approximately 45° toward the z-axis from the two orientations parallel to the surface. The interatomic distance of H_2 molecules was set to be 0.74 Å, and the distance between the hydrogen molecules and the CuO(111) surface was set to about 1.5 Å in the initial state. O^3 – three-coordinated O atom; O^4 – four-coordinated O atom; Cu^3 – three-coordinated Cu atom; Cu^4 – four-coordinated Cu atom.

22. In lines 312~314, for the statement "For each measurement, 0.5 g of particles was used. Hydrogen gas was introduced at an initial pressure of ~1.27 MPa after vacuuming with a rotary pump for at least 15 minutes," could the authors clarify whether the samples were first purged with an inert gas to remove residual air? Additionally, what vacuum pressure was reached before introducing hydrogen?

Response: We did not purge the cell with an inert gas. The vacuum pressure was <0.001 MPa before introducing hydrogen. The minimum display of the pressure gauge in our measurement system is 0.001 MPa. Values below this are displayed as 0.000, and the specific value is not shown.

23. In the caption of Fig. 6, the description for subfigure (c) is missing.

Response: We added the description for subfigure 6(c) in the revised manuscript.

24. DFT calculations indicate a high barrier for H diffusion into the CuO(111) subsurface. However, the authors could also calculate hydrogen solubility in the CuO layer to show that it is low, further supporting its enhanced resistance to hydrogen embrittlement.

Response: We have evaluated the hydrogen atom solid solubility limit in CuO by calculating the solution energies when inserting H atoms sequentially up to 9 atoms into interstitial octahedral sites in a larger slab model comprising 256 atoms (Fig. r13). Structural optimization of the slab model was first performed with all Cu and O atoms movable. Subsequently, the total energies of each state with solid solution H atoms were calculated by fixing the Cu and O atoms in the optimized model, and only the H atoms being movable (Figs. r14 and r15). The Brillouin zone was sampled using a 1×2×3 k-point mesh, with cut-off energies of 450 eV for the wave function and 1800 eV for the charge density. The state determination was performed using a convergence criterion of 1×10^{-5} eV for total energy and a force criterion of 0.01 eV/Å for force action. The solution energy for solid solution of H atoms into CuO (ΔG_{sol}) was calculated as follows:

$$\Delta G_{sol} = (E_{(CuO+nH)} + ZPE_{(CuO+nH)}) - (E_{CuO} + ZPE_{CuO}) - \frac{n}{2}(E_{H_2} + ZPE_{H_2} + \mu_{H_2}(T,p)) \quad (6)$$

where n is the numbers of H atom (n = 1–9), $E_{(CuO+nH)}$ and $ZPE_{(CuO+nH)}$ are the total energy and ZPE of CuO with solubilized H atoms, E_{CuO} and ZPE_{CuO} are the total energy and ZPE of CuO, E_{H_2} and ZPE_{H_2} are the total energy and ZPE of free H_2 molecule, and $\mu_{H_2}(T,p)$ is the chemical potential of gaseous H_2 contributed by temperature (T) and pressure (p) as follows.

$$\mu_{H_2}(T,p) = \mu_{H_2}(T,p^{\circ}) + k_B T ln(\frac{p}{p^{\circ}}) \quad (7)$$

The H concentration (c_H) was estimated as follows.

$c_H \approx \exp\left(-\frac{\Delta G_{sol}}{k_B T}\right)$

(8)

where k_B is Boltzmann's constant (1.380649×10^{-23} J/K), T is absolute temperature (K), $p°$ is the reference pressure, and p is the actual pressure. $\mu_{H_2}(T, p°)$ is the chemical potential of H_2 at the reference pressure (1 atm) which were obtained from the NIST standard reference database (https://doi.org/10.18434/T4D303). The H solubility at ΔG_sol = 0 was determined as 0.01377 at% by extrapolation, indicating an extremely low H solubility in CuO at 298 K and 1.27 MPa.

We have added the detailed calculation method in the Method and the description of results in the text and Supplementary information (Fig. S).

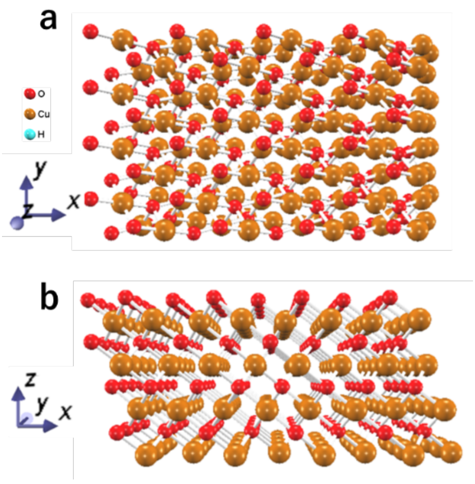

Fig. r13. Schematic representation of the CuO slab model consisted of 256 Cu and O atoms (32×8) used for hydrogen solution. **a**. top view; **b**. side view.

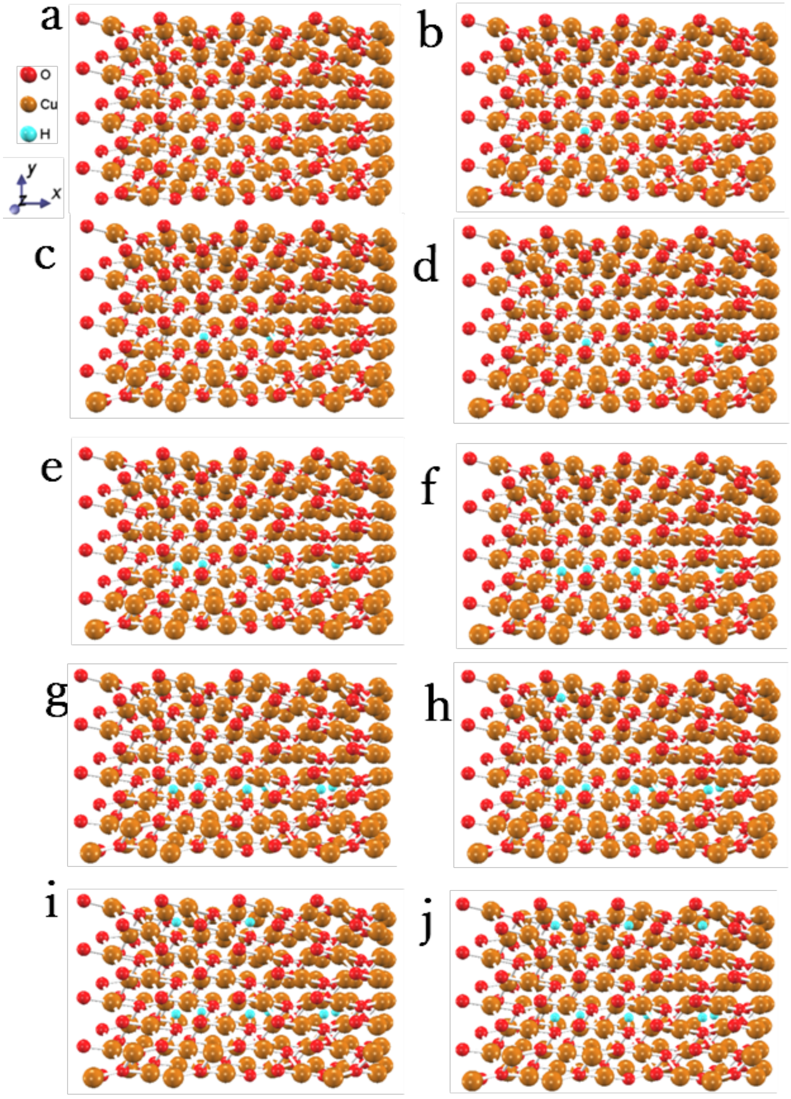

Fig. r14. Optimized structures of CuO with H atoms inserted into interstitial octahedral sites. **a.** CuO slab model consisted of 256 Cu and O atoms before inserting H; **b.** CuO with inserting one H; **c.** CuO with two H inserting; **d.** CuO with inserting three H; **e.** CuO with inserting four H; **f.** CuO with inserting five H; **g.** CuO with inserting six H; **h.** CuO with inserting seven H; **i.** CuO with inserting eight H; **j.** CuO with inserting nine H.

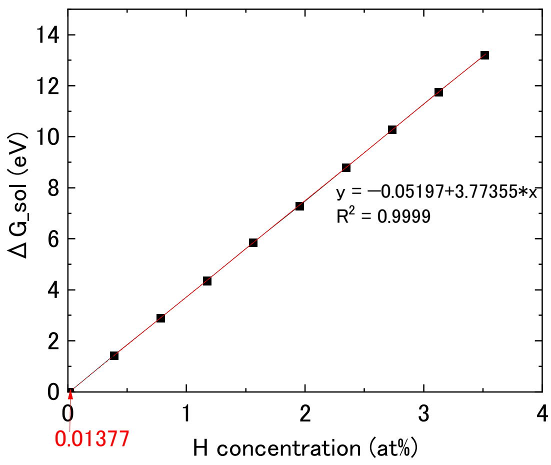

Fig. r15. Solubility energy (ΔG_sol) at various hydrogen concentrations in CuO at 298 K and 1.27 MPa. These values exhibit excellent linearity. The H solubility at ΔG_sol = 0 was determined as 0.01377 at% by extrapolation.

Reviewer #4 (Remarks to the Author):

This paper reported the formation of oxide layer on the surface of ErCo2 sphere as hydrogen permeation barrier. By comparing the performance of CuO layer and the in situ generated oxide layer of ErCo2, the authors found that CuO is more efficient for hydrogen permeation protection. This finding may provide some valuable insights for protecting the magnetic refrigeration materials from hydrogen embrittlement. But there are still some questions needed to be further clarified.

1. Some important content that can emphasize the novelty of this work is suggested to be included as follows

1)Is the hydrogen permeation issue a key challenge faced by the magnetic refrigeration materials?

Response: We appreciate the reviewer's insightful and valuable comments, which helped improve the quality of our manuscript. To date, magnetic refrigeration systems have been placing magnetic refrigeration materials in a helium gas atmosphere rather than a hydrogen gas atmosphere, attempting hydrogen liquefaction via helium gas flow (Ref. 6). If magnetic refrigeration materials can be

endowed with superior HPB performance, they could be used directly in a hydrogen atmosphere, promising system simplification and significant efficiency improvements. We have added this discussion in the Introduction.

"Currently, magnetic refrigeration systems use a helium gas atmosphere rather than a hydrogen gas atmosphere to liquefy hydrogen via helium gas flow[6].
The AMR refrigeration system could be simplified and the energy efficiency significantly improved if magnetic refrigeration materials could be placed directly in a hydrogen atmosphere."

2)Are there any other reports on developing the hydrogen permeation barrier for magnetic refrigeration material? If so, how about their performance?

Response: To the best of our knowledge, no research has been conducted on the hydrogen barrier properties of magnetic refrigeration materials.

3)The background for choosing CuO instead of other materials as the hydrogen permeation barrier should be provided, as there are many materials have been reported as efficient hydrogen permeation barriers, such as ZrO2, Er2O3, α-Al2O3, CrxN, et al. These materials have higher chemical stability in the hydrogen environment than CuO. What is the principle for choosing the alternative hydrogen resistant materials for ErCo2? Except the hydrogen permeation resistance, are there any other characteristics or functional properties required by the barriers?

Response: As mentioned in our response to comment 1 from reviewer #3, we tried to use materials with low hydrogen diffusivity, such as Al_2O_3 and Cr_2O_3, for forming a HPB layer on magnetic refrigeration materials. We asked a company to form a Cr_2O_3 layer on $ErCo_2$ particles via thermal spraying, however, it was proved difficult to prevent particle aggregation and to control the uniformity of the coating layer on the small $ErCo_2$ particles via thermal spraying (Fig. r4). Furthermore, we commissioned a company specializing in PVD to form an Al_2O_3 layer onto the $ErCo_2$ particles via a barrel PVD method. $ErCo_2$ particles were placed within a multi-faceted barrel which was rotated during the coating process. However, it was difficult to form Al_2O_3 layer uniformly on the surface of small $ErCo_2$ particles with significant surface irregularities. Compared to these coating methods, copper plating followed by oxidation treatment offers great advantages in terms of cost and efficiency. CuO has not previously been

recognized as an HPB material, and this study has demonstrated for the first time that it possesses sufficient HPB properties near room temperature. We have added this discussion in the Introduction.

2.As the hydrogen permeation barrier performance is evaluated by the hydrogen pressure measured in the equipment. The accuracy of the pressure gauge is very important, as the changes of the hydrogen pressure mentioned in the work are 0.01~0.02 MPa, the type of the pressure gauge used and its Instrument detection limit, the average relative error should be given.

Response: We added the information of the pressure gauge used in the revised manuscript.
"The average relative error of the pressure gauge (PG-50KU-F, KYOWA Electronic Instruments Co., Ltd., Japan) is ±0.5%. The calibration sheet of the gauge shows its rate output is 1997 μV/V, and the calibration constant is 0.00245 MPa/ 1μV/V."

3.It seems that ΔSM was decreased with CuO layer on the sphere surface, is the thickness of CuO layer affect the ΔSM? Can the CuO thickness be controlled by the plating-oxidation method? If thinner the layer, will the ΔSM have a higher value?

Response: We appreciate the reviewer's insightful comment. As mentioned in our response to comment 2 of reviewer #1 and comment 8 of reviewer #3, the ΔS_M decreased not only by the CuO layer but also by the Er-Co oxide layers on the spherical surface, and the weight of Er–Co oxide layers were approximately four times that of the CuO layer (Figs. r2 and r3). Therefore, ΔS_M should have a higher value if the CuO layer is thinner. However, simply reducing the thickness of the CuO layer may not be sufficient to prevent a decrease in ΔS_M.

4.The authors have tested the performance of the hydrogen permeation barriers under 1.27 MPa H2 at 296 K for 7 days. Is this pressure, temperature and time agreed with the condition faced by ErCo2 spheres when in practical applications? What is biggest pressure and the longest time for the ErCo2 spheres exposure to H2 toward hydrogen liquefaction? And how about the performance of CuO layer under higher pressures or for expanding times?

Response: As we have mentioned in our response to comment 2 of reviewer #3, gases

at 1 MPa or higher are classified as high-pressure gases and fall under the jurisdiction of Japan's High-Pressure Gas Safety Act. Magnetic refrigeration systems offer practical advantages by avoiding this classification. In fact, current magnetic refrigeration systems are designed to operate at pressures below 1 MPa. Therefore, the initial pressure of 1.27 MPa we selected is sufficient for current practical magnetic refrigeration systems. However, higher pressures are advantageous for improving energy efficiency and should be a future research topic.

$ErCo_2$ is usually used in magnetic refrigeration systems at 20–77 K, except for routine maintenance at room temperature. The routine maintenance period of magnetic refrigeration system is usually a few days per year. Therefore, the condition used in this study (1.27 MPa H_2 at 296 K for 7 days) agree with the condition in practical application. Furthermore, we have confirmed that the Cu-plated/oxidized $ErCo_2$ can even maintain good HPB performance under 1.27 MPa H_2 for up to 28 days at 293–297 K (Fig. r4). The PCT measurements indicate that the Cu-plated/oxidized $ErCo_2$ may maintain good HPB performance at higher H_2 pressure (4 MPa) and higher temperature (333 K) (Fig. r3).

5.It is mentioned that the pre-cleaning step was essential for ensuring uniform Cu deposition. What is the reason? A detailed discussion about this phenomenon is absent. As the quality of the CuO layer is the key to determine its performance, therefore, the growth mechanism of Cu on the sphere surface and the method to improve its quality should be demonstrated in the text.

Response: We have compared the Cu plated $ErCo_2$ particles without and with pre-cleaning. Fig. r16 show the stereomicroscope observations of particles with and without pre-cleaning. A part of the particles could not be Cu-plated without pre-cleaning. SEM and EDS analyses revealed that a very thin Er-Co oxide layer existed on the surface of as-homogenized $ErCo_2$ particles. This natural oxide layer is thought to be formed during the homogenization heat treatment process or subsequent storage at room temperature in air. This oxide layer is believed to affect the copper plating process and need to be removed by pre-cleaning. We have added the related description in the manuscript.

"SEM and EDS analyses revealed that a very thin Er–Co oxide layer existed on the surface of as-homogenized $ErCo_2$ particles, formed during the homogenization heat treatment process or subsequent storage at room temperature in air. This oxide layer

is believed to affect the copper plating process and need to be removed by pre-cleaning."

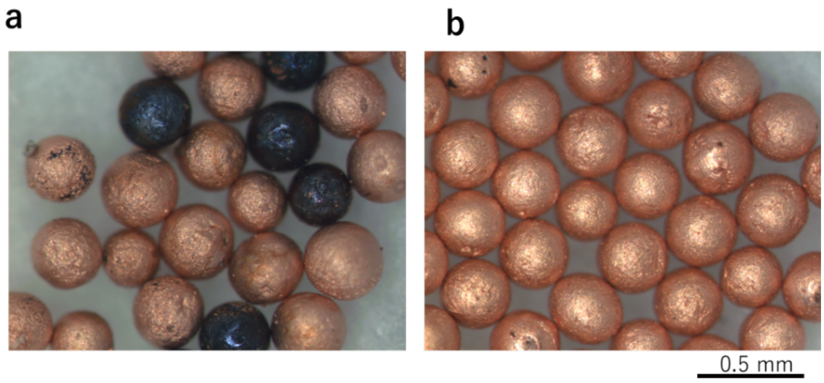

Fig. r16. Stereomicroscope observation of Cu plated $ErCo_2$ particles without (**a**) and with (**b**) pre-cleaning by immersion in 4 vol% HCl aqueous solution at 298 K for 20 seconds.

6.In DFT calculations, line 274 "suggesting that H atoms diffuse easily on the CuO surface", this conclusion cannot be easily understand. And how could the authors get the result "the energy barrier for H diffusion from the surface to subsurface was 337.4 kJ/mol"? These results need to be clarified more clearly in order to be understandable for the readers.

Response: We have revised the description in line 274 to:
"In contrast, the diffusion barrier for H migration from H–Cu to H–O was much lower (13.5 kJ/mol) (Fig. 6e), suggesting that H atoms diffuse easily on the CuO surface."

We have added the following description to the energy barrier value in the revised text.

"Moreover, the energy barrier for H diffusion from the surface to subsurface was 337.4 kJ/mol (obtained from the difference between the total energy of TS (240.8 kJ/mol) and that of the H–O/H–O absorbed surface state (–96.6 kJ/mol, Fig. 6e),"

Reviewer #5 (Remarks to the Author):

I co-reviewed this manuscript with one of the reviewers who provided the listed

reports. This is part of the Nature Communications initiative to facilitate training in peer review and to provide appropriate recognition for Early Career Researchers who co-review manuscripts.

RESPONSE TO REVIEWERS' COMMENTS

Reviewer #1 (Remarks to the Author):

The authors have addressed all the questions raised before. I would like to recommend the revised manuscript for publication in Nature Communications.

Response: We sincerely appreciate Reviewer #1's thorough and constructive comments. These have been extremely helpful in improving the quality of our manuscript.

Reviewer #2 (Remarks to the Author):

The authors responded appropriately to my comments. Please find attached the manuscript, where I have marked the incorrect names of compounds in red.

Response: We sincerely appreciate Reviewer #2's insightful and constructive comments. These have been extremely helpful in improving the quality of our manuscript. We have corrected the names of the compounds the reviewer pointed out. We apologize for these errors.

(Lines 43-44)
$DrNi_2$ ➔ $DyNi_2$
$Dr_{1-x}Er_xNi_2$ ➔ $Dy_{1-x}Er_xNi_2$
(Line 46)
HoB_2 ➔ $HoNi_2$

Reviewer #3 (Remarks to the Author):

In the manuscript titled "Enhancing hydrogen permeation barrier performance of ErCo2 magnetic refrigeration material via surface oxide layer formation," the authors reveal, through experimental analyses and first-principles calculations, the CuO layer serves as an effective hydrogen permeation barrier, enhancing the hydrogen resistance of $ErCo_2$ intermetallic compounds for magnetic refrigeration applications. The revised manuscript shows clear improvement after the first-round revision. However, several technical and formatting issues still need to be addressed.

(1) The “DFT calculation(s)” section contains font size inconsistencies and apparent character encoding errors, likely related to equation formatting.
(2) The authors should carefully correct these issues and conduct a thorough check of the entire manuscript to ensure consistency in formatting, figure captions, symbols, overall presentation, and figure resolution.

Response: We sincerely appreciate Reviewer #3’s insightful and constructive comments. These have been extremely helpful in improving the quality of our manuscript.

(1) We have corrected inconsistent font sizes and apparent character encoding errors in the “DFT Calculation” section. We sincerely apologize for these errors.
(2) We have conducted a thorough check to ensure consistency in the formatting of the entire manuscript, figure captions, symbols, overall presentation, and figure resolution.

Reviewer #4 (Remarks to the Author):

The authors replied the reviewers’comments and carried out additional experiments and calculations. The reviewer agrees for its publication.

Response: We sincerely appreciate Reviewer #4’s insightful and constructive comments. These have been extremely helpful in improving the quality of our manuscript.

Reviewer #5 (Remarks to the Author):

I co-reviewed this manuscript with one of the reviewers who provided the listed reports. This is part of the Nature Communications initiative to facilitate training in peer review and to provide appropriate recognition for Early Career Researchers who co-review manuscripts.

Response: We sincerely thank Reviewer #5 for the collaborative review of this manuscript. It is extremely valuable in improving the quality of the manuscript.

Hydrogen liquefaction is crucial for its transportation and storage[1-3]. Magnetic refrigeration offers a more energy-efficient alternative to conventional gas compression/expansion refrigeration technology[4-7], and the efficiency of such refrigeration systems can be further improved using magnetic refrigeration materials with large magnetocaloric effects. Numerous magnetic refrigeration materials with large magnetocaloric effects have been developed, such as $DrNi_2$[8], $ErCo_2$[9-11], $ErNi_2$[12], $Dr_{1-x}Er_xNi_2$[13], HoB_2[14], and $HoAl_2$[15]. However, the development of magnetic refrigeration materials with a large magnetic entropy change (ΔS_M) over a broad temperature range (77–20 K) is challenging[16-20]. A layered composite magnetic refrigerant of HoB_2, $DyNi_2$,
